# Screening for depression among the general adult population and in women during pregnancy or the first-year postpartum: two systematic reviews to inform a guideline of the Canadian Task Force on Preventive Health Care

**DOI:** 10.1186/s13643-022-02022-2

**Published:** 2022-08-22

**Authors:** Andrew Beck, Candyce Hamel, Micere Thuku, Leila Esmaeilisaraji, Alexandria Bennett, Nicole Shaver, Becky Skidmore, Ian Colman, Sophie Grigoriadis, Stuart Gordon Nicholls, Beth K. Potter, Kerri Ritchie, Priya Vasa, Beverley J. Shea, David Moher, Julian Little, Adrienne Stevens

**Affiliations:** 1 Clinical Epidemiology Program, Ottawa Hospital Research Institute, Ottawa, Ontario Canada; 2 School of Epidemiology and Public Health, Faculty of Medicine, University of Ottawa, Ottawa, Ontario Canada; 3 Department of Psychiatry, University of Toronto, Toronto, Canada; 4 Sunnybrook Health Sciences Centre, Toronto, Canada; 5 Ottawa Hospital Research Institute, Ottawa, Ontario Canada; 6 School of Psychology, University of Ottawa, Ottawa, Ontario Canada; 7 Department of Family and Community Medicine, St. Michael’s Hospital, University of Toronto, Toronto, Ontario Canada

**Keywords:** Depression, Screening, Systematic review, Adults, Pregnancy, Postpartum

## Abstract

**Background:**

Depression affects an individual’s physical health and mental well-being and, in pregnant and postpartum women, has specific adverse short- and long-term effects on maternal, child, and family health. The aim of these two systematic reviews is to identify evidence on the benefits and harms of screening for depression compared to no screening in the general adult and pregnant and postpartum populations in primary care or non-mental health clinic settings. These reviews will inform recommendations by the Canadian Task Force on Preventive Health Care.

**Methods:**

We searched MEDLINE, Embase, PsycINFO, CINAHL, and the Cochrane Library using a randomized controlled trial filter, where applicable, October 4, 2018, and updated to May 11, 2020. We also searched for gray literature (e.g., websites of organizations of health professionals and patients). Study selection for depression screening trials was performed first on title and abstract, followed by full-text screening. Data extraction, assessment of the risk of bias using the Cochrane risk of bias tool, and application of Grading of Recommendations Assessment, Development and Evaluation were performed by one reviewer and validated by a second reviewer.

**Results:**

A total of three trials were included. All three trials were included in the general adult review, while one of the three trials was included in the pregnant and postpartum review. We did not pool results due to substantial differences between studies and high risk of bias. In the general adult review, the first trial (*n* = 1001) evaluated whether screening for depression in adults with acute coronary syndrome compared to usual care improves health-related quality of life, depression symptoms, or harms of screening at 6, 12, and 18 months. There were little to no differences between the groups at 18 months for the outcomes. The second trial included adults (*n* = 1412) undergoing initial consultation for osteoarthritis, evaluated for depression and general health (mental and physical) after initial consultation and at 3, 6, and 12 months. The physical component score was statistically significantly lower (worse health) in the screened group at 6 months; however, this difference was not significant at 3 or at 12 months. There were no clinically important or statistically significant differences for other outcomes between groups at any time. The third trial (included in both reviews) reported on 462 postpartum women. At 6 months postpartum, fewer women in the screening group were identified as possibly depressed compared to the control group (RR 0.59, 95% confidence interval (CI) 0.39 to 0.89) and mean EPDS scores were also statistically significantly lower in the screened group (standardized mean difference 0.34 lower (95% CI 0.15 to 0.52 lower)). All other outcomes did not differ between groups at follow-up. There were serious concerns about the cut-offs used for the questionnaire used to screen, diagnostic confirmation, selective outcome reporting, and the reported magnitude of effects.

**Discussion:**

There are limitations of the evidence included in the reviews. There was moderate certainty in the evidence from one trial that screening for depression in the general adult population in primary care or non-mental health clinic settings likely results in little to no difference on reported outcomes; however, the evidence was uncertain from the other two included trials. The evidence is very uncertain about the effect of screening for depression in pregnant or postpartum women in primary care or non-mental health clinic settings. Well-conducted and better-reported trials are needed that meet the screening trial criteria used in this review.

**Systematic review registration:**

Both protocols have been registered in the International Prospective Registry of Systematic Reviews (PROSPERO) [adult: CRD42018099690; pregnancy and postpartum: CRD42018099689﻿] and published (https://systematicreviewsjournal.biomedcentral.com/track/pdf/10.1186/s13643-018-0930-3).

**Supplementary Information:**

The online version contains supplementary material available at 10.1186/s13643-022-02022-2.

## Introduction

### Rationale

Since the release of the 2013 Canadian Task Force on Preventive Health Care (“Task Force”) guideline on depression screening in the general adult population [1, 2], other guidelines have been updated; however, the recommendations for screening are discordant. Neither the Task Force nor the UK National Screening Committee (UK NSC) [3] recommended routinely screening the adult population in contrast to the 2016 US Preventive Services Task Force (USPFTF) recommendation for screening when adequate systems were in place to ensure accurate diagnosis, effective treatment, and appropriate follow-up [4, 5]. The 2013 Task Force guideline also considered the perinatal and postpartum population as a subgroup who may be at increased risk of depression and did not recommend routine screening which was also similar to the UK NSC postnatal depression screening recommendation, last updated in 2011 [3]. In contrast, the 2015 guideline from the American College of Obstetricians and Gynecologists recommended screening patients at least once during the perinatal period for depression and anxiety symptoms using a standardized, validated tool, despite limited evidence of benefit [6, 7]. The 2016 USPSTF also recommended screening pregnant and postpartum women [4, 5].

Due to newer and discordant recommendations since the 2013 Task Force guideline on depression screening among the general adult population, the Task Force decided to update their 2013 guideline and develop an additional guideline and systematic review considering women during pregnancy and postpartum.

### Background

Depression is a mood disorder characterized by states of low mood, feelings of hopelessness, worthlessness, or emptiness and accompanied by physical symptoms such as decreased activity, poor appetite, and poor sleep, persisting for at least 2 weeks and serious enough to impair functioning in social, occupational, educational, or other situations [8]. The current definition of a major depressive episode (MDE) is based on one of two classifications [9]: Diagnostic and Statistical Manual of Mental Disorders, Fifth Edition (DSM-5) [10] and International Statistical Classification of Diseases and Related Health Problems, 10th revision (ICD-10) [11]. As of 2018, it was estimated that over 264 million people worldwide live with depression, making it the most common cause of disability worldwide [12].

The 2012 Canadian Community Health Survey-Mental Health evaluated 25,113 individuals (aged 15 years and older) and reported an annual prevalence of major depressive disorder (MDD) of 3.9% (95% confidence interval (CI) 3.5–4.2%) [13]. Depression can affect work performance through absenteeism and presenteeism (decreased work productivity while at work), which is a large cost to employers in terms of productivity [9]. On a population level, it also has a large societal impact through increased health service utilization, increased burden on family members, and increased resource costs related to disability [14]. A US study in which women were interviewed (*n* = 14,549), and diagnosed using the DSM-IV criteria, found the 12-month period prevalence of MDD to be 8.4% among women who were currently pregnant or had been pregnant in the past 12 months, 9.3% among postpartum women, and 8.1% among non-pregnant women [15]. It should be noted that the prevalence for postpartum women could include time in which they were pregnant, as this period covers the previous 12 months. Depression during pregnancy and postpartum has specific adverse short- and long-term effects on maternal health during pregnancy (e.g., lower rates of self-care), health outcomes for infants (e.g., preterm delivery), child health and development (e.g., social engagement), and the overall health of families (e.g., marital satisfaction) [16–21].

Almost half of Canadians with depression had never reported depression to a primary care provider [22]; for depression in pregnancy and postpartum, the proportion has been reported to be even higher. For example, the 2005–2012 National Health and Nutrition Examination Survey (NHANES) reported that 88% of depressed pregnant women did not seek mental health care in the past 12 months [23]. The intent of a screening program for depression is to identify symptomatic diseases that would not otherwise be identified or reported (e.g., by spontaneous patient self-report or careful clinical assessment) and to provide early intervention to reduce morbidity and mortality. Current approaches for depression screening are based on the use of questionnaires (e.g., Patient Health Questionnaire [PHQ-9], Beck Depression Inventory for depression generally; Edinburgh Postnatal Depression Scale [EPDS] for depression in pregnancy or postpartum), which have cut-off scores that imply need for further evaluation. If effective, screening for depression would be expected to improve future health through identification and intervention in those who otherwise would not have been identified [24].

### Objective

Our objective was to review the evidence of screening for depression among the general adult population and in pregnant and postpartum women regarding benefits and harms of screening for depression in primary care and non-mental health clinic settings. We conducted a separate systematic review (SR) on each population and the protocols addressing these populations were previously published [25] (PROSPERO # ﻿CRD42018099689, CRD42018099690﻿). It is intended that the results will inform the Task Force in the updating of their guideline recommendation on depression screening for the general adult population, and to develop recommendations for screening for depression in individuals during pregnancy and up to 1 year postpartum in primary health care settings or other non-mental health clinic settings (e.g., obstetrics and gynecology).

**Fig. 1:**
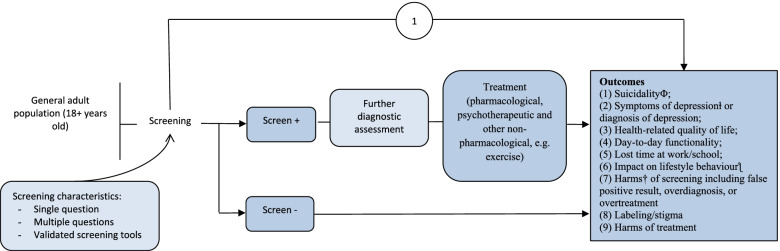
General adult population analytic framework

**Fig. 2:**
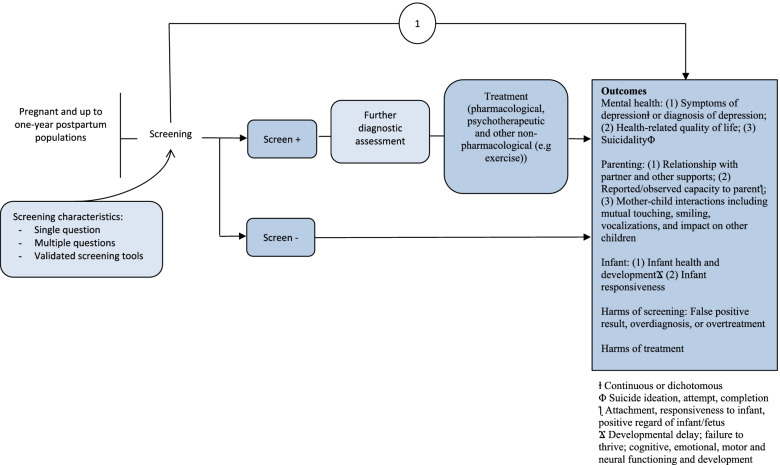
Pregnancy and postpartum analytic framework

The analytic framework depicts the structure used to address the key questions for evaluating the benefits and harms of depression screening (Figs. 1 and 2). We used the following key questions to guide the two SRs (Table 1).

**Table 1:** Key questions to inform recommendations by the Task Force on depression screening in adults and pregnant and postpartum women

General adult population	Pregnant and postpartum population
**KQ1**: What are the benefits and harms of screening versus no screening for depression in the general adult population in primary care or other non-mental health clinic settings?	**KQ1**: What are the benefits and harms of screening versus no screening for depression during pregnancy and up to 1 year postpartum in primary care or other non-mental health clinic settings?
**KQ1a**: What are the benefits and harms of screening versus no screening for depression in the general adult population in primary care or other non-mental health clinic settings for patients targeted because they have characteristics that may suggest elevated risk of depression^a^?	**KQ1a**: What are the benefits and harms of screening versus no screening for depression during pregnancy and up to 1 year postpartum in primary care or other non-mental health clinic settings for patients targeted because they have characteristics that may suggest elevated risk of depression^a^?

^a^Characteristics as defined in primary studies (e.g., trauma early in life, a family history of depression), other than those specified in the exclusion criteria

## Methods

**Fig. 3:**
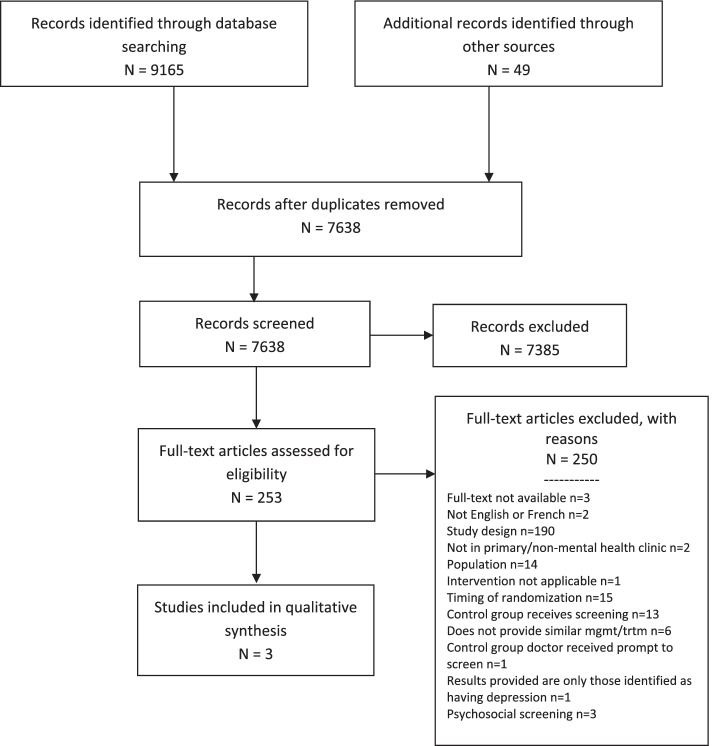
General adult population PRISMA flow diagram

**Fig. 4:**
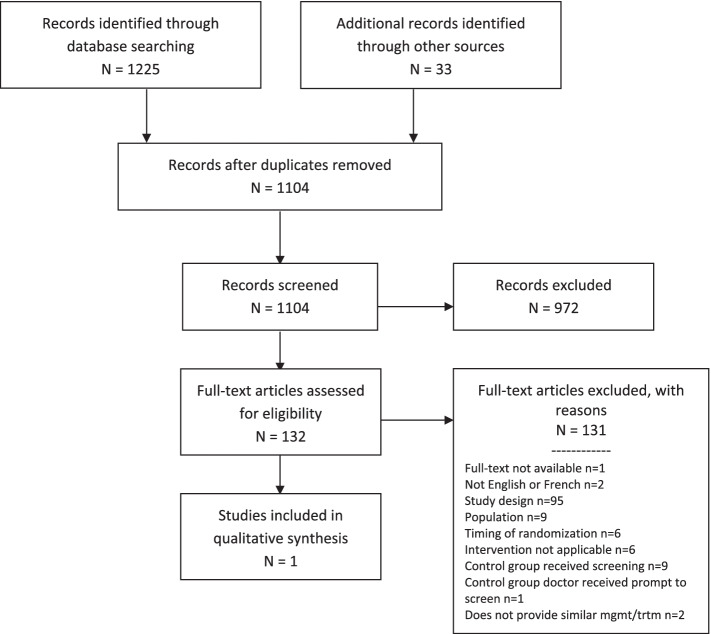
Pregnancy and postpartum PRISMA flow diagram

This review was developed, conducted, and reported according to the Preferred Reporting Items for Systematic Reviews and Meta-Analyses (PRISMA) 2020 statement [26] (Additional file 1). Separate searches were developed and peer-reviewed using the Peer Review of Electronic Search Strategies (PRESS) 2015 guideline [27] (Additional file﻿ 1), for the general adult population and the pregnant and postpartum population. The PRISMA flow diagrams summarizing the process of study selection are presented in Figs. 3 and 4. For additional quality control, we used A Measurement Tool to Assess systematic Reviews (AMSTAR 2) to guide conduct of these reviews [21]. Details on how eligibility criteria and outcomes were determined can be found in the published protocol [25] and PROSPERO registration [CRD42018099690﻿ (adult) and CRD42018099689﻿ (pregnancy and postpartum)].

A Depression Working Group of Task Force members was formed with support from the Public Health Agency of Canada (PHAC) scientific staff and the Ottawa Evidence Review Synthesis Centre (ERSC) on the development of the topic, refinement of the key questions and scope, and rating of outcomes considered most important for creating a recommendation; this working group also sought input from external clinical experts. Patients were also invited to participate in focus group discussions regarding the prioritization of the outcomes. The general adult population focus group included a total of 16 adults (three males, 13 females), aged 22 to 63 (mean 36.5 years, SD 12.21). A total of 15 women (six pregnant and nine postpartum) participated as part of the pregnancy and postpartum group, with five of these women reporting that they were previously diagnosed or treated for depression by a health professional, and two women reported that they were currently receiving treatment for depression. Additional details around outcome selection, rating, and patient engagement can be found in the protocol [25].

### Amendments to the protocol

The review on the general adult population and the eligibility criteria for timing of publication date was changed from May 2012 (the last search date from the previous Task Force review) to inception. This review was an update of a previous Task Force systematic review [2], and for quality assurance, we re-screened the excluded studies at full-text screening from the previous review to determine if any excluded studies met the updated review eligibility criteria.

### Eligibility criteria

The following three criteria were used to identify potentially eligible randomized controlled trials (RCTs) of depression screening [28]: (i) the patient population must be clearly defined and participants must be randomized prior to administering the screening test; (ii) studies with patients who are known to have a current episode of depression or are already being treated for depression close to the time of eligibility assessment are excluded, as screening is intended to identify undetected cases and those who are known to have depression would not be screened in clinical practice. We allowed inclusion if no more than 20% of the study population were known cases, and (iii) similar depression management and treatment resources must be provided to patients in the screening arm of the trial and patients in the non-screening arm of the trial who are identified as depressed via other methods (e.g., unaided clinician diagnosis, patient report). If this last criterion was not followed, it would not be possible to disentangle evidence of the effectiveness of a screening program from evidence of the effectiveness of providing additional treatment and management resources. Additional inclusion and exclusion criteria for both populations are listed in Table 2.

**Table 2:** Eligibility criteria for inclusion and exclusion of studies

	Inclusion criteria		Exclusion criteria
	General adult population (A)	Pregnancy and postpartum (B)	
**Population**	Key question 1: Patients who are 18 years of age and older Key question 1a: Patients who are 18 years of age and older selected for screening because they have characteristics that may suggest elevated risk of depression *Characteristics as defined in primary studies (e.g., trauma early in life, a family history of depression)*	Key question 1: Patients during pregnancy and up to 1 year postpartum of any age Key question 1a: Patients during pregnancy and up to 1 year postpartum selected for screening because they have characteristics that may suggest elevated risk of depression *Characteristics as defined in primary studies (e.g., trauma early in life, a family history of depression)*	• If >20% of study sample have a recent history of depression, have a current diagnosis, or are receiving treatment for depression or other mental disorders (unless results are provided separately from the population of interest) (A and B) • Women with any history of depression during pregnancy or the postpartum period (B)^b^ • Those seeking services due to symptoms of mental disorders (A and B) • Those receiving assessment or care in psychiatric or mental health settings (A and B)
**Intervention**	Interventions that use a single question, small sets of questions, or a screening questionnaire (validated or non-validated) with a pre-defined cut-off score to identify patients who may have depression, but who have not reported their symptoms to healthcare providers or who have otherwise not been identified as possibly depressed by healthcare providers		Interventions that, in addition to screening, include depression care referral or treatment options that are not available to patients identified as depressed in the no screening trial arm
**Comparator**	No depression screening Patients in comparator trial arms may be administered depression symptom questionnaires for the purpose of baseline or outcome assessments as long as scores are not provided to the patients or healthcare providers		
**Outcomes**	• Symptoms of depression (continuous or dichotomous) or diagnosis of MDD (using a validated diagnostic interview) • Health-related quality of life^a^ • Day-to-day functionality • Lost time at work/school • Impact on lifestyle behavior (alcohol abuse, smoking, drugs, gambling, etc.) • Suicidality (suicide ideation, attempt or completion) • False-positive result (positive screen in absence of depressive disorder), overdiagnosis, or overtreatment • Labeling/stigma • Harms of treatment	*Mental health outcomes* • Symptoms of depression (continuous or dichotomous) or diagnosis of MDD (using a validated diagnostic interview) • Health-related quality of life^a^ • Suicidality (suicide ideation, attempt, or completion) • False-positive screens (positive screens in absence of depressive disorder), overdiagnosis, or overtreatment • Labeling/stigma • Harms of treatment *Parenting outcomes* • Relationship with partner and other supports • Reported/observed capacity to parent (attachment, responsiveness to infant, positive regard of infant/fetus) • Mother-child interactions including mutual touching, smiling, vocalizations, and impact on other children *Infant outcomes* • Infant health and development (i.e., developmental delay; failure to thrive) cognitive, emotional, motor, and neural functioning and development • Infant responsiveness	
**Timing**	From literature inception	From literature inception	
**Study design**	Randomized controlled trials (RCTs)* including cluster-controlled trials *Trials of screening in which patient eligibility is determined and then patients are enrolled prior to randomization (i.e., to screening or to no screening). Similar depression management and treatment resources are provided to patients in the screening arm of the trial who were identified as depressed as well as patients in either the screening or the non-screening arms of the trial who were identified as depressed via other methods (e.g., unaided clinician diagnosis, patient report) [28]		RCTs where patient eligibility is determined, and patients are enrolled after randomization. Non-RCTs, controlled before-after studies, interrupted time series, cohort studies, case-control studies, cross-sectional studies, case series, case reports, abstracts, and other publication types (editorials, commentaries, notes, letter, opinions)
**Setting**	Primary care or other non-mental health clinic settings, including specialty clinics such as rheumatology, obstetrics, and gynecology	Primary care or other non-mental health clinic settings, including specialty clinics such as obstetrical, maternal-fetal medicine, and pediatric clinics	Studies conducted in mental health or psychiatric settings
**Language**	English and French		

*MDD* major depressive disorder, *RCT* randomized controlled trial

^a^Validated tools

^b^Women with a history of depression in these periods should be assessed clinically and not just be part of a screening pool

### Data sources and search for studies

Search strategies were developed through an iterative process by an experienced medical information specialist in consultation with the review team. Using the multi-database searching option and deduping tool on the OVID platform, we searched Ovid MEDLINE® ALL, including Epub Ahead of Print, In-Process & Other Non-Indexed Citations, Embase Classic + Embase, and PsycINFO. We also searched CINAHL on EBSCO and the Cochrane Library (Cochrane Reviews, Cochrane Protocols, and CENTRAL) on Wiley. All searches were conducted on October 4, 2018, and updated on May 11, 2020. When possible, animal-only and opinion pieces were removed from the results. No language restriction was applied to the searches. A RCT filter based on the Cochrane Highly Sensitive Search Strategy, sensitivity- and precision-maximizing version (2008 revision), was utilized in all databases except CENTRAL. Due to the large volume of abstracts and potential discrepancies between conference abstracts and final reports, conference abstracts were removed from the search results in Embase and CENTRAL, a feature only available in these two databases. Vocabulary and syntax were adjusted across databases.

As the review for the general adult population was an update from a previous review [2] that did not identify any eligible RCTs, for quality assurance, we reviewed the previous review and the list of excluded studies and screened for eligibility. The search was conducted from 2012 onwards to overlap with that review. Strategies used a combination of controlled vocabulary (e.g., “Depressive Disorder”, “Mass Screening”, “Adult”) and keywords (e.g., “depression”, “screening”, “adults”) (Additional file﻿ 1). As the pregnant and postpartum population was not specifically addressed in the previous review, there was no date restriction for this search. Strategies used a combination of controlled vocabulary (e.g., “Depressive Disorder”, “Mass Screening”, “Pregnancy Complications”) and keywords (e.g., “depression”, “screening”, “pregnancy”) (Additional file 1).

We searched gray literature sources for unpublished documents (e.g., reports, theses, governmental publications) following the Canadian Agency for Drugs and Technologies in Health (CADTH) Grey Matters checklist [29]. We searched the websites of the following organizations between May 11 and May 22, 2020: College of Family Physicians Canada, American College of Physicians, American Academy of Family Physicians, Canadian Nurses Association, American Nurses Association, Canadian Psychiatric Association, Centre for Addiction and Mental Health, Anxiety and Depression Association of America, American Psychological Association, Society of Obstetricians and Gynaecologists of Canada, American College of Obstetricians and Gynecologists, Royal College of Obstetricians and Gynaecologists, Royal Australian and New Zealand College of Obstetricians and Gynaecologists, and the Canadian Association of Midwives. We also searched the following clinical trials registries for ongoing or completed studies on May 15, 2020: ClinicalTrials.gov, International Standard Randomised Controlled Trial Number Registry, Clinical Trials Registry India, CenterWatch, Canadian Cancer Trials, UK Clinical Research Collaboration, and the International Clinical Trials Registry Platform.

Clinical experts were contacted and invited to submit research reports for consideration. For relevant evidence-based clinical practice guidelines and systematic reviews that were found, the reference lists were reviewed, and any potentially relevant study was located and screened for eligibility using the full-text report. Using Robinson et al. as guidance [30], a systematic review needed to meet the following criteria to be considered a potential source for reviewing the reference list: (i) at least one database was searched; (ii) selection criteria were reported; (iii) quality appraisal of included studies was reported; and (iv) a list and synthesis of included studies was provided.

### Study selection

Duplicates across searches were identified and removed using Reference Manager (Thomson Reuters) and EndNote X9.3.3 (Clarivate Analytics) [31, 32]. Screening was completed in two stages using DistillerSR (Evidence Partners) [33]. The first stage was a broad screening of the titles and abstracts. A pilot test of the screening form was performed by four reviewers on a random set of 100 records. Conflicting answers were discussed among the four reviewers and resolved to increase the consistency of screening. The remaining records were then screened independently by two reviewers using the liberal accelerated method, in which only one reviewer was required to include records in order to move them on to a full-text review and a second reviewer was used to verify records to be excluded [34]. As records were screened in random order, each reviewer would not necessarily know if the reference had already been considered irrelevant by the other reviewer. Any conflicting answers were passed through to full-text review.

In stage 2, those records deemed potentially relevant based on title and abstract were subjected to a more focused screening of the full-text reports against the study design, population, intervention, comparison of interest, and the three screening trial criteria. A pilot exercise of five reports was performed by three reviewers. Conflicts in the pilot phase were discussed with the three reviewers, and once agreement was reached, the remaining records were screened independently and in duplicate by two reviewers. Once screening was underway, conflicts were resolved by consensus or a third team member. All reviewers involved in piloting were those involved in the remaining screening. Only English and French articles were evaluated at the full-text stage; records for articles in all other languages were excluded and labeled as “other language.” Articles that were not available electronically were ordered via interlibrary loan, and for timing feasibility, those that were not received within 30 days were excluded with the reason for exclusion labeled as “full text not available.” Bibliographic abstracts for articles not located in the search were excluded and labeled as “abstract.” For full-text screening, where study eligibility was unclear, authors were contacted by email twice, 2 weeks apart, for additional information. If no response was received, the article was excluded and labeled as “unclear” under that question. Clinical expert co-authors and with the guideline working group were contacted for advice in situations where the study reporting was clear, but clinical expertise was needed to understand the clinical context.

### Data abstraction and risk of bias assessment

Standardized data extraction forms were developed a priori and included author and year of publication, funding source, participant information, study design, location, details of the intervention and control group, and the outcomes. No piloting was performed as there were only three included studies. Full data abstraction was completed by one reviewer and verified by a second reviewer, who checked the accuracy of extracted information and any omissions in extraction. Disagreements were resolved by consensus.

We used the Cochrane risk of bias (RoB) tool to assess the RoB of the included trials [35]. No piloting was performed as there were only three included studies. RoB assessment was performed by one reviewer with verification completed by a second reviewer. Disagreements were resolved by consensus. The three domains in the Cochrane RoB that are outcome-specific (i.e., blinding of participants and personnel, blinding of outcome assessors, and incomplete outcome data) were assessed at the outcome level. The overall RoB for the body of evidence involved a judgment of the relative importance of domains, guided by known empirical evidence of bias, the likely direction of bias, and the likely magnitude of bias [35]. We followed the Grading of Recommendations Assessment, Development and Evaluation (GRADE) guidance for determining the extent of the RoB for the body of evidence [36]. For outcome and analysis reporting bias(es), we used the methods outlined in the Agency for Healthcare Research and Quality guidance to determine RoB for that domain [37].

### Data synthesis and statistical analysis

Study characteristics, including country of conduct, primary author, date of publication, number of included participants in each group, details of intervention and comparator, and funding, are summarized narratively and presented in Additional file 1. Relative and absolute effects with 95% confidence intervals (CI) were calculated to facilitate presentation of outcome data according to the GRADE evidence profiles and summary of findings tables. Risk ratios were used to report effects for binary data and confidence intervals were used to calculate the standard deviation of the mean based on the formula provided in the Cochrane Handbook [38] (Section 7.7.3.3) that allows input into Cochrane Review Manager software version 5.3 [39]. Results for all reported outcomes are presented as mean and standardized mean differences, to facilitate ease of access of information for clinicians. The Cochrane Review Manager was used to calculate standardized mean differences (SMDs) and related 95% CIs. GRADE guidance was used for presenting continuous data [40]. Where possible, the number needed to screen was calculated. Although some outcomes differed between the two populations, the same process for synthesis and analysis was used for both the adult and pregnancy/postpartum populations.

#### Additional analysis

We planned to do meta-analyses, subgroup analyses, sensitivity analyses, and test for publication bias if data permitted. However, due to the heterogeneity among the three studies for the general adult population, inclusive of the only included study for the pregnancy and postpartum population, we did not perform any planned analyses and summarized the results narratively.

#### Grading the certainty of evidence and interpretation

We assessed the quality of evidence for individual comparisons and outcomes using the GRADE approach. GRADE tables were prepared for each of the critical and important outcomes using the GRADE framework to assess each domain (i.e., study limitations, imprecision, inconsistency, indirectness, and other considerations [e.g., publication bias]) [41, 42]. This was performed by one reviewer with verification completed by a second reviewer. Disagreements were resolved by consensus. In consultation with available co-authors and guideline working group experts, we were unable to define consensus thresholds for minimum clinically important differences (MCID). Additionally, a search for empirical evidence was performed in PubMed, and there was no consensus to support a threshold.

The GRADE Handbook was used to help determine imprecision. Despite the inability to define specific thresholds for data interpretation, it was judged as unlikely that absolute differences between groups were large enough to be clinically important in almost all cases; for the measurement of depression using the EPDS, uncertainty resided in meaningfulness of applying a cut-off score of 10 or more without additional clinical or diagnostic assessment. Therefore, due to uncertainty and lack of empirical evidence around thresholds, we elected not to calculate an optimal information size (OIS). In the absence of this, we used the GRADE suggestion to inform the rating (i.e., a minimum of 400 events for dichotomous outcomes or 800 participants for continuous outcomes) [43–45]. However, since GRADE also states that this threshold is arbitrary, some judgment around the results was used, as tracked in the GRADE table footnotes.

## Results

### General adult population

#### Search results

The bibliographic search strategies yielded 9165 records. An additional 49 records were found through scanning systematic review bibliographies, full-text publication of protocols from published protocols and clinical registries, and gray literature searching. After de-duplication, 7638 records remained and were screened based on the title and abstract. A total of 253 records were assessed at full text, with three studies meeting the inclusion criteria (Fig. 3) [46–48]. One of these studies [48] was identified and included as a result of the re-examination of the excluded studies from the previous review that we updated for this project and suggested by a clinical expert as a missing study that had been included in other systematic reviews and guidelines [2]. This previously excluded study had fulfilled the previous review eligibility criteria and should have been included in the previous review based on the inclusion and exclusion criteria. This study was also included in a related pregnancy and postpartum review. Additional file 1 provides the bibliographic listing of those excluded during full-text assessment, sorted by reason. A list of ongoing trials is provided in Additional file 1.

#### Study characteristics

Additional file 1 provides study characteristics of the three included studies that assessed screening for depression in the general adult population. Included studies were RCTs conducted in a single health center in the USA [46], general practices in the UK [47], and in Maternal and Child Health (MCH) Centers in Hong Kong, China [48]. The respective populations included participants aged 21 years or older with documented acute coronary syndrome within 2 to 12 months of enrollment [46], participants 45 years or older who consulted for osteoarthritis symptoms in primary care [47], and mothers with 2-month-old babies visiting MCH Centers [48]. The UK study differed from the other two in that it was a pragmatic cluster randomized trial where the general clinical practices were the units of randomization [47]. All studies excluded participants who either had a prior history of depression, who were receiving treatment for depression, or who were participating in other screening programs. Screening interventions and comparators differed between the three studies.

In the US trial, Kronish et al. [46] evaluated systematic screening for depression using the 8-item Patient Health Questionnaire (PHQ-8) compared with usual care; however, baseline data for the screening arm was not recorded. In one intervention arm, 501 participants were screened with notification of primary care clinicians for those with a positive screening result (screen and notify group). In another arm, 499 participants were screened with a primary care clinician notified of any clinically significant depressive symptoms (PHQ-8 score ≥10) and provision of care followed for those with a positive screening result (screen, notify, and treat group); however, this group was not included in the synthesis of this review because the treatment intervention met the predefined exclusion criteria. In the control arm, 500 participants received usual care from their treating clinician and were able to seek mental health screening and/or depression treatment at their own expense (no screening group). The study’s primary outcome of interest was change in quality-adjusted life-years (scores derived from 12-Item Short Form Health Survey (version 2) [SF-12] responses), while the secondary outcome was depression-free days (based on the 10-Item Center for Epidemiologic Studies Depression Scale [CESD-10]). Other reported outcomes included depressive symptoms (measured by the CESD-10 and PHQ-8), harms of depression screening (i.e., loss in appetite, sleep problems, gastrointestinal upset, and bleeding), and mortality (not an outcome of interest for this review). Outcomes were measured at 6, 12, and 18 months.

In the UK trial, Mallen et al. [47] evaluated an electronic template to prompt routine screening for anxiety and depression compared with usual care. In the intervention arm, the electronic template prompted the general practitioner (GP) to ask two questions about depression (Patient Health Questionnaire-2 [PHQ-2]), two questions about anxiety (Generalized Anxiety Disorder-2 [GAD-2]), and one question about pain intensity. With the two PHQ-2 items, the authors utilized a dichotomous yes/no response rather than the standard PHQ-2 scoring. A positive response to either question was deemed a positive screen. In the control arm, the electronic template prompted the GP only to ask the question about pain intensity. In both arms, no additional treatment resources or services for depression, anxiety, or pain management were provided as part of the study. A total of 2042 respondents consented to further contact and were sent post-consultation questionnaires, of whom 1412 returned the questionnaire and were included in the analysis. Study authors reported that participants had broadly similar characteristics at baseline. Participants returned the post-consultation questionnaire on average 24 days after initial consultation in the intervention group (range 9–149 days) and 22 days in the control group (range 3–106 days). In addition, they were sent questionnaires to determine outcomes at 3, 6, and 12 months after the initial appointment with the GP. The authors reported the total number of questionnaires contributing to each outcome, but not specifically for each time point. As the primary outcome of the trial was pain intensity, there is no information provided about post-screening treatment for depression. All adjusted effect estimates and 95% CIs were reported. Analyses were adjusted using general practice and repeated measures as cluster-level random effects and fixed-effect covariates at practice level and patient level (age, sex, and time between consultation and post-consultation response) (Additional file 1). The standardized mean difference (SMD) has been calculated, but it is based on the raw results and does not account for clustering as there was insufficient information to be able to calculate adjusted SMDs.

In the Hong Kong trial, Leung et al. [48] evaluated screening for postnatal depression. Two hundred thirty-one participants in the intervention group were screened for postnatal depression using the Edinburgh Postnatal Depression Scale (EPDS), while 231 participants in the control group received usual care by clinical assessment. The EPDS consisted of 10 questions with scores ranging from 0 to 30, and participants with score above the cut-off (9/10) or suicidal ideation (positive answer to question 10) were offered non-directive counseling by Maternal and Child Health nurses or management by the community psychiatric team as appropriate. The Chinese version of the EPDS was validated with Hong Kong women at 6 weeks postnatal, against the structured clinical interview for DSM-III-R. The outcome of interest was maternal mental health as measured by depression scores calculated from the EPDS measured at 2, 6, and 18 months postpartum.

#### Outcomes

Results for all three studies are presented in Additional file 1. Ratings of risk of bias by study are included in Additional file 1.

The GRADE Evidence Profiles and Summary of Findings Tables including explanations for all rating are available in Additional file 1. We did not pool results for any of the outcomes due to substantial differences between study populations, approaches to screening for depression, time points, and high risk of bias.

#### Benefits of screening

##### Symptoms of depression

Kronish et al. [46] measured symptoms of depression using the CESD-10 and PHQ-8, change in depressive symptoms using CESD-10 scores, and depression-free days converted from CESD-10 scores. We rated the certainty in the evidence as moderate for these outcomes, except for change in depressive symptoms and depression-free days among women, owing to serious concerns of indirectness as the study only included adults who were recently documented with acute coronary syndrome which is not representative of the wider general adult population. Regarding change in depressive symptoms and depression-free days among women, we rated the certainty in the evidence as low due to serious concerns of indirectness as described above and serious concerns of imprecision as the sample size was low (see Additional file 1).

##### Depression score (CESD-10)

Screening for depression likely results in little to no difference in symptoms of depression at any time point (i.e., baseline, 6, 12, and 18 months) (SMD of 0.06 lower [from 0.18 lower to 0.07 higher] at 18 months)—moderate certainty: serious indirectness. Similarly, screening likely results in little to no difference on the changes in depressive symptoms among men (SMD 0.09 lower [0.24 lower to 0.06 higher]) and women (SMD 0.09 lower [0.32 lower to 0.15 higher])—moderate certainty: serious indirectness and low certainty: serious indirectness, serious imprecision.

##### Depression score (PHQ-8)

At 18 months, screening for depression likely results in little to no difference in symptoms of depression (SMD of 0.02 lower [from 0.15 lower to 0.10 higher])—moderate certainty: serious indirectness. Screening also likely results in little to no difference in symptoms of depression among men (SMD 0.07 lower [0.22 lower to 0.07 higher]) and women (SMD 0.02 higher [0.21 lower to 0.26 higher]) —moderate certainty: serious indirectness and low certainty: serious indirectness, serious imprecision. Note, baseline data for the screening arm was not measured by study authors.

##### Depression-free days (CESD-10 score converted to depression day)

Screening for depression likely results in little to no difference in depression-free days (SMD of 0.07 higher [from 0.05 lower to 0.19 higher] at 18 months)—moderate certainty of the evidence: serious indirectness. Similarly, screening likely results in little to no difference in depression-free days among men (SMD 0.08 higher [0.07 lower to 0.23 higher]) and women (SMD 0.06 higher [0.17 lower to 0.30 higher])—moderate certainty: serious indirectness and low certainty: serious indirectness, serious imprecision.

Mallen et al. [47] measured symptoms of depression using the PHQ-8. We rated the certainty in the evidence as very low owing to very serious concerns with the risk of bias because a large proportion of participants were lost to follow-up from those who were screened and indirectness as the study included adults seeking consultation for osteoarthritis and receiving screening for anxiety (see Additional file 1).

##### Depression score (PHQ-8)

The evidence is very uncertain about the effect of screening for depression on symptoms of depression at any time point (i.e., post-consultation, 3, 6, and 12 months post-consultation) (SMD 0.10 higher [0.03 lower to 0.23 higher] at 12 months)—very low certainty: very serious RoB, very serious indirectness.

Leung et al. [48] measured symptoms of depression with the EPDS and GHQ-12. We rated the certainty in the evidence as very low for these outcomes owing to very serious concerns of risk of bias (lack of blinding and selective outcome reporting), serious concerns of indirectness (limited to postpartum women), and serious concerns of imprecision (small sample size) (see Additional file 1). These concerns are discussed and further explained in the “Discussion” section.

##### Number identified as depressed among women (EPDS score)

The evidence is very uncertain about the effects of screening for symptoms of depression using EPDS in postpartum women—very low certainty: very serious RoB, serious indirectness, serious imprecision.

At baseline (2 months postpartum), 73 (36.1%) women in the screening arm and 14 (6.0%) women in the no screening arm were assessed as having probable postpartum depression; 58 women in the screening arm scored ≥10 on the EPDS and nine women scored <10 on the EPDS but had a positive response on the suicidal ideation question. Among those with an EPDS score <10 and without suicidal ideation, six were clinically assessed as having probable postpartum depression. All participants were offered treatment; however, 18 (8.0%) in the screening arm (10 defaulted the recommended treatment, eight were inadvertently discharged) and three (1.0%) in the no screening arm (inadvertently discharged) did not receive treatment. At 6 months postpartum (i.e., 4 months after randomization), it was reported that women in the screening arm had a 41% reduced risk of depression with EPDS relative to those in the no screening arm (RR 0.59, 95% CI 0.39 to 0.89); this corresponds to 11 (95% CI 6 to 50) needed to screen to prevent one case of postpartum depression at 6 months postpartum. However, after adjustment for the positive predictive value (44%) of the Chinese EPDS for depression ascertained by clinical interview in the Hong Kong population, the number needed to screen increased to 25 (95% CI 14 to 114) [48]. After adjusting for known predictors of postpartum depression using multiple logistic regression (marital relationship at 2 months, history of psychiatric illness, depression during pregnancy, and relationship with mother-in-law), Leung et al. stated that the effect remained statistically significant, but did not report or provide the adjusted RR. Leung et al. also reported results at 18 months postpartum, but the no screening arm was already screened with the EPDS at 6 months postpartum and offered treatment or follow-up services for those who scored ≥10, thereby removing the screened versus not screened comparison. Furthermore, there is uncertainty in using a cut-off score of ≥10 on the EPDS with no further clinical/diagnostic assessment [49]. Since we were unable to otherwise define a threshold for a clinically important difference, it is not clear whether an important difference is being observed in Additional file 1.

##### Depression score (EPDS)

The evidence is very uncertain about the effect of screening for depression in mean EPDS scores at 6 months postpartum (the mean EPDS score was 1.36 points lower in the screening group [95% CI −0.63 to −2.09; SMD 0.34, 95% CI −0.15 to −0.52]); very low certainty: very serious RoB, serious imprecision and serious indirectness.

##### Depression score (GHQ-12)

The evidence is very uncertain about the effect of screening for depression on the mean GHQ score at 6 months postpartum (SMD −0.16, 95% CI −0.35 to 0.02); very low certainty: very serious RoB, serious indirectness, serious imprecision.

##### Health-related quality of life

Kronish et al. [46] measured quality-adjusted life-years (QALYs) and quality-of-life utility scores.

We rated the certainty in the evidence as moderate for these outcomes, except for change in QALYs among women, owing to serious concerns of indirectness as the study included adults who were recently documented with acute coronary syndrome. Regarding change in QALYs among women, we rated the certainty in the evidence as low due to serious concerns of indirectness as described above and serious concerns of imprecision as the sample size was low (see Additional file 1).

##### Change in mean QALYs

Screening for depression likely results in little to no difference on the change in mean QALYs from baseline to 18 months (SMD of 0 [from 0.12 lower to 0.12 higher])—moderate certainty: serious indirectness. Screening likely results in little to no difference in depression-free days among men (SMD 0.05 higher [0.09 lower to 0.20 higher]) and women (SMD 0.22 lower [0.45 lower to 0.02 higher])—moderate certainty: serious indirectness and low certainty: serious indirectness, serious imprecision.

##### Change in quality-of-life utility scores

Screening for depression likely results in little to no difference on the change in quality-of-life utility scores at any time point (i.e., baseline, 6, 12, and 18 months) (SMD 0.04 lower [0.17 lower to 0.08 higher] at 18 months)—moderate certainty: serious indirectness.

Mallen et al. [47] measured the quality of life using the Medical Outcomes Study Short Form 12 Mental Component (SF-MCS) and Physical Component (SF-PCS) scores. We rated the certainty in the evidence as very low owing to very serious concerns with the risk of bias because a large proportion of participants were lost to follow-up from those who were screened and indirectness as the study included adults seeking consultation for osteoarthritis and receiving screening for anxiety (see Additional file 1).

##### SF-MCS scores

The evidence is very uncertain about the effect of screening for depression on the mental quality of life at any time point (i.e., post-consultation, 3, 6, and 12 months post-consultation) (SMD 0.04 lower [0.16 lower to 0.09 higher] at 12 months)—very low certainty: very serious RoB, very serious indirectness.

##### SF-PCS scores

The evidence is very uncertain about the effect of screening for depression on the physical health quality of life (adjusted MD −0.66, 95% CI −2.25 to 0.93; SMD 0.08 lower [0.21 lower to 0.04 higher] at 12 months) at post-consultation, 3, and 12 months post-consultation—very low certainty: very serious RoB, very serious indirectness. At 6 months post-consultation, screening for depression may decrease physical health quality of life (adjusted MD −1.77, 95% CI −3.22 to −0.32; *p* = 0.017; SMD −0.26, 95% CI −0.13 to −0.38), but the evidence is very uncertain—very low certainty: very serious RoB, very serious indirectness.

#### Harms of screening

Kronish et al. [46] reported harms attributable to antidepressant medications (i.e., any bleeding, changes in appetite, drowsiness, and gastrointestinal upset) among the screened and no screened group.

##### Bleeding

Screening for depression likely results in little to no difference in bleeding at any time point (i.e., 6, 12, and 18 months) (RR 1.00 [0.69 to 1.44] at 18 months); this corresponds to 0 fewer per 1000 patients (36 fewer to 52 more per 1000 patients)—moderate certainty: serious indirectness.

##### Changes in appetite

Screening for depression likely results in little to no difference in increased appetite at any time point (i.e., 6, 12, and 18 months) (RR 1.00 [0.75 to 1.34] at 18 months); this corresponds to 0 fewer per 1000 (from 44 fewer to 61 more)—moderate certainty: serious indirectness. Screening for depression may result in a slight reduction in decreased appetite (RR 0.85 [0.63 to 1.15] at 18 months); this corresponds to 27 fewer per 1000 (from 66 fewer to 27 more)—moderate certainty: serious indirectness.

##### Drowsiness

Screening for depression may result in a slight decrease in drowsiness at any time point (i.e., 6, 12, and 18 months) (RR 0.94 [0.81 to 1.09] at 18 months); this corresponds to 28 fewer per 1000 (from 88 fewer to 42 more)—moderate certainty: serious indirectness.

##### Gastrointestinal upset

Screening for depression may result in a slight decrease in gastrointestinal upset (RR 0.88 [0.69 to 1.12] at 18 months); this corresponds to 30 fewer per 1000 (from 78 fewer to 30 more)—moderate certainty: serious indirectness.

Leung et al. [48] had reported adverse events; however, the effect size was not estimable—very low certainty: very serious RoB, serious indirectness, serious imprecision.

#### Outcomes not reported

Many outcomes of interest for this review (as reported in PICOs framework) that were established through consultation with the Task Force, their external clinical experts, and patient partners were not reported in the included studies. Not only is there a paucity of data, but the included studies are not necessarily providing clinically helpful information as they are not examining outcomes that are deemed important to guideline panel experts and patient partners. These include diagnosis of depression using a validated diagnostic interview (e.g., the Structured Clinical Interview for DSM (SCID)) at a follow-up time point, day-to-day functionality, lost time at work/school, impact on lifestyle behavior, suicidality, false positive results, overdiagnosis or overtreatment, and labeling/stigma.

### Pregnancy and postpartum women

#### Search results

The search strategies resulted in 1225 records and an additional 33 records were found through scanning systematic review bibliographies, full-text publication of protocols from published protocols and clinical registries, and gray literature searching. After de-duplication, 1104 records remained and were screened based on the title and abstract. A total of 132 records were assessed at full text, with one RCT by Leung et al. included [48] (Fig. 4). Additional file 1 provides a bibliographic list of the excluded studies on full text assessment, with reasons. A list of ongoing trials is provided in Additional file 1.

#### Study characteristics

Leung et al. [48] performed a randomized controlled trial among 462 women enrolled 2 months postpartum. Women were seen at the Maternal and Child Health Centres in Hong Kong and were randomized to screening with the Edinburgh Postnatal Depression Scale (EPDS) (*n* = 231) or no screening (*n* = 231) in addition to usual practice in which nurses carried out clinical assessment. The EPDS consists of 10 questions and scores may range from 0 to 30, with higher scores identifying those as higher likelihood of depression [50]. All women including the control group received clinical assessment at the 2-month postpartum appointment. Participants in either group identified as potentially depressed (i.e., having a score of ≥10 on EPDS, by answering positive to the suicidal ideation question in the intervention group, or through clinical assessment in either group) were offered non-directive counseling by nurses or management by a community psychiatric team (55/73 in the intervention and 11/14 in the control group). Additional file 1 provides the study characteristics table, results table (binary data), and results table (continuous data), respectively. Groups were reported to be similar at baseline, although there were some differences in some baseline measures (e.g., history of psychiatric illness, separated/divorced/widowed/never married, does not live with child all the time).

#### Outcomes

Results for are presented in Additional file 1. Ratings of risk of bias by study are included in Additional file 1. The GRADE Evidence Profiles and Summary of Findings Tables including explanations for all rating are available in Additional file 1.

#### Benefits of screening

##### Maternal mental health outcomes

We rated the certainty in the evidence as very low for these outcomes owing to very serious concerns of risk of bias (lack of blinding and selective reporting) and serious concerns of imprecision (small sample size) (see Additional file 1) and further explained in the “Discussion” section.

##### Number identified as depressed among women (EPDS score)

The evidence is very uncertain about the effect of screening for depression using the EPDS in postpartum women—very low certainty: very serious RoB and serious imprecision.

At baseline (2 months postpartum), 73 of the 231 women in the screening arm (36.1%) were identified as potentially depressed. Of these, 58 women scored ≥10 on the EPDS, nine women scored <10 on the EPDS, but had a positive response on the suicidal ideation question, and six, among those with EPDS < 10 and without suicidal ideation, were clinically assessed as having probable postpartum depression. In the control group, 14 of the 231 (6%) women were clinically assessed as having probable postpartum depression. Although all women should have been offered treatment, 18 women (8%) in the screening group (10 women defaulted the recommended treatment, eight inadvertently discharged) and three women (1%) in the no screening group (all inadvertently discharged) did not receive treatment. At 6 months postpartum (i.e., 4 months after randomization), women in the screening group had a 41% reduced risk of depression with EPDS relative to those in the unscreened group (RR 0.59, 95% CI 0.39–0.89; Additional file 1). The number needed to screen to prevent one case of postpartum depression at 6 months postpartum was 11 (95% CI 6 to 50), but after adjustment for the positive predictive value (44%) of the Chinese EPDS for depression ascertained by clinical interview in the Hong Kong population, the number needed to screen became 25 (95% CI 14 to 114) [48]. After adjusting for known predictors of postpartum depression (marital relationship at 2 months, history of psychiatric illness, depression during pregnancy, and relationship with mother-in-law) using multiple logistic regression, the authors reported that the effect remained statistically significant (adjusted RR not provided by study authors). The study authors reported results at 18 months postpartum; however, the control group was given the EPDS at 6 months postpartum and was offered treatment/follow-up services for those who scored ≥10, thereby removing the screened versus not screened comparison. There is uncertainty in applying a cut-off score of ≥10 on the EPDS with no further clinical/diagnostic assessment [49]. Since we were unable to otherwise define a threshold for a clinically important difference, it is not clear whether an important difference is being observed in Additional file 1 and the evidence was rated as very low certainty. Therefore, given the available information, the evidence is very uncertain for symptoms of depression measured with EPDS score ≥10 due to very serious concerns with risk of bias and serious concerns with imprecision.

##### Depression score (EPDS)

At 6 months postpartum, the mean EPDS score was 1.36 points lower in the screening group (95% CI −0.63 to −2.09; SMD 0.34, 95% CI −0.15 to −0.52; Additional file 1). The evidence is very uncertain about the effect of screening for depression on the mean EPDS score at 6 months postpartum—very low certainty: very serious RoB and serious imprecision.

##### Depression score (GHQ-12)

The evidence is very uncertain about the effect of screening for depression on the mean score of the General Health Questionnaire (GHQ) at 6 months postpartum (SMD −0.16, 95% CI −0.35 to 0.02) (Additional file 1)—very low certainty: very serious RoB and serious imprecision.

##### Parenting and relationship outcomes

We rated the certainty in the evidence as very low for these outcomes owing to very serious concerns of risk of bias (selective reporting and lack of blinding) and serious concerns of imprecision (small sample size) (see Additional file 1).

##### Parenting stress index-short form (PSI-SF) score

Parenting outcomes were measured with the Parental Stress Index (PSI) tool, which is designed to evaluate the magnitude of stress in the parent-child system.

The evidence is very uncertain about the effect of screening for depression in the total mean score on the PSI (SMD of 0.17 lower [from 0.35 lower to 0.01 higher]) or any of its subscales (Parental Distress, Parent-Child Dysfunctional Interaction, Difficult Child) at 6 months postpartum—very low certainty: very serious RoB and serious imprecision.

##### Marital satisfaction score

Martial satisfaction was measured using the Chinese Kansas Marital Satisfaction Scale. The evidence is very uncertain about the effect of screening for depression in the marital satisfaction score at 6 months postpartum (SMD of 0.15 higher [from 0.03 lower to 0.34 higher])—very low certainty: very serious RoB and serious imprecision.

##### Infant outcomes

We rated the certainty in the evidence as low and very low for these two outcomes owing to serious or very serious concerns of risk of bias (selective reporting and/or lack of blinding, respectively) and serious concerns of imprecision (small sample size) (see Additional file 1).

##### Infant body weight

Screening for depression likely results in little to no difference in the mean infant body weight at 6 months postpartum (SMD 0.06, 95% CI −0.12 to 0.24)—low certainty: serious RoB and serious imprecision.

##### Number of infant hospitalizations

The evidence is very uncertain about the effect of screening for depression in the mean number of infant hospitalizations at 6 months postpartum (SMD 0.06, 95% CI —0.13 to 0.24)—very low certainty: very serious RoB and serious imprecision.

#### Harms of screening

No adverse events were reported in either group during the study duration and it is unclear how this information was sought. We have not assigned a level of importance for this outcome, as there was no information provided on how adverse events were collected/reported. The certainty of the evidence was rated as very low due to very serious concerns with risk of bias and serious concerns with imprecision. Therefore, the evidence is very uncertain for the harms of screening for depression.

#### Outcomes not reported

A key outcome was not reported in the one included study [48], i.e., the diagnosis of depression using a validated diagnostic interview (e.g., the Mini International Neuropsychiatric Interview (MINI)). Other not reported outcomes of interest for this review include health-related quality of life, false-positive screens, overdiagnosis or overtreatment, labeling/stigma, mother-child interactions, infant neurodevelopment, and infant responsiveness. Although suicidality was evaluated with question 10 on the EPDS, results were not presented separately for those who were identified as depressed at follow-up based on this question (as was provided in the baseline results).

## Discussion

For this update, we included three randomized trials that have evaluated the effectiveness of screening for depression. All three trials were included in the general adult population review, while one of the three trials were included in the pregnant and postpartum population review. Across outcomes for the general adult population, screening for depression likely results in little to no effect for screening. There was moderate certainty (serious indirectness) in the evidence from Kronish et al. [46] that screening for depression likely results in little to no difference; however, the evidence was uncertain from Mallen et al. [47] (very serious RoB, very serious indirectness) and Leung et al. [48] (very serious RoB, serious indirectness, serious imprecision).

None of the trials focused on a specific primary care population although the study populations would have been encountered in a primary care setting for non-disease-specific visits (for example, participants 21 years or older with documented acute coronary syndrome, participants 45 years or older who consulted for osteoarthritis symptoms, and mothers with 2-month-old babies). Furthermore, Kronish et al. [46] stated that their results and the applicability to depression screening conducted outside the context of the trial may be different because approximately half of the patients approached declined to enroll in the study. Additionally, none of the trials included patients who had characteristics that may suggest elevated risk of depression (KQ1a), adding no new evidence to the adult SR update in 2013, which did not include any results from trials [2]. Consequently, there is little information to determine the effectiveness of screening in these populations, and what information exists has several limitations.

At the review’s full-text screening, there were 250 studies excluded in the general adult population and 131 studies in the pregnancy/postpartum population. The majority of these were excluded because of their reported study design (adult: 190; pregnancy/postpartum: 95). One of the three criteria to determine a well-designed depression screening trial [28] specifies that similar management and treatment resources need to be made available to all patients identified with depression, both those who received depression screening and those identified through other means in the non-screening group. The importance of this criterion is to be able to isolate the impact of the screening intervention, without a differential influence of subsequent management/treatment on outcomes. Operationalizing this criterion was difficult in the Mallen trial [47]. The authors provided clear documentation of management and treatment resources available to participants in the intervention group. However, these similar resources available to those who were identified as depressed in the comparator group were less clear. In line with the documentation of management and treatment resources recommended in the extension to the CONSORT guideline for pragmatic trials [51], it is stated that no additional treatment resources or services for depression, anxiety, or pain management were provided in either arm. However, in the intervention group, there was specific signposting in the electronic template used at point-of-care to the National Institute for Health and Care Excellence (NICE) guidelines on managing comorbid depression and anxiety [52], together with brief face-to-face training on these guidelines and quick reference versions of the guidelines in all consulting rooms in the intervention practices. While the specific signposting was linked to the screening offered in the intervention arm, arguably the face-to-face training and provision of quick reference versions of the guidelines were a resource that differed between the intervention and control arms. Nevertheless, as psychotherapy training is broad, and the variation of treatment applied could be diverse, the trial was included since the treatment and management resources available to patients in both groups should have been largely the same.

Only one trial in postpartum women was identified [48]. In a commentary, possible concerns about the trial have been identified [49]. These concerns include that no women identified as possibly having depression (either through EPDS or clinical assessment) was further evaluated to determine depression diagnosis and whether treatment was necessary; all participants, in both groups, received a clinical assessment at baseline; a primary outcome in the trial registry record being reported as a secondary outcome in the results; and the effect sizes they reported per woman treated were much larger than other estimates published in treatment SRs for counseling. There is also a large variation in what cut-off scores are being used in the EPDS to determine those who are screened as possibly being depressed [53], as many studies use a cut-off score of 12 or 13 [54]. Given these concerns and the uncertainty of the evidence, the true effects of screening for depression are unknown.

In comparing the results from this review with other SRs of depression screening in the general adult population, the United States Preventive Services Task Force (USPSTF) guideline [4], based on a SR by O’Connor et al. [55], recommended screening for depression, if there were adequate systems in place. These systems would be to ensure accurate diagnosis, effective treatment, and appropriate follow-up. The review included a single trial [56], published in 1999, that compared screening to usual care case-finding. However, the usual care case-finding group included one question asking participants “Have you felt depressed or sad much of the time in the past year?”, which would have been considered screening and was therefore excluded from our review since there was not a screened versus not-screened comparison. The 2014 report by the United Kingdom National Screening Committee (UK NSC) [57] was not based on a systematic review, but used information from the 2010 NICE guideline, 2009 USPSTF guideline, and 2013 Task Force guideline (which reported no RCTs) to help develop their recommendation. The UK NSC states that their policy is that “routine screening of the population or subsets of the population for depression is not recommended.” This report notes that questionnaire-based tests to identify people who are at risk of depression are not sufficiently accurate when used in the general population, and many people would be falsely identified as having depression.

In the pregnant and postpartum population, the aforementioned USPSTF guideline [4] and a SR [5], which included six RCTs, supported a recommendation for screening for depression in this population, if systems and staff are available. These systems would be to ensure accurate diagnosis, effective treatment, and appropriate follow-up. The American College of Obstetrics and Gynecology (ACOG) guideline, based on a SR by Myers et al. [58], included four RCTs and recommended routine depression screening when staff-assisted depression care programs were in place to ensure accurate diagnosis and effective treatment and follow-up. There were three common trials between these reviews [48, 59, 60], and three unique trials included in only one of these guidelines [61–63]. Leung et al. [48] was included in both reviews. The review for the USPSTF guideline did not consider any of the studies to use “a straightforward design that compared usual care plus screening (and no additional treatment components) to usual care without screening.” Among the five trials, all were excluded from this current review. Morell et al. [60], Yawn et al. [59], Zlotnick et al. [63], and MacArthur et al. [61] did not provide similar management and treatment resources to the intervention and control groups. For example, in Yawn et al., the intervention group received education and tools for postpartum depression screening, diagnosis, and initiation of therapy, while the control group received a 30-min presentation about postpartum depression. Lastly, Wickberg [62] was not included as it was a naturalistic comparison and not a randomized controlled trial. The UK NSC postnatal depression screening recommendation, last updated in 2011, recommended against a screening program [3]. This recommendation was based on an evidence summary (i.e., not a systematic review) and states that the use of current identification strategies would result in a significant number of false positives. They emphasized the lack of evidence from trials and found insufficient evidence that screening significantly improved health outcomes for mother or baby.

### Implications for research

More high-quality trials are needed to inform this area of inquiry and to cover the breadth of populations, for generalizability. First, researchers should consider the three aforementioned design criteria when developing their depression screening trial. Second, the Consolidated Standards of Reporting Trials (CONSORT) statement (http://www.consort-statement.org/), which provides a minimum set of recommendations for reporting randomized trials, should be followed. There is direct [64] and indirect [65] evidence that use of CONSORT is associated with improvements in the quality of reporting RCTs. Using CONSORT for reporting the RCTs might have allowed for fewer unclear judgements in the risk of bias assessments. Full details of the intervention and comparator group would allow for clearer interpretation of the aforementioned depression screening trial criteria. Developers of pragmatic trials or cluster trials can also benefit from using the relevant extensions of the CONSORT statement. Third, there is little consensus on what constitutes a minimally clinical important difference for depression screening. Further research in this area would allow for greater interpretation around measures of imprecision. Lastly, outcome switching, the failure to report pre-specified outcomes without justification, was commonly observed among research articles [66, 67] and can present problems in interpreting results. Any deviations from the protocol should be reported and justified [68]. For example, although the GHQ at 6-month outcome was reported in the Leung study [48], it was listed as a primary outcome in the trial registry and as a secondary outcome in the study results. We could not find a justification for this change.

Several outcomes that were of interest in this review were not recorded or reported in the included trials. Therefore, future trials may benefit from the development of a core set of patient-important outcomes, as promoted by the Core Outcomes Measures in Effectiveness Trials (COMET) initiative. No core sets are currently available, but a study is underway by Chevance and colleagues who are developing a core outcome set for major depressive disorder (http://www.comet-initiative.org/studies/details/1105). Although this is specific to therapeutic interventions, they are likely a subset of the outcomes relevant to screening but would not capture outcomes related to diagnostic care nor potential adverse effects of screening. Additionally, there is little to no empirical research evaluating thresholds for defining clinically important differences. This limited our ability to definitively determine if there were important differences in the data presented.

A few trials are currently underway and may be relevant (Additional file 1). Authors of these trials were contacted to see if the trial would meet the eligibility criteria that was used in this review, with three authors responding. One author of a trial in the adult population (ACTRN12606000483550) responded and provided three published articles related to the trial and none would have met the inclusion criteria based on study design [69–71]. Therefore, this trial has not been included in Additional file 1. The last author responded that the trial (NCT00433238) was finished and stated that there is no publication at present.

The strengths of our work lie in the use of an a priori protocol, consideration of criteria for determining well-conducted trials, peer-review evaluation of our search strategies, and updating from a previously published systematic review to reduce duplication of effort and research waste. With input from the guideline working group, clinical experts, and patients, we assembled a group of outcomes of importance to those stakeholders. The risk of missing data by not including languages other than English and French (*n* = 2 adults; *n* = 2 pregnancy/postpartum) is likely low based on the information provided in the abstracts.

## Conclusion

In our systematic evaluation of the literature, there was moderate certainty in the evidence from one trial [46] that screening for depression in the general adult population in primary care or non-mental health clinic settings likely results in little to no difference on reported outcomes; however, the evidence was uncertain from the other two included trials [47, 48]. The evidence is very uncertain about the effect of screening for depression in pregnant or postpartum women in primary care or non-mental health clinic settings. Well-conducted and better-reported trials are needed that meet the screening trial criteria used in this review.

## Supplementary information

**Additional file 1.** Depression additional files.

## Data Availability

Not applicable.

## Abbreviations

CADTH: Canadian Agency for Drugs and Technologies in Health
CI: Confidence interval
DSM: Diagnostic and Statistical Manual of Mental Disorders
EPDS: Edinburgh Postnatal Depression Scale
GHQ: General Health Questionnaire
GP: General practitioner
GRADE: Grading of Recommendations Assessment, Development and Evaluation
ICD-10: International Classification of Diseases, 10th Revision
MD: Mean difference
MDD: Major depressive disorder
MDE: Major depressive episode
NICE: National Institute for Health and Care Excellence
PHQ: Patient Health Questionnaire
PRISMA: Preferred Reporting Items for Systematic Reviews and Meta-Analyses
PROSPERO: International Prospective Registry of Systematic Reviews
PSI: Parental Stress Index
RCT: Randomized controlled trial
RoB: Risk of bias
SF-MCS: Medical Outcomes Study Short Form 12 mental component
SF-PCS: Medical Outcomes Study Short Form 12 physical component
SMD: Standardized mean difference
SR: Systematic review
UK NSC: United Kingdom National Screening Committee
USPSTF: United States Preventive Services Task Force

## References

[1] C. T. F. on P. H. Care (2013). Recommendations on screening for depression in adults. CMAJ.

[2] Keshavarz H (2013). Screening for depression: a systematic review and meta-analysis. CMAJ Open.

[3] The UK NSC recommendation on postnatal depression screening in pregnancy, Current UK NSC recommendations. https://legacyscreening.phe.org.uk/postnataldepression (Accessed 3 Apr 2018).

[4] Siu AL (2016). Screening for depression in adults: US preventive services task force recommendation statement. JAMA.

[5] O’Connor E, Rossom RC, Henninger M, Groom HC, Burda BU (2016). Primary care screening for and treatment of depression in pregnant and postpartum women: evidence report and systematic review for the US preventive services task force. JAMA.

[6] American College of Obstetricians and Gynecologists (2018). Optimizing postpartum care no.736. Obstet Gynecol.

[7] Myers ER, et al. Efficacy and safety of screening for postpartum depression. Rockville; 2013. Accessed: 13 Feb 2018. [Online]. Available: http://www.ncbi.nlm.nih.gov/books/NBK137724/

[8] Public Health Agency of Canada (PHAC), A report on mental illness in Canada (archived). 2002. Accessed: 14 Feb 2020. [Online]. Available: https://mdsc.ca/documents/Publications/Report%20on%20mental%20illness%20in%20canada_EN.pdf

[9] Lam RW (2016). Canadian network for mood and anxiety treatments (CANMAT) 2016 clinical guidelines for the management of adults with major depressive disorder. Can J Psychiatr.

[10] American Psychiatric Association (2013). Diagnostic and statistical manual of mental disorders.

[11] World Health Organization. The ICD-10 classification of mental and behavioural disorders. Diagnostic criteria for research. Geneva; 1992. [Online]. Available: http://www.who.int/classifications/icd/en/GRNBOOK.pdf

[12] “WHO | Depression,” WHO. http://www.who.int/mental_health/management/depression/en/ (Accessed 8 Feb 2018).

[13] Patten SB, Williams JVA, Lavorato DH, Wang JL, McDonald K, Bulloch AGM (2015). Descriptive epidemiology of major depressive disorder in Canada in 2012. Can J Psychiatr.

[14] Lim K-L, Ohinmaa A, Schopflocher D, Dewa C (2008). A new population-based measure of the economic burden of mental illness in Canada. Chronic Dis Canada.

[15] Vesga-López O, Blanco C, Keyes K, Olfson M, Grant BF, Hasin DS (2008). Psychiatric disorders in pregnant and postpartum women in the United States. Arch Gen Psychiatry.

[16] Stuart-Parrigon K, Stuart S (2014). Perinatal depression: an update and overview. Curr Psychiatry Rep.

[17] Stewart DE (2011). Clinical practice. Depression during pregnancy. N Engl J Med.

[18] Dubber S, Reck C, Müller M, Gawlik S (2015). Postpartum bonding: the role of perinatal depression, anxiety and maternal–fetal bonding during pregnancy. Arch Womens Ment Health.

[19] Grigoriadis S (2013). The impact of maternal depression during pregnancy on perinatal outcomes: a systematic review and meta-analysis. J Clin Psychiatry.

[20] J. Barrett and A. S. Fleming, Annual research review: all mothers are not created equal: neural and psychobiological perspectives on mothering and the importance of individual differences, J Child Psychol Psychiatry, 52, 4, 368–397, Apr. 2011, 10.1111/j.1469-7610.2010.02306.x. 10.1111/j.1469-7610.2010.02306.x 20925656

[21] Feldman R, Granat A, Pariente C, Kanety H, Kuint J, Gilboa-Schechtman E (2009). Maternal depression and anxiety across the postpartum year and infant social engagement, fear regulation, and stress reactivity. J Am Acad Child Adolesc Psychiatry.

[22] Mental Health Commission of Canada (2013). Making the case for investing in mental health.

[23] Byatt N, Xiao RS, Dinh KH, Waring ME (2016). Mental health care use in relation to depressive symptoms among pregnant women in the USA. Arch Womens Ment Health.

[24] Thombs BD (2017). Consistency and sources of divergence in recommendations on screening with questionnaires for presently experienced health problems or symptoms: a comparison of recommendations from the Canadian task force on preventive health care, UK National Screening Committee, and US preventive services task force. BMC Med.

[25] Hamel C (2019). Screening for depression in women during pregnancy or the first year postpartum and in the general adult population: a protocol for two systematic reviews to update a guideline of the Canadian task force on preventive health care. Systematic Reviews.

[26] Page MJ (2021). The PRISMA 2020 statement: an updated guideline for reporting systematic reviews. BMJ.

[27] McGowan J, Sampson M, Salzwedel DM, Cogo E, Foerster V, Lefebvre C (2016). PRESS peer review of electronic search strategies: 2015 guideline statement. J Clin Epidemiol.

[28] Thombs BD (2014). Depression screening and patient outcomes in pregnancy or postpartum: a systematic review. J Psychosom Res.

[29] CADTH, Grey Matters: a practical tool for searching health-related grey literature, 2018, Accessed: 25 Apr 2019. [Online]. Available: https://www.cadth.ca/resources/finding-evidence

[30] Robinson KA (2014). Integration of existing systematic reviews into new reviews: identification of guidance needs. Systematic Reviews.

[31] The EndNote Team (2020). EndNote.

[32] Thomson Reuters, Reference Manager 12. 2011. [Online]. Available: http://scientific.thomsonreuters.com/index.html

[33] DistillerSR. Ottawa: Evidence Partners, 2011.

[34] Khangura S, Konnyu K, Cushman R, Grimshaw J, Moher D (2012). Evidence summaries: the evolution of a rapid review approach. Syst Rev.

[35] Higgins J, Green S (2008). Cochrane handbook for systematic reviews of interventions.

[36] Balshem H (2011). GRADE guidelines: 3. Rating the quality of evidence. J Clin Epidemiol.

[37] Balshem H (2013). Finding grey literature evidence and assessing for outcome and analysis reporting biases when comparing medical interventions: AHRQ and the effective health care program. Methods guide for effectiveness and comparative effectiveness reviews.

[38] Higgins J, Green S (2011). Chapter 7: selecting studies and collecting data. The Cochrane collaboration. Cochrane handbook for systematic reviews of interventions, 5.1.0.

[39] Review Manager (RevMan). Copenhagen: The Nordic Cochrane Centre, The Cochrane Collaboration, 2014.

[40] Guyatt GH (2013). GRADE guidelines: 13. Preparing summary of findings tables and evidence profiles-continuous outcomes. J Clin Epidemiol.

[41] Canadian Task for on Preventive Health Care Procedure Manual, (2014). [Online Video]. Available: http://canadiantaskforce.ca/files/procedural-manual-en.pdf

[42] D. Atkins et al., Grading quality of evidence and strength of recommendations, BMJ, 328, 7454, 1490, Jun. 2004, 10.1136/bmj.328.7454.1490. 10.1136/bmj.328.7454.1490 PMC428525 15205295

[43] Murad MH, Wang Z (2017). Guidelines for reporting meta-epidemiological methodology research. Evid Based Med.

[44] Ryan, R and Hill, S, How to GRADE the quality of evidence, La Trobe University, Melbourne. 2016. Accessed: 17 July 2019. [Online]. Available: http://cccrg.cochrane.org/author-resources

[45] Guyatt GH (2011). GRADE guidelines 6. Rating the quality of evidence--imprecision. J Clin Epidemiol.

[46] Kronish IM (2020). Effect of depression screening after acute coronary syndromes on quality of life: the CODIACS-QoL randomized clinical trial. JAMA Intern Med.

[47] Mallen CD (2017). The effects of implementing a point-of-care electronic template to prompt routine anxiety and depression screening in patients consulting for osteoarthritis (the primary care osteoarthritis trial): a cluster randomised trial in primary care. PLoS Med.

[48] Leung SSL (2011). Outcome of a postnatal depression screening programme using the Edinburgh postnatal depression scale: a randomized controlled trial. J Public Health (Oxf).

[49] Thombs BD (2012). Postpartum depression screening: a comment on Leung et al. J Public Health (Oxf).

[50] Cox JL, Holden JM, Sagovsky R (1987). Detection of postnatal depression. Development of the 10-item Edinburgh postnatal depression scale. [document used for perinatal services BC - Edinburgh perinatal/postnatal depression scale (EPDS)]. Br J Psychiatry.

[51] Zwarenstein M (2008). Improving the reporting of pragmatic trials: an extension of the CONSORT statement. BMJ.

[52] National Institute for Health and Care Excellence (NICE), Depression in adults with a chronic physical health problem: recognition and management. NICE clinical guideline 91, UK, 2009. Accessed: 24 July 2019. [Online]. Available: https://www.nice.org.uk/guidance/cg91 39808015

[53] Shrestha SD, Pradhan R, Tran TD, Gualano RC, Fisher JRW. Reliability and validity of the Edinburgh postnatal depression scale (EPDS) for detecting perinatal common mental disorders (PCMDs) among women in low-and lower-middle-income countries: a systematic review. BMC Pregnancy Childbirth. 2016;16. 10.1186/s12884-016-0859-2. 10.1186/s12884-016-0859-2 PMC4820998 27044437

[54] Matthey S, Vedova AMD, Agostini F (2017). The Edinburgh postnatal depression scale in routine screening: errors and cautionary advice. Am J Obstet Gynecol.

[55] O’Connor E (2016). Screening for depression in adults: an updated systematic evidence review for the U.S. preventive services task force.

[56] Williams JW (1999). Case-finding for depression in primary care: a randomized trial. Am J Med.

[57] G. Pittam and M. Allaby, Appraisal of screening for depression. A report for the UK National Screening Committee, 2014. Accessed: 4 Apr 2018. [Online]. Available: https://legacyscreening.phe.org.uk/depression

[58] Myers ER (2013). Efficacy and safety of screening for postpartum depression.

[59] Yawn BP (2012). TRIPPD: a practice-based network effectiveness study of postpartum depression screening and management. Ann Fam Med.

[60] Morrell CJ (2009). Clinical effectiveness of health visitor training in psychologically informed approaches for depression in postnatal women: pragmatic cluster randomised trial in primary care. BMJ.

[61] MacArthur C (2002). Effects of redesigned community postnatal care on womens’ health 4 months after birth: a cluster randomised controlled trial. Lancet.

[62] Wickberg B, Tjus T, Hwang P (2005). Using the EPDS in routine antenatal care in Sweden: a naturalistic study. J Reprod Infant Psychol.

[63] Zlotnick C, Miller IW, Pearlstein T, Howard M, Sweeney P (2006). A preventive intervention for pregnant women on public assistance at risk for postpartum depression. Am J Psychiatry.

[64] Cobo E (2011). Effect of using reporting guidelines during peer review on quality of final manuscripts submitted to a biomedical journal: masked randomised trial. BMJ.

[65] Stevens A (2014). Relation of completeness of reporting of health research to journals’ endorsement of reporting guidelines: systematic review. BMJ.

[66] Goldacre B (2016). Make journals report clinical trials properly. Nature News.

[67] Heneghan C, Goldacre B, Mahtani KR (2017). Why clinical trial outcomes fail to translate into benefits for patients. Trials.

[68] Moher D (2010). CONSORT 2010 explanation and elaboration: updated guidelines for reporting parallel group randomised trials. BMJ.

[69] Arroll B, Khin N, Kerse N (2003). Screening for depression in primary care with two verbally asked questions: cross sectional study. BMJ.

[70] Arroll B, Smith FG, Kerse N, Fishman T, Gunn J (2005). Effect of the addition of a ‘help’ question to two screening questions on specificity for diagnosis of depression in general practice: diagnostic validity study. BMJ.

[71] Arroll B (2010). Validation of PHQ-2 and PHQ-9 to screen for major depression in the primary care population. Ann Fam Med.

