# Supplementary material for: Screening for depression among the general adult population and in women during pregnancy or the first-year postpartum: two systematic reviews to inform a guideline of the Canadian Task Force on Preventive Health Care
Source: Syst Rev. 2022 Aug 22;11:176. doi: 10.1186/s13643-022-02022-2 (PMC9396828; doi:10.1186/s13643-022-02022-2)
Supplement: Supplementary file 1 — Additional file 1. Depression additional files. [file 13643_2022_2022_MOESM1_ESM.docx]

## PRISMA 2020 statement checklist

| **Section and Topic** | **Item #** | **Checklist item** | **Location where item is reported** |
| --- | --- | --- | --- |
| **TITLE** | | |  |
| Title | 1 | Identify the report as a systematic review. | 1 |
| **ABSTRACT** | | |  |
| Abstract | 2 | See the PRISMA 2020 for Abstracts checklist. | 3-4 |
| **INTRODUCTION** | | |  |
| Rationale | 3 | Describe the rationale for the review in the context of existing knowledge. | 5-6 |
| Objectives | 4 | Provide an explicit statement of the objective(s) or question(s) the review addresses. | 6-7 |
| **METHODS** | | |  |
| Eligibility criteria | 5 | Specify the inclusion and exclusion criteria for the review and how studies were grouped for the syntheses. | 9 |
| Information sources | 6 | Specify all databases, registers, websites, organisations, reference lists and other sources searched or consulted to identify studies. Specify the date when each source was last searched or consulted. | 9-11, Additional file |
| Search strategy | 7 | Present the full search strategies for all databases, registers and websites, including any filters and limits used. | Additional file |
| Selection process | 8 | Specify the methods used to decide whether a study met the inclusion criteria of the review, including how many reviewers screened each record and each report retrieved, whether they worked independently, and if applicable, details of automation tools used in the process. | 11-12 |
| Data collection process | 9 | Specify the methods used to collect data from reports, including how many reviewers collected data from each report, whether they worked independently, any processes for obtaining or confirming data from study investigators, and if applicable, details of automation tools used in the process. | 12-13 |
| Data items | 10a | List and define all outcomes for which data were sought. Specify whether all results that were compatible with each outcome domain in each study were sought (e.g. for all measures, time points, analyses), and if not, the methods used to decide which results to collect. | 8,9 |
|  | 10b | List and define all other variables for which data were sought (e.g. participant and intervention characteristics, funding sources). Describe any assumptions made about any missing or unclear information. | 12-13 |
| Study risk of bias assessment | 11 | Specify the methods used to assess risk of bias in the included studies, including details of the tool(s) used, how many reviewers assessed each study and whether they worked independently, and if applicable, details of automation tools used in the process. | 13 |
| Effect measures | 12 | Specify for each outcome the effect measure(s) (e.g. risk ratio, mean difference) used in the synthesis or presentation of results. | 13-15 |
| Synthesis methods | 13a | Describe the processes used to decide which studies were eligible for each synthesis (e.g. tabulating the study intervention characteristics and comparing against the planned groups for each synthesis (item #5)). | 13-15 |
|  | 13b | Describe any methods required to prepare the data for presentation or synthesis, such as handling of missing summary statistics, or data conversions. | 13-15 |
|  | 13c | Describe any methods used to tabulate or visually display results of individual studies and syntheses. | 13-15 |
|  | 13d | Describe any methods used to synthesize results and provide a rationale for the choice(s). If meta-analysis was performed, describe the model(s), method(s) to identify the presence and extent of statistical heterogeneity, and software package(s) used. | 13-15 |
|  | 13e | Describe any methods used to explore possible causes of heterogeneity among study results (e.g. subgroup analysis, meta-regression). | 14 |
|  | 13f | Describe any sensitivity analyses conducted to assess robustness of the synthesized results. | 14 |
| Reporting bias assessment | 14 | Describe any methods used to assess risk of bias due to missing results in a synthesis (arising from reporting biases). | n/a |
| Certainty assessment | 15 | Describe any methods used to assess certainty (or confidence) in the body of evidence for an outcome. | 14-5 |
| **RESULTS** | | |  |
| Study selection | 16a | Describe the results of the search and selection process, from the number of records identified in the search to the number of studies included in the review, ideally using a flow diagram. | 15, 23 |
|  | 16b | Cite studies that might appear to meet the inclusion criteria, but which were excluded, and explain why they were excluded. | 28 |
| Study characteristics | 17 | Cite each included study and present its characteristics. | 16-18, 23 |
| Risk of bias in studies | 18 | Present assessments of risk of bias for each included study. | Additional file 5.4, 6.4 |
| Results of individual studies | 19 | For all outcomes, present, for each study: (a) summary statistics for each group (where appropriate) and (b) an effect estimate and its precision (e.g. confidence/credible interval), ideally using structured tables or plots. | Additional file 5.2, 5.3, 6.2, 6.3 |
| Results of syntheses | 20a | For each synthesis, briefly summarise the characteristics and risk of bias among contributing studies. | 18-22, 24-27,  Additional file 5.4, 6.4 |
|  | 20b | Present results of all statistical syntheses conducted. If meta-analysis was done, present for each the summary estimate and its precision (e.g. confidence/credible interval) and measures of statistical heterogeneity. If comparing groups, describe the direction of the effect. | n/a |
|  | 20c | Present results of all investigations of possible causes of heterogeneity among study results. | n/a |
|  | 20d | Present results of all sensitivity analyses conducted to assess the robustness of the synthesized results. | n/a |
| Reporting biases | 21 | Present assessments of risk of bias due to missing results (arising from reporting biases) for each synthesis assessed. | n/a |
| Certainty of evidence | 22 | Present assessments of certainty (or confidence) in the body of evidence for each outcome assessed. | 18-22, 24-27, Additional file 5.5, 5.6, 6.5, 6.5 |
| **DISCUSSION** | | |  |
| Discussion | 23a | Provide a general interpretation of the results in the context of other evidence. | 29-31 |
|  | 23b | Discuss any limitations of the evidence included in the review. | 27-29 |
|  | 23c | Discuss any limitations of the review processes used. | 32-33 |
|  | 23d | Discuss implications of the results for practice, policy, and future research. | 31-33 |
| **OTHER INFORMATION** | | |  |
| Registration and protocol | 24a | Provide registration information for the review, including register name and registration number, or state that the review was not registered. | 8 |
|  | 24b | Indicate where the review protocol can be accessed, or state that a protocol was not prepared. | 8 |
|  | 24c | Describe and explain any amendments to information provided at registration or in the protocol. | 9 |
| Support | 25 | Describe sources of financial or non-financial support for the review, and the role of the funders or sponsors in the review. | 36 |
| Competing interests | 26 | Declare any competing interests of review authors. | 36 |
| Availability of data, code and other materials | 27 | Report which of the following are publicly available and where they can be found: template data collection forms; data extracted from included studies; data used for all analyses; analytic code; any other materials used in the review. | n/a |

## Additional file 2. Completed PRESS forms for searches

**General adult population**

***PRESS Guideline* 2015— Search Submission & Peer Review Assessment**

Reference: McGowan J, Sampson M, Salzwedel DM, Cogo E, Foerster V, Lefebvre C. PRESS Peer Review of Electronic Search Strategies: 2015 guideline statement. *J Clin Epidemiol* 2016;75:40-6. Available: <http://www.jclinepi.com/article/S0895-4356(16)00058-5/pdf>

**Search submission: This section to be filled in by the searcher**

Searcher: Becky Skidmore Email: [bskidmore@rogers.com](mailto:bskidmore@rogers.com)

Date submitted: 9 Aug 2018 Date requested by: 13 Aug 2018 AM

| **Systematic Review Title** |  |
| --- | --- |

Screening for depression in women during pregnancy or the first-year postpartum and in the general adult population: a protocol for two systematic reviews to update a guideline of the Canadian Task Force on Preventive Health Care

| **This search strategy is …** |
| --- |

| X | My PRIMARY (core) database strategy — First time submitting a strategy for search question and database |
| --- | --- |
|  | My PRIMARY (core) strategy — Follow-up review NOT the first time submitting a strategy for search question and database. If this is a response to peer review, itemize the changes made to the review suggestions |
|  | SECONDARY search strategy— First time submitting a strategy for search question and database |
|  | SECONDARY search strategy — NOT the first time submitting a strategy for search question and database. If  this is a response to peer review, itemize the changes made to the review suggestions |

| **Database** (e.g., MEDLINE, CINAHL) *[mandatory]* |
| --- |

MEDLINE

| **Interface** (e.g., Ovid, EbscoHost…) *[mandatory]* |
| --- |

Ovid

| **Research Question** (Describe the purpose of the search)  *[mandatory]* |
| --- |

What are the benefits and harms of screening for depression in the general adult population in primary care or other non-mental health clinic settings?

What are the benefits and harms of screening for depression in the general adult population in primary care or other non-mental health clinic settings for patients targeted because they have characteristics that may suggest elevated risk of depression? (Characteristics as defined in primary studies, not including exclusion criteria).

| **PICO Format** Outline the PICOs for your question — i.e., Patient, Intervention, Comparison, Outcome, and Study Design — as applicable |
| --- |

| **P** | Key Question 1: Patients who are 18 years and older.  Key Question 1a: Patients who are 18 years and older selected for screening because they have characteristics that may suggest elevated risk of depression*  *characteristics as defined in primary studies (e.g., trauma early in life, a family history of depression) |
| --- | --- |
| **I / Exposure** | Interventions that use a single question, small sets of questions, or a screening questionnaire (validated or non-validated) with a pre-defined cut-off score to identify patients who may have depression, but who have not reported their symptoms to healthcare providers or who have otherwise not been identified as possibly depressed by healthcare providers. |
| **C** | No depression screening.  Patients in comparator trial arms may be administered depression symptom questionnaires for the purpose of baseline or outcome assessments as long as scores are not provided to the patients or healthcare providers. |
| **O** | Symptoms of depression (continuous or dichotomous) or diagnosis of MDD (using a validated diagnostic interview)  Health-related Quality of life  Day-to-day functionality  Lost time at work/school  Impact on lifestyle behaviour (alcohol abuse, smoking, drugs, gambling, etc.)  Suicidality (suicide ideation, attempt or completion)  False positive result (positive screen in absence of depressive disorder), overdiagnosis, or overtreatment  Harms of treatment |
| **S** | Randomized controlled trials (RCTs)* including cluster-controlled trials |

| **Inclusion Criteria** (List criteria such as age groups, study designs, etc., to be included) *[optional]*  **This search strategy is …** |
| --- |

Setting: Primary care or other non-mental health clinic settings, including specialty clinics such as rheumatology, obstetrics and gynecology.

Published from May 2012

| **Exclusion Criteria** (List criteria such as study designs, date limits, etc., to be excluded) **[optional]** |
| --- |

Non-RCTs, controlled before-after, interrupted times series, cohort studies, case-control studies, cross-sectional studies, case series, case reports, and other publication types (editorials, commentaries, notes, letter, opinions).

| **Was a search filter applied?** Yes |
| --- |

**If YES, which one(s) (e.g., Cochrane RCT filter, PubMed Clinical Queries filter)? Provide the source if this is a published filter.** *[mandatory if YES to previous question* — *textbox]*

A randomized controlled trial (RCT) filter based on the Cochrane Highly Sensitive Search Strategy, sensitivity- and precision-maximizing version (2008 revision)

| **Notes or comments you feel would be useful for the peer reviewer**  *[optional]* |
| --- |

Original strategies included instruments/scales – these have since been removed:

“…have been asked to run the search without the screening tool names in it. One of the members is an expert in this area and is pretty adamant that it introduces inefficiencies and that we would not be criticized for not including them.”

| **Please copy and paste your search strategy here, exactly as run, including the number of hits per line. [mandatory]** |
| --- |

Database: Ovid MEDLINE(R) and Epub Ahead of Print, In-Process & Other Non-Indexed Citations and Daily <1946 to August 08, 2018>

Search Strategy:

--------------------------------------------------------------------------------

1 exp Depressive Disorder/ (99942)

2 Depression/ (103334)

3 depress*.tw,kf. (412572)

4 dysthym*.tw,kf. (3029)

5 blues.tw,kf. (1667)

6 melanchol*.tw,kf. (2896)

7 MDD.tw,kf. (10593)

8 or/1-7 [DEPRESSION] (454091)

9 Mass Screening/ (94608)

10 (screen* or detect*).tw,kf. (2584990)

11 (identif* or recogni*).ti. (339243)

12 ((early or earlier or earliest) adj5 (identif* or recogni*)).tw,kf. (59724)

13 (case finding? or casefinding?).tw,kf. (4738)

14 or/9-13 [SCREENING] (2910053)

15 8 and 14 [SCREENING FOR DEPRESSION] (46092)

16 (controlled clinical trial or randomized controlled trial or pragmatic clinical trial).pt. (554624)

17 clinical trials as topic.sh. (184466)

18 exp Randomized Controlled Trials as Topic/ (120088)

19 (randomi#ation? or randomi#ed or randomly or RCT$1 or placebo*).tw,kf. (865980)

20 ((singl* or doubl* or trebl* or tripl*) adj (mask* or blind* or dumm*)).tw,kf. (158451)

21 trial.ti. (185878)

22 or/16-21 (1268696)

23 15 and 22 [SCREENING FOR DEPRESSION - RCTS] (5193)

24 exp Adult/ (6610356)

25 (adult or adults or adulthood).tw,kf. (1094326)

26 (man or men or woman or women).tw,kf. (1487118)

27 middle-age?.tw,kf. (44716)

28 age?.tw,kf. (2362190)

29 (elderly or geriatric* or gerontolog* or old-age? or senior?).tw,kf. (320867)

30 (older adj2 (female? or male? or patient? or person? or people? or population?)).tw,kf. (101493)

31 or/24-30 [ADULTS] (8525239)

32 23 and 31 [SCREENING FOR DEPRESSION - ADULTS - RCTS] (3961)

33 exp Child/ not (exp Adult/ and exp Child/) (1128589)

34 exp Child/ not (Adolescent/ and exp Child/) (926347)

35 exp Infant/ not (exp Adult/ and exp Infant/) (787775)

36 exp Infant/ not (Adolescent/ and exp infant/) (779427)

37 or/33-36 (1804652)

38 32 not 37 [CHILD-/INFANT-ONLY REMOVED] (3760)

39 exp Animals/ not (exp Animals/ and Humans/) (4485646)

40 38 not 39 [ANIMAL-ONLY REMOVED] (3705)

41 (comment or editorial or interview or news or newspaper article).pt. (1275520)

42 (letter not (letter and randomized controlled trial)).pt. (990782)

43 40 not (41 or 42) [OPINION PIECES REMOVED] (3699)

44 limit 43 to yr="2012-current" (1686)

***************************

|  | Reviewer: Kaitryn Campbell | Email: [kcamlolo668@gmail.com](mailto:kcamlolo668@gmail.com) | Date completed: 12 Aug 2018 |
| --- | --- | --- | --- |
|  |  |  |  |

**Peer review assessment: this section to be filled in by the reviewer**

Do you wish to be acknowledged? (If yes, the review team will be advised to add an acknowledgement to any publications related to this work). No – unless your organization requires it

The suggested acknowledgement is “We thank Xxxxx Yyyyyy, MLIS, AHIP (xxxxx Health Sciences Library, University of xxxxxx) for peer review of the MEDLINE search strategy.” [please edit to indicate your name, postnomials and institutional affiliation as you would like them presented].

|  | **1. TRANSLATION** |  |  | | |
| --- | --- | --- | --- | --- | --- |
|  | |  |  |  |  |
| A -­‐No revisions | | x |  |  |  |
| B -­‐ Revision(s) suggested | |  |  |  |  |
| C -­‐ Revision(s) required | |  |  |  |  |

If “B” or “C,” please provide an explanation or example:

**2. BOOLEAN AND PROXIMITY OPERATORS**

| A -­‐No revisions | x |
| --- | --- |
| B -­‐ Revision(s) suggested |  |
| C -­‐ Revision(s) required |  |

If “B” or “C,” please provide an explanation or example:

**3. SUBJECT HEADINGS**

| A -­‐No revisions | x |
| --- | --- |
| B -­‐ Revision(s) suggested |  |
| C -­‐ Revision(s) required |  |

If “B” or “C,” please provide an explanation or example:

**4. TEXT WORD SEARCHING**

| A -­‐No revisions | x |
| --- | --- |
| B -­‐ Revision(s)suggested |  |
| C -­‐ Revision(s) required |  |

If “B” or “C,” please provide an explanation or example:

**5. SPELLING, SYNTAX, AND LINE NUMBERS**

| A -­‐No revisions | x |
| --- | --- |
| B -­‐ Revision(s)suggested |  |
| C -­‐ Revision(s) required |  |

If “B” or “C,” please provide an explanation or example:

**6. LIMITS AND FILTERS**

| A -­‐No revisions | x |
| --- | --- |
| B -­‐ Revision(s) suggested |  |
| C -­‐ Revision(s) required |  |

If “B” or “C,” please provide an explanation or example:

OVERALL EVALUATION (Note: If one or more “revision required” is noted above, the response below must be “revisions required”.)

| A -­‐No revisions | x |
| --- | --- |
| B -­‐ Revision(s) suggested |  |
| C -­‐ Revision(s) required |  |

Additional comments:

Solid search. No corrections/additions.

**Pregnant and Postpartum women**

***PRESS Guideline* 2015— Search Submission & Peer Review Assessment**

Reference: McGowan J, Sampson M, Salzwedel DM, Cogo E, Foerster V, Lefebvre C. PRESS Peer Review of Electronic Search Strategies: 2015 guideline statement. *J Clin Epidemiol* 2016;75:40-6. Available: <http://www.jclinepi.com/article/S0895-4356(16)00058-5/pdf>.

**Search submission: This section to be filled in by the searcher**

Searcher: Becky Skidmore Email: [bskidmore@rogers.com](mailto:bskidmore@rogers.com)

Date submitted: 9 Aug 2018 Date requested by: 13 Aug 2018 AM

| **Systematic Review Title** |  |
| --- | --- |

Screening for depression in women during pregnancy or the first-year postpartum and in the general adult population: a protocol for two systematic reviews to update a guideline of the Canadian Task Force on Preventive Health Care

| **This search strategy is …** | | | |
| --- | --- | --- | --- |
| X | My PRIMARY (core) database strategy — First time submitting a strategy for search question and database |  |  |
|  | My PRIMARY (core) strategy — Follow-up review NOT the first time submitting a strategy for search question and database. If this is a response to peer review, itemize the changes made to the review suggestions |  |  |
|  | SECONDARY search strategy— First time submitting a strategy for search question and database |  |  |
|  | SECONDARY search strategy — NOT the first time submitting a strategy for search question and database. If  this is a response to peer review, itemize the changes made to the review suggestions |  |  |

| **Database** (e.g., MEDLINE, CINAHL) *[mandatory]* |
| --- |

MEDLINE

| **Interface** (e.g., Ovid, EbscoHost…) *[mandatory]* |
| --- |

Ovid

| **Research Question** (Describe the purpose of the search)  *[mandatory]* |
| --- |

**Key Question 1**: What are the benefits and harms of screening for depression during pregnancy and up to one-year postpartum in primary care or other non-mental health clinic settings?

**Key Question 1a**: What are the benefits and harms of screening for depression during pregnancy and up to one-year postpartum in primary care or other non-mental health clinic settings for patients targeted because they have characteristics that may suggest elevated risk of depression? (Characteristics as defined in primary studies, not including exclusion criteria (e.g., previous depression in pregnancy or postpartum)).

| **PICO Format** Outline the PICOs for your question — i.e., Patient, Intervention, Comparison, Outcome, and Study Design — as applicable |
| --- |

| **P** | Key Question 1: Patients during pregnancy and up to one-year postpartum of any age  Key Question 1a. Patients during pregnancy and up to one-year postpartum selected for screening because they have characteristics that may suggest elevated risk of depression*  *characteristics as defined in primary studies (e.g., trauma early in life, a family history of depression) |
| --- | --- |
| **I / Exposure** | Interventions that use a single question, small sets of questions, or a screening questionnaire (validated or non-validated) with a pre-defined cut-off score to identify patients who may have depression, but who have not reported their symptoms to healthcare providers or who have otherwise not been identified as possibly depressed by healthcare providers. |
| **C** | No depression screening.  Patients in comparator trial arms may be administered depression symptom questionnaires for the purpose of baseline or outcome assessments as long as scores are not provided to the patients or healthcare providers. |
| **O** | Symptoms of depression (continuous or dichotomous) or diagnosis of MDD (using a validated diagnostic interview)  Health-related Quality of life  Day-to-day functionality  Lost time at work/school  Impact on lifestyle behaviour (alcohol abuse, smoking, drugs, gambling, etc.)  Suicidality (suicide ideation, attempt or completion)  False positive result (positive screen in absence of depressive disorder), overdiagnosis, or overtreatment  Harms of treatment |
| **S** | Randomized controlled trials (RCTs)* including cluster-controlled trials |

| **Inclusion Criteria** (List criteria such as age groups, study designs, etc., to be included) *[optional]*  **This search strategy is …** |
| --- |

Setting: Primary care or other non-mental health clinic settings, including specialty clinics such as rheumatology, obstetrics and gynecology.

| **Exclusion Criteria** (List criteria such as study designs, date limits, etc., to be excluded) **[optional]** |
| --- |

Non-RCTs, controlled before-after, interrupted times series, cohort studies, case-control studies, cross-sectional studies, case series, case reports, and other publication types (editorials, commentaries, notes, letter, opinions).

| **Was a search filter applied?** Yes |
| --- |

**If YES, which one(s) (e.g., Cochrane RCT filter, PubMed Clinical Queries filter)? Provide the source if this is a published filter.** *[mandatory if YES to previous question* — *textbox]*

A randomized controlled trial (RCT) filter based on the Cochrane Highly Sensitive Search Strategy, sensitivity- and precision-maximizing version (2008 revision

| **Notes or comments you feel would be useful for the peer reviewer**  *[optional]* |
| --- |

Original strategies included instruments/scales – these have since been removed:

“…have been asked to run the search without the screening tool names in it. One of the members is an expert in this area and is pretty adamant that it introduces inefficiencies and that we would not be criticized for not including them.”

| **Please copy and paste your search strategy here, exactly as run, including the number of hits per line. [mandatory]** |
| --- |

Database: Ovid MEDLINE(R) and Epub Ahead of Print, In-Process & Other Non-Indexed Citations and Daily <1946 to August 08, 2018>

Search Strategy:

--------------------------------------------------------------------------------

1 exp Depressive Disorder/ (99942)

2 Depression/ (103334)

3 depress*.tw,kf. (412572)

4 dysthym*.tw,kf. (3029)

5 blues.tw,kf. (1667)

6 melanchol*.tw,kf. (2896)

7 MDD.tw,kf. (10593)

8 PND.tw,kf. (4194)

9 PPD.tw,kf. (8811)

10 or/1-9 [GENERAL DEPRESSION] (465621)

11 Mass Screening/ (94608)

12 (screen* or detect*).tw,kf. (2584990)

13 (identif* or recogni*).ti. (339243)

14 ((early or earlier or earliest) adj5 (identif* or recogni*)).tw,kf. (59724)

15 (case finding? or casefinding?).tw,kf. (4738)

16 or/11-15 [GENERAL SCREENING] (2910053)

17 10 and 16 (48320)

18 (controlled clinical trial or randomized controlled trial or pragmatic clinical trial).pt. (554624)

19 clinical trials as topic.sh. (184466)

20 exp Randomized Controlled Trials as Topic/ (120088)

21 (randomi#ation? or randomi#ed or randomly or RCT$1 or placebo*).tw,kf. (865980)

22 ((singl* or doubl* or trebl* or tripl*) adj (mask* or blind* or dumm*)).tw,kf. (158451)

23 trial.ti. (185878)

24 or/18-23 (1268696)

25 17 and 24 [RCTs - DEPRESSION & SCREENING] (5334)

26 exp Pregnancy/ (839732)

27 exp Pregnancy Complications/ (398169)

28 Pregnant Women/ (6844)

29 exp Pregnancy Trimesters/ (38080)

30 pregnan*.tw,kf. (476924)

31 Maternal Health Services/ (12520)

32 Peripartum Period/ (862)

33 exp Perinatal Care/ (8868)

34 Prenatal Care/ (24574)

35 exp Postpartum Period/ (58948)

36 (prenatal* or pre-natal* or antenatal* or ante natal* or antepartum or ante partum or perinatal* or peri-natal* or peripartum or peri-partum or postnatal* or post-natal* or postpartum or post partum or puerperal or puerperium).tw,kf. (317771)

37 Maternal Health/ (777)

38 (maternal* or maternit*).tw,kf. (248749)

39 (expectant mother* or "new mother" or "new mothers" or "mother-to-be" or "mothers-to-be").tw,kf. (4120)

40 or/26-39 [PREGNANCY/ANTENATAL/POSTNATAL PERIOD] (1187029)

41 Male/ not (Female/ and Male/) (2607236)

42 40 not 41 [MALE-ONLY REMOVED] (1166965)

43 exp Child/ not (exp Adult/ or Adolescent/) (765973)

44 exp Infant/ not (exp Adult/ or Adolescent/) (666888)

45 42 not (43 or 44) [CHILD/INFANT-ONLY POPULATION REMOVED] (1012269)

46 25 and 45 [SCREENING FOR DEPRESSION - PREGNANCY/ANTENATAL/POSTNATAL PERIOD] (371)

47 exp Animals/ not (exp Animals/ and Humans/) (4485646)

48 46 not 47 [ANIMAL-ONLY REMOVED] (326)

49 (editorial or news or newspaper article).pt. (672185)

50 (letter not (letter and randomized controlled trial)).pt. (990782)

51 48 not (49 or 50) [OPINION PIECES REMOVED] (325)

***************************

|  | Reviewer: Kaitryn Campbell | Email: [kcamlolo668@gmail.com](mailto:kcamlolo668@gmail.com) | Date completed: 12 Aug 2018 |
| --- | --- | --- | --- |
|  |  |  |  |

**Peer review assessment: this section to be filled in by the reviewer**

Do you wish to be acknowledged? (If yes, the review team will be advised to add an acknowledgement to any publications related to this work). No – unless your organization requires it

The suggested acknowledgement is “We thank Xxxxx Yyyyyy, MLIS, AHIP (xxxxx Health Sciences Library, University of xxxxxx) for peer review of the MEDLINE search strategy.” [please edit to indicate your name, postnomials and institutional affiliation as you would like them presented].

|  | **1. TRANSLATION** |  |  | | |
| --- | --- | --- | --- | --- | --- |
|  | |  |  |  |  |
| A -­‐No revisions | | x |  |  |  |
| B -­‐ Revision(s) suggested | |  |  |  |  |
| C -­‐ Revision(s) required | |  |  |  |  |

If “B” or “C,” please provide an explanation or example:

**2. BOOLEAN AND PROXIMITY OPERATORS**

| A -­‐No revisions | x |
| --- | --- |
| B -­‐ Revision(s) suggested |  |
| C -­‐ Revision(s) required |  |

If “B” or “C,” please provide an explanation or example:

**3. SUBJECT HEADINGS**

| A -­‐No revisions | x |
| --- | --- |
| B -­‐ Revision(s) suggested |  |
| C -­‐ Revision(s) required |  |

If “B” or “C,” please provide an explanation or example:

**4. TEXT WORD SEARCHING**

| A -­‐No revisions | x |
| --- | --- |
| B -­‐ Revision(s)suggested |  |
| C -­‐ Revision(s) required |  |

If “B” or “C,” please provide an explanation or example:

**5. SPELLING, SYNTAX, AND LINE NUMBERS**

| A -­‐No revisions | x |
| --- | --- |
| B -­‐ Revision(s)suggested |  |
| C -­‐ Revision(s) required |  |

If “B” or “C,” please provide an explanation or example:

**6. LIMITS AND FILTERS**

| A -­‐No revisions | x |
| --- | --- |
| B -­‐ Revision(s) suggested |  |
| C -­‐ Revision(s) required |  |

If “B” or “C,” please provide an explanation or example:

OVERALL EVALUATION (Note: If one or more “revision required” is noted above, the response below must be “revisions required”.)

| A -­‐No revisions | x |
| --- | --- |
| B -­‐ Revision(s) suggested |  |
| C -­‐ Revision(s) required |  |

Additional comments:

The search is well-designed with no errors, and a tight set of results. Am just curious if you also tested the search without the population NOT’ing and whether this cut down on volume drastically? We don’t use this technique currently.

## Additional file 3. General adult population search strategy

### **Ovid Multifile (2020 May 11)**

Database: Embase Classic+Embase <1947 to 2020 May 08>, Ovid MEDLINE(R) ALL <1946 to May 08, 2020>, APA PsycInfo <1806 to May Week 1 2020>

Search Strategy:

---------------------------------------------------------------------

1 exp Depressive Disorder/ (587318)

2 Depression/ (511308)

3 depress*.tw,kf. (1406731)

4 dysthym*.tw,kf. (11242)

5 blues.tw,kf. (4817)

6 melanchol*.tw,kf. (12172)

7 MDD.tw,kf. (42782)

8 or/1-7 [DEPRESSION] (1619893)

9 Mass Screening/ (161885)

10 (screen* or detect*).tw,kf. (6935376)

11 (identif* or recogni*).ti. (896293)

12 ((early or earlier or earliest) adj5 (identif* or recogni*)).tw,kf. (188844)

13 (case finding? or casefinding?).tw,kf. (13596)

14 or/9-13 [SCREENING] (7795470)

15 8 and 14 [SCREENING FOR DEPRESSION] (166188)

16 (controlled clinical trial or randomized controlled trial or pragmatic clinical trial).pt. (594688)

17 clinical trials as topic.sh. (191082)

18 exp Randomized Controlled Trials as Topic/ (314138)

19 (randomi#ation? or randomi#ed or randomly or RCT$1 or placebo*).tw,kf. (2531945)

20 ((singl* or doubl* or trebl* or tripl*) adj (mask* or blind* or dumm*)).tw,kf. (440951)

21 trial.ti. (554231)

22 or/16-21 (3168468)

23 15 and 22 [SCREENING FOR DEPRESSION - RCTS] (17594)

24 exp Adult/ (15839677)

25 (adult or adults or adulthood).tw,kf. (3379489)

26 (man or men or woman or women).tw,kf. (4371037)

27 middle-age?.tw,kf. (131822)

28 age?.tw,kf. (7644017)

29 (elderly or geriatric* or gerontolog* or old-age? or senior?).tw,kf. (958556)

30 (older adj2 (female? or male? or patient? or person? or people? or population?)).tw,kf. (320996)

31 or/24-30 [ADULTS] (22625523)

32 23 and 31 [SCREENING FOR DEPRESSION - ADULTS - RCTS] (12478)

33 exp Child/ not (exp Adult/ and exp Child/) (3409877)

34 exp Child/ not (Adolescent/ and exp Child/) (3143768)

35 exp Infant/ not (exp Adult/ and exp Infant/) (1742613)

36 exp Infant/ not (Adolescent/ and exp infant/) (1768294)

37 or/33-36 (4439693)

38 32 not 37 [CHILD-/INFANT-ONLY REMOVED] (11874)

39 exp Animals/ not (exp Animals/ and Humans/) (18180610)

40 38 not 39 [ANIMAL-ONLY REMOVED] (8696)

41 (comment or editorial or interview or news or newspaper article).pt. (2090419)

42 (letter not (letter and randomized controlled trial)).pt. (2183088)

43 40 not (41 or 42) [OPINION PIECES REMOVED] (8690)

44 limit 43 to yr="2012-current" (4293)

45 44 use medall [MEDLINE RECORDS] (2276)

46 exp Depressive Disorder/ (587318)

47 depress*.tw,kw. (1415327)

48 dysthym*.tw,kw. (11346)

49 blues.tw,kw. (4837)

50 melanchol*.tw,kw. (12266)

51 MDD.tw,kw. (42943)

52 or/46-51 [GENERAL DEPRESSION] (1602554)

53 screening/ (297353)

54 mass screening/ (161885)

55 screening test/ (77214)

56 (screen* or detect*).tw,kw. (6946324)

57 (identif* or recogni*).ti. (896293)

58 ((early or earlier or earliest) adj5 (identif* or recogni*)).tw,kw. (188966)

59 (case finding? or casefinding?).tw,kw. (13733)

60 or/53-59 [GENERAL SCREENING] (7836093)

61 depression assessment/ (1860)

62 52 and 60 (167495)

63 61 or 62 [DEPRESSION & SCREENING/ASSESSMENT] (168957)

64 randomized controlled trial/ or controlled clinical trial/ (1385531)

65 exp "clinical trial (topic)"/ (321709)

66 (randomi#ation? or randomi#ed or randomly or RCT$1 or placebo*).tw,kw. (2534131)

67 ((singl* or doubl* or trebl* or tripl*) adj (mask* or blind* or dumm*)).tw,kw. (441112)

68 trial.ti. (554231)

69 or/64-68 (3353037)

70 63 and 69 [RCTs - DEPRESSION & SCREENING/ASSESSMENT] (19057)

71 exp adult/ (15839677)

72 (adult or adults or adulthood).tw,kw. (3386201)

73 (man or men or woman or women).tw,kw. (4372702)

74 middle-age?.tw,kw. (132283)

75 age?.tw,kf. (7644017)

76 (elderly or geriatric* or gerontolog* or old-age? or senior?).tw,kw. (971054)

77 (older adj2 (age? or female? or male? or patient? or person? or people? or population?)).tw,kw. (486700)

78 or/71-77 [ADULT POPULATION] (22629742)

79 70 and 78 [SCREENING FOR DEPRESSION - ADULT POPULATION - RCTs] (13569)

80 exp child/ not (exp adult/ and exp child/) (3409877)

81 exp adolescent/ not (exp adult/ and exp adolescent/) (1207182)

82 fetus/ not (exp adult/ and fetus/) (246112)

83 or/80-82 (4065688)

84 79 not 83 [UNDER-18 POPULATION ONLY REMOVED] (12967)

85 exp animal experimentation/ or exp animal model/ or exp animal experiment/ or nonhuman/ or exp vertebrate/ (51755327)

86 exp human/ or exp human experimentation/ or exp human experiment/ (40670435)

87 85 not 86 (11086649)

88 84 not 87 [ANIMAL-ONLY REMOVED] (12771)

89 editorial.pt. (1179153)

90 letter.pt. not (letter.pt. and randomized controlled trial/) (2177800)

91 88 not (89 or 90) [OPINION PIECES REMOVED] (12762)

92 conference abstract.pt. (3771767)

93 91 not 92 [CONFERENCE ABSTRACTS REMOVED] (11010)

94 limit 93 to yr="2012-CURRENT" (6109)

95 94 use emczd [EMBASE RECORDS] (3184)

96 "Depression (Emotion)"/ (142567)

97 exp Major Depression/ (193311)

98 depress*.tw. (1400815)

99 dysthym*.tw. (11213)

100 blues.tw. (4807)

101 melanchol*.tw. (12127)

102 MDD.tw. (42657)

103 or/96-102 [GENERAL DEPRESSION] (1449571)

104 Screening/ (297353)

105 exp Screening Tests/ (30883)

106 exp Health Screening/ (258834)

107 (screen* or detect*).tw. (6926974)

108 (identif* or recogni*).ti. (896293)

109 ((early or earlier or earliest) adj5 (identif* or recogni*)).tw. (188743)

110 (case finding? or casefinding?).tw. (13521)

111 or/104-110 [GENERAL SCREENING] (7876514)

112 103 and 111 [SCREENING FOR DEPRESSION] (156656)

113 Clinical Trials/ (105129)

114 (randomi#ation? or randomi#ed or randomly or RCT$1 or placebo*).tw. (2529583)

115 ((singl* or doubl* or trebl* or tripl*) adj (mask* or blind* or dumm*)).tw. (440841)

116 trial.ti. (554231)

117 or/113-116 (2819283)

118 112 and 117 [RCTs - SCREENING FOR DEPRESSION] (15819)

119 (adult or adults or adulthood).tw. (3372782)

120 (man or men or woman or women).tw. (4367925)

121 middle-age?.tw. (131277)

122 age?.tw. (7631943)

123 Geriatric Patients/ (13323)

124 (elderly or geriatric* or gerontolog* or old-age? or senior?).tw. (938346)

125 (older adj2 (age? or female? or male? or patient? or person? or people? or population?)).tw. (484705)

126 or/119-125 [ADULT POPULATION] (13065461)

127 118 and 126 (8205)

128 limit 118 to "300 adulthood <age 18 yrs and older>" [Limit not valid in Embase,Ovid MEDLINE(R),Ovid MEDLINE(R) Daily Update,Ovid MEDLINE(R) In-Process,Ovid MEDLINE(R) Publisher; records were retained] (15070)

129 127 or 128 [SCREENING FOR DEPRESSION - ADULTS] (15387)

130 exp Animals/ not (exp Animals/ and Humans/) (18180610)

131 129 not 130 [ANIMAL-ONLY REMOVED] (10904)

132 limit 131 to yr="2012-current" (5573)

133 132 use medall,emczd (4430)

134 132 not 133 [PSYCINFO RECORDS] (1143)

135 45 or 95 or 134 [ALL DATABASES] (6603)

136 135 use medall [MEDLINE RECORDS] (2276)

137 (201810* or 201811* or 201812* or 2019* or 2020*).dt. (2120253)

138 136 and 137 [MEDLINE UPDATE PERIOD] (470)

139 135 use emczd [EMBASE RECORDS] (3184)

140 (201810* or 201811* or 201812* or 2019* or 2020*).dc. (3132108)

141 139 and 140 [EMBASE UPDATE PERIOD] (858)

142 135 not (136 or 139) [PSYCINFO RECORDS] (1143)

143 (201809* or 201810* or 201811* or 201812* or 2019* or 2020*).up. (35074348)

144 142 and 143 [PSYCINFO UPDATE PERIOD] (257)

145 138 or 141 or 144 [ALL DATABASES - UPDATE PERIOD] (1585)

146 remove duplicates from 145 (1066) [TOTAL UNIQUE RECORDS]

147 146 use medall [MEDLINE UNIQUE RECORDS - UPDATE PERIOD] (459)

148 146 use emczd [EMBASE UNIQUE RECORDS - UPDATE PERIOD] (496)

149 146 not (147 or 148) [PSYCINFO UNIQUE RECORDS - UPDATE PERIOD] (111)

### **Ovid Multifile (2018 Oct 4)**

Ovid Multifile: 2018 Sep 25 (*Updated to 2018 Oct 4*)

Database: Embase Classic+Embase <1947 to 2018 September 24>, Ovid MEDLINE(R) ALL <1946 to September 24, 2018>, PsycINFO <1806 to September Week 3 2018>

Search Strategy:

---------------------------------------------------------------------

1 exp Depressive Disorder/ (525659)

2 Depression/ (459247)

3 depress*.tw,kf. (1276494)

4 dysthym*.tw,kf. (10890)

5 blues.tw,kf. (4536)

6 melanchol*.tw,kf. (11670)

7 MDD.tw,kf. (35588)

8 or/1-7 [DEPRESSION] (1473035)

9 Mass Screening/ (151048)

10 (screen* or detect*).tw,kf. (6199421)

11 (identif* or recogni*).ti. (804246)

12 ((early or earlier or earliest) adj5 (identif* or recogni*)).tw,kf. (164364)

13 (case finding? or casefinding?).tw,kf. (12220)

14 or/9-13 [SCREENING] (6975361)

15 8 and 14 [SCREENING FOR DEPRESSION] (145033)

16 (controlled clinical trial or randomized controlled trial or pragmatic clinical trial).pt. (556602)

17 clinical trials as topic.sh. (184793)

18 exp Randomized Controlled Trials as Topic/ (269439)

19 (randomi#ation? or randomi#ed or randomly or RCT$1 or placebo*).tw,kf. (2237960)

20 ((singl* or doubl* or trebl* or tripl*) adj (mask* or blind* or dumm*)).tw,kf. (404403)

21 trial.ti. (473473)

22 or/16-21 (2834719)

23 15 and 22 [SCREENING FOR DEPRESSION - RCTS] (15050)

24 exp Adult/ (14133064)

25 (adult or adults or adulthood).tw,kf. (3011206)

26 (man or men or woman or women).tw,kf. (3990334)

27 middle-age?.tw,kf. (118226)

28 age?.tw,kf. (6807032)

29 (elderly or geriatric* or gerontolog* or old-age? or senior?).tw,kf. (873027)

30 (older adj2 (female? or male? or patient? or person? or people? or population?)).tw,kf. (281007)

31 or/24-30 [ADULTS] (20430617)

32 23 and 31 [SCREENING FOR DEPRESSION - ADULTS - RCTS] (10570)

33 exp Child/ not (exp Adult/ and exp Child/) (3172580)

34 exp Child/ not (Adolescent/ and exp Child/) (2919512)

35 exp Infant/ not (exp Adult/ and exp Infant/) (1647232)

36 exp Infant/ not (Adolescent/ and exp infant/) (1663799)

37 or/33-36 (4131523)

38 32 not 37 [CHILD-/INFANT-ONLY REMOVED] (10079)

39 exp Animals/ not (exp Animals/ and Humans/) (17261236)

40 38 not 39 [ANIMAL-ONLY REMOVED] (7058)

41 (comment or editorial or interview or news or newspaper article).pt. (1860402)

42 (letter not (letter and randomized controlled trial)).pt. (2027319)

43 40 not (41 or 42) [OPINION PIECES REMOVED] (7052)

44 limit 43 to yr="2012-current" (2665)

45 44 use medall [MEDLINE RECORDS] (1717)

46 exp Depressive Disorder/ (525659)

47 depress*.tw,kw. (1284029)

48 dysthym*.tw,kw. (10981)

49 blues.tw,kw. (4553)

50 melanchol*.tw,kw. (11757)

51 MDD.tw,kw. (35725)

52 or/46-51 [GENERAL DEPRESSION] (1456043)

53 screening/ (283438)

54 mass screening/ (151048)

55 screening test/ (70964)

56 (screen* or detect*).tw,kw. (6209418)

57 (identif* or recogni*).ti. (804246)

58 ((early or earlier or earliest) adj5 (identif* or recogni*)).tw,kw. (164481)

59 (case finding? or casefinding?).tw,kw. (12344)

60 or/53-59 [GENERAL SCREENING] (7012951)

61 depression assessment/ (1237)

62 52 and 60 (146201)

63 61 or 62 [DEPRESSION & SCREENING/ASSESSMENT] (147180)

64 randomized controlled trial/ or controlled clinical trial/ (1253703)

65 exp "clinical trial (topic)"/ (273834)

66 (randomi#ation? or randomi#ed or randomly or RCT$1 or placebo*).tw,kw. (2240072)

67 ((singl* or doubl* or trebl* or tripl*) adj (mask* or blind* or dumm*)).tw,kw. (404551)

68 trial.ti. (473473)

69 or/64-68 (3003708)

70 63 and 69 [RCTs - DEPRESSION & SCREENING/ASSESSMENT] (16308)

71 exp adult/ (14133064)

72 (adult or adults or adulthood).tw,kw. (3017285)

73 (man or men or woman or women).tw,kw. (3991732)

74 middle-age?.tw,kw. (118633)

75 age?.tw,kf. (6807032)

76 (elderly or geriatric* or gerontolog* or old-age? or senior?).tw,kw. (883734)

77 (older adj2 (age? or female? or male? or patient? or person? or people? or population?)).tw,kw. (423673)

78 or/71-77 [ADULT POPULATION] (20434612)

79 70 and 78 [SCREENING FOR DEPRESSION - ADULT POPULATION - RCTs] (11502)

80 exp child/ not (exp adult/ and exp child/) (3172580)

81 exp adolescent/ not (exp adult/ and exp adolescent/) (1116653)

82 fetus/ not (exp adult/ and fetus/) (237047)

83 or/80-82 (3789094)

84 79 not 83 [UNDER-18 POPULATION ONLY REMOVED] (11006)

85 exp animal experimentation/ or exp animal model/ or exp animal experiment/ or nonhuman/ or exp vertebrate/ (47851101)

86 exp human/ or exp human experimentation/ or exp human experiment/ (37400820)

87 85 not 86 (10451986)

88 84 not 87 [ANIMAL-ONLY REMOVED] (10846)

89 editorial.pt. (1044960)

90 letter.pt. not (letter.pt. and randomized controlled trial/) (2022423)

91 88 not (89 or 90) [OPINION PIECES REMOVED] (10838)

92 conference abstract.pt. (3151029)

93 91 not 92 [CONFERENCE ABSTRACTS REMOVED] (9469)

94 limit 93 to yr="2012-CURRENT" (4576)

95 94 use emczd [EMBASE RECORDS] (2369)

96 "Depression (Emotion)"/ (128391)

97 exp Major Depression/ (174970)

98 depress*.tw. (1271702)

99 dysthym*.tw. (10872)

100 blues.tw. (4529)

101 melanchol*.tw. (11631)

102 MDD.tw. (35498)

103 or/96-102 [GENERAL DEPRESSION] (1317351)

104 Screening/ (283438)

105 exp Screening Tests/ (24701)

106 exp Health Screening/ (229651)

107 (screen* or detect*).tw. (6193289)

108 (identif* or recogni*).ti. (804246)

109 ((early or earlier or earliest) adj5 (identif* or recogni*)).tw. (164292)

110 (case finding? or casefinding?).tw. (12169)

111 or/104-110 [GENERAL SCREENING] (7046364)

112 103 and 111 [SCREENING FOR DEPRESSION] (136722)

113 Clinical Trials/ (92465)

114 (randomi#ation? or randomi#ed or randomly or RCT$1 or placebo*).tw. (2236103)

115 ((singl* or doubl* or trebl* or tripl*) adj (mask* or blind* or dumm*)).tw. (404314)

116 trial.ti. (473473)

117 or/113-116 (2496825)

118 112 and 117 [RCTs - SCREENING FOR DEPRESSION] (13444)

119 (adult or adults or adulthood).tw. (3006428)

120 (man or men or woman or women).tw. (3987637)

121 middle-age?.tw. (117742)

122 age?.tw. (6796045)

123 Geriatric Patients/ (12823)

124 (elderly or geriatric* or gerontolog* or old-age? or senior?).tw. (854739)

125 (older adj2 (age? or female? or male? or patient? or person? or people? or population?)).tw. (422156)

126 or/119-125 [ADULT POPULATION] (11761784)

127 118 and 126 (6903)

128 limit 118 to "300 adulthood <age 18 yrs and older>" [Limit not valid in Embase,Ovid MEDLINE(R),Ovid MEDLINE(R) Daily Update,Ovid MEDLINE(R) In-Process,Ovid MEDLINE(R) Publisher; records were retained] (12815)

129 127 or 128 [SCREENING FOR DEPRESSION - ADULTS] (13073)

130 exp Animals/ not (exp Animals/ and Humans/) (17261236)

131 129 not 130 [ANIMAL-ONLY REMOVED] (8848)

132 limit 131 to yr="2012-current" (3537)

133 132 use medall,emczd (2654)

134 132 not 133 [PSYCINFO RECORDS] (883)

135 45 or 95 or 134 [ALL DATABASES] (4969)

136 remove duplicates from 135 (2925)

137 136 use medall (1709)

138 136 use emczd (958)

139 136 not (137 or 138) (258)

### **Cochrane Library (2020 May 11)**


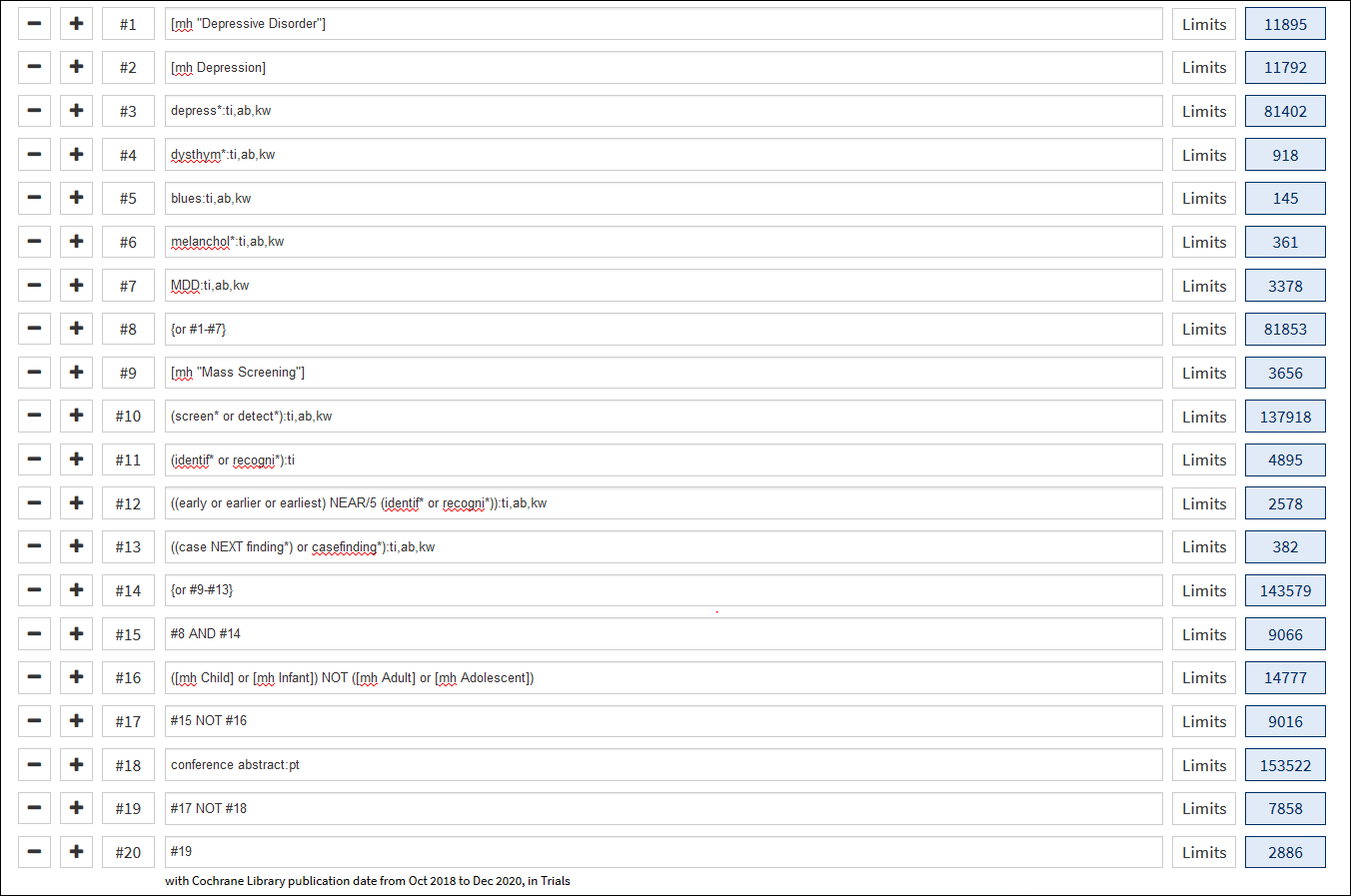


### **Cochrane Library (2018 Oct 4)**

Search Name: CTFPHC - Depression - Screening - Adults

Date Run: 04/10/2018 18:23:34

ID Search Hits

#1 MeSH descriptor: ["Depressive Disorder"] explode all trees 10295

#2 MeSH descriptor: [Depression] explode all trees 9672

#3 depress*:ti,ab,kw 61914

#4 dysthym*:ti,ab,kw 755

#5 blues:ti,ab,kw 97

#6 melanchol*:ti,ab,kw 320

#7 MDD:ti,ab,kw 2560

#8 {or #1-#7} 62283

#9 MeSH descriptor: ["Mass Screening"] explode all trees 2885

#10 (screen* or detect*):ti,ab,kw 104808

#11 (identif* or recogni*):ti 4474

#12 ((early or earlier or earliest) near/5 (identif* or recogni*)):ti,ab,kw 2161

#13 ((case next finding*) or casefinding*):ti,ab,kw 297

#14 {or #9-#13} 109756

#15 #8 and #14 6287

#16 MeSH descriptor: [Child] explode all trees 837

#17 MeSH descriptor: [Child] explode all trees 835

#18 MeSH descriptor: [Infant] explode all trees 14937

#19 MeSH descriptor: [Infant] explode all trees 13248

#20 {or #16-#19} 15919

#21 #15 not #20 6228

#22 conference abstract:pt 121946

#23 #21 not #22 with Publication year from 2012 to 2018 2884

Reviews: 161

Trials: 2719

### **CINAHL (2020 May 11)**

| # | Query | Limiters/Expanders | Results |
| --- | --- | --- | --- |
| S36 | S32 OR S33 | Limiters - Published Date: 20180101-20201231; Exclude MEDLINE records  Expanders - Apply related words  Search modes - Boolean/Phrase | 133 |
| S35 | S32 OR S33 | Limiters - Published Date: 20180101-20201231  Expanders - Apply related words  Search modes - Boolean/Phrase | 349 |
| S34 | S32 OR S33 | Expanders - Apply related words  Search modes - Boolean/Phrase | 1,893 |
| S33 | S29 NOT S31 | Expanders - Apply related words  Search modes - Boolean/Phrase | 1,853 |
| S32 | S29 NOT S30 | Expanders - Apply related words  Search modes - Boolean/Phrase | 1,864 |
| S31 | (MH "Child+") NOT ( ( MH "Child+" AND MH "Adult+") ) | Expanders - Apply related words  Search modes - Boolean/Phrase | 529,599 |
| S30 | (MH "Child+") NOT ( (MH "Child+" AND MH "Adolescence+") ) | Expanders - Apply related words  Search modes - Boolean/Phrase | 466,209 |
| S29 | S21 AND S28 | Expanders - Apply related words  Search modes - Boolean/Phrase | 1,926 |
| S28 | S22 OR S23 OR S24 OR S25 OR S26 OR S27 | Expanders - Apply related words  Search modes - Boolean/Phrase | 2,531,953 |
| S27 | TI ( older N2 (age or aged or ages or female or females or male or males or patient? or person or persons or people or population*) ) OR AB ( older N2 (age or aged or ages or female or females or male or males or patient? or person or persons or people or population*) ) | Expanders - Apply related words  Search modes - Boolean/Phrase | 96,560 |
| S26 | TI ( elderly or geriatric* or gerontolog* or "old-age" or "old-aged" or senior or seniors ) OR AB ( elderly or geriatric* or gerontolog* or "old-age" or "old-aged" or senior or seniors ) | Expanders - Apply related words  Search modes - Boolean/Phrase | 146,786 |
| S25 | TI ( "middle-age" or "middle-aged" or age or ages or aged ) OR AB ( "middle-age" or "middle-aged" or age or aged or ages ) | Expanders - Apply related words  Search modes - Boolean/Phrase | 767,906 |
| S24 | TI ( man or men or woman or women ) OR AB ( man or men or woman or women ) | Expanders - Apply related words  Search modes - Boolean/Phrase | 540,172 |
| S23 | TI ( adult or adults or adulthood ) OR AB ( adult or adults or adulthood ) | Expanders - Apply related words  Search modes - Boolean/Phrase | 375,119 |
| S22 | (MH "Adult+") | Expanders - Apply related words  Search modes - Boolean/Phrase | 1,905,792 |
| S21 | S14 AND S20 | Expanders - Apply related words  Search modes - Boolean/Phrase | 2,551 |
| S20 | S15 OR S16 OR S17 OR S18 OR S19 | Expanders - Apply related words  Search modes - Boolean/Phrase | 399,870 |
| S19 | TI trial | Expanders - Apply related words  Search modes - Boolean/Phrase | 111,730 |
| S18 | TI ( ((singl* or doubl* or trebl* or tripl*) N1 (mask* or blind* or dumm*)) ) OR AB ( ((singl* or doubl* or trebl* or tripl*) N1 (mask* or blind* or dumm*)) ) | Expanders - Apply related words  Search modes - Boolean/Phrase | 50,868 |
| S17 | TI ( randomi?ed or randomly or RCT or RCTs or placebo* ) OR AB ( randomi?ed or randomly or RCT or RCTs or placebo* ) | Expanders - Apply related words  Search modes - Boolean/Phrase | 336,728 |
| S16 | (MH "Double-Blind Studies") OR (MH "Single-Blind Studies") OR (MH "Triple-Blind Studies") | Expanders - Apply related words  Search modes - Boolean/Phrase | 64,816 |
| S15 | (MH "Randomized Controlled Trials") | Expanders - Apply related words  Search modes - Boolean/Phrase | 118,438 |
| S14 | S7 AND S13 | Expanders - Apply related words  Search modes - Boolean/Phrase | 22,477 |
| S13 | S8 OR S9 OR S10 OR S11 OR S12 | Expanders - Apply related words  Search modes - Boolean/Phrase | 468,145 |
| S12 | TI ( "case finding" or "case findings" or casefinding or casefindings ) OR AB ( "case finding" or "case findings" or casefinding or casefindings ) | Expanders - Apply related words  Search modes - Boolean/Phrase | 1,588 |
| S11 | TI ( ((early or earlier or earliest) N5 (identif* or recogni*)) ) OR AB ( ((early or earlier or earliest) N5 (identif* or recogni*)) ) | Expanders - Apply related words  Search modes - Boolean/Phrase | 22,820 |
| S10 | TI (identif* or recogni*) | Expanders - Apply related words  Search modes - Boolean/Phrase | 62,534 |
| S9 | TI ( (screen* or detect*) ) OR AB ( (screen* or detect*) ) | Expanders - Apply related words  Search modes - Boolean/Phrase | 382,286 |
| S8 | (MH "Health Screening") | Expanders - Apply related words  Search modes - Boolean/Phrase | 48,758 |
| S7 | S1 OR S2 OR S3 OR S4 OR S5 OR S6 | Expanders - Apply related words  Search modes - Boolean/Phrase | 195,822 |
| S6 | TI MDD OR AB MDD | Expanders - Apply related words  Search modes - Boolean/Phrase | 3,995 |
| S5 | TI melanchol* OR AB melanchol* | Expanders - Apply related words  Search modes - Boolean/Phrase | 499 |
| S4 | TI blues OR AB blues | Expanders - Apply related words  Search modes - Boolean/Phrase | 9,665 |
| S3 | TI dysthym* OR AB dysthym* | Expanders - Apply related words  Search modes - Boolean/Phrase | 838 |
| S2 | TI depress* OR AB depress* | Expanders - Apply related words  Search modes - Boolean/Phrase | 156,217 |
| S1 | (MH "Depression+") | Expanders - Apply related words  Search modes - Boolean/Phrase | 120,272 |

### **CINAHL (2018 Oct 4)**

| # | Query | Limiters/Expanders | Results |
| --- | --- | --- | --- |
| S35 | S32 OR S33 | Limiters - Published Date: 20120101-20181231; Exclude MEDLINE records  Expanders - Apply related words  Search modes - Boolean/Phrase | 113 |
| S34 | S32 OR S33 | Expanders - Apply related words  Search modes - Boolean/Phrase | 864 |
| S33 | S29 NOT S31 | Expanders - Apply related words  Search modes - Boolean/Phrase | 847 |
| S32 | S29 NOT S30 | Expanders - Apply related words  Search modes - Boolean/Phrase | 848 |
| S31 | (MH "Child+") NOT ( ( MH "Child+" AND MH "Adult+") ) | Expanders - Apply related words  Search modes - Boolean/Phrase | 285,658 |
| S30 | (MH "Child+") NOT ( (MH "Child+" AND MH "Adolescence+") ) | Expanders - Apply related words  Search modes - Boolean/Phrase | 256,396 |
| S29 | S21 AND S28 | Expanders - Apply related words  Search modes - Boolean/Phrase | 869 |
| S28 | S22 OR S23 OR S24 OR S25 OR S26 OR S27 | Expanders - Apply related words  Search modes - Boolean/Phrase | 1,222,007 |
| S27 | TI ( older N2 (age or aged or ages or female or females or male or males or patient? or person or persons or people or population*) ) OR AB ( older N2 (age or aged or ages or female or females or male or males or patient? or person or persons or people or population*) ) | Expanders - Apply related words  Search modes - Boolean/Phrase | 52,089 |
| S26 | TI ( elderly or geriatric* or gerontolog* or "old-age" or "old-aged" or senior or seniors ) OR AB ( elderly or geriatric* or gerontolog* or "old-age" or "old-aged" or senior or seniors ) | Expanders - Apply related words  Search modes - Boolean/Phrase | 82,654 |
| S25 | TI ( "middle-age" or "middle-aged" or age or ages or aged ) OR AB ( "middle-age" or "middle-aged" or age or aged or ages ) | Expanders - Apply related words  Search modes - Boolean/Phrase | 348,224 |
| S24 | TI ( man or men or woman or women ) OR AB ( man or men or woman or women ) | Expanders - Apply related words  Search modes - Boolean/Phrase | 260,926 |
| S23 | TI ( adult or adults or adulthood ) OR AB ( adult or adults or adulthood ) | Expanders - Apply related words  Search modes - Boolean/Phrase | 178,065 |
| S22 | (MH "Adult+") | Expanders - Apply related words  Search modes - Boolean/Phrase | 934,476 |
| S21 | S14 AND S20 | Expanders - Apply related words  Search modes - Boolean/Phrase | 1,108 |
| S20 | S15 OR S16 OR S17 OR S18 OR S19 | Expanders - Apply related words  Search modes - Boolean/Phrase | 186,312 |
| S19 | TI trial | Expanders - Apply related words  Search modes - Boolean/Phrase | 49,258 |
| S18 | TI ( ((singl* or doubl* or trebl* or tripl*) N1 (mask* or blind* or dumm*)) ) OR AB ( ((singl* or doubl* or trebl* or tripl*) N1 (mask* or blind* or dumm*)) ) | Expanders - Apply related words  Search modes - Boolean/Phrase | 23,339 |
| S17 | TI ( randomi?ed or randomly or RCT or RCTs or placebo* ) OR AB ( randomi?ed or randomly or RCT or RCTs or placebo* ) | Expanders - Apply related words  Search modes - Boolean/Phrase | 155,628 |
| S16 | (MH "Double-Blind Studies") OR (MH "Single-Blind Studies") OR (MH "Triple-Blind Studies") | Expanders - Apply related words  Search modes - Boolean/Phrase | 33,052 |
| S15 | (MH "Randomized Controlled Trials") | Expanders - Apply related words  Search modes - Boolean/Phrase | 43,953 |
| S14 | S7 AND S13 | Expanders - Apply related words  Search modes - Boolean/Phrase | 10,412 |
| S13 | S8 OR S9 OR S10 OR S11 OR S12 | Expanders - Apply related words  Search modes - Boolean/Phrase | 207,189 |
| S12 | TI ( "case finding" or "case findings" or casefinding or casefindings ) OR AB ( "case finding" or "case findings" or casefinding or casefindings ) | Expanders - Apply related words  Search modes - Boolean/Phrase | 730 |
| S11 | TI ( ((early or earlier or earliest) N5 (identif* or recogni*)) ) OR AB ( ((early or earlier or earliest) N5 (identif* or recogni*)) ) | Expanders - Apply related words  Search modes - Boolean/Phrase | 10,843 |
| S10 | TI (identif* or recogni*) | Expanders - Apply related words  Search modes - Boolean/Phrase | 31,342 |
| S9 | TI ( (screen* or detect*) ) OR AB ( (screen* or detect*) ) | Expanders - Apply related words  Search modes - Boolean/Phrase | 162,964 |
| S8 | (MH "Health Screening") | Expanders - Apply related words  Search modes - Boolean/Phrase | 25,530 |
| S7 | S1 OR S2 OR S3 OR S4 OR S5 OR S6 | Expanders - Apply related words  Search modes - Boolean/Phrase | 93,316 |
| S6 | TI MDD OR AB MDD | Expanders - Apply related words  Search modes - Boolean/Phrase | 982 |
| S5 | TI melanchol* OR AB melanchol* | Expanders - Apply related words  Search modes - Boolean/Phrase | 196 |
| S4 | TI blues OR AB blues | Expanders - Apply related words  Search modes - Boolean/Phrase | 4,232 |
| S3 | TI dysthym* OR AB dysthym* | Expanders - Apply related words  Search modes - Boolean/Phrase | 415 |
| S2 | TI depress* OR AB depress* | Expanders - Apply related words  Search modes - Boolean/Phrase | 72,017 |
| S1 | (MH "Depression+") | Expanders - Apply related words  Search modes - Boolean/Phrase |  |

## Additional file 4. Pregnancy and postpartum search strategy

### **Ovid Multifile (2020 May 11)**

Database: Embase Classic+Embase <1947 to 2020 May 08>, Ovid MEDLINE(R) ALL <1946 to May 08, 2020>, APA PsycInfo <1806 to May Week 1 2020>

Search Strategy:

----------------------------------------------------------------------

1 exp Depressive Disorder/ (587318)

2 Depression/ (511308)

3 depress*.tw,kf. (1406731)

4 dysthym*.tw,kf. (11242)

5 blues.tw,kf. (4817)

6 melanchol*.tw,kf. (12172)

7 MDD.tw,kf. (42782)

8 PND.tw,kf. (12857)

9 PPD.tw,kf. (24574)

10 or/1-9 [GENERAL DEPRESSION] (1651708)

11 Mass Screening/ (161885)

12 (screen* or detect*).tw,kf. (6935376)

13 (identif* or recogni*).ti. (896293)

14 ((early or earlier or earliest) adj5 (identif* or recogni*)).tw,kf. (188844)

15 (case finding? or casefinding?).tw,kf. (13596)

16 or/11-15 [GENERAL SCREENING] (7795470)

17 10 and 16 (172301)

18 (controlled clinical trial or randomized controlled trial or pragmatic clinical trial).pt. (594688)

19 clinical trials as topic.sh. (191082)

20 exp Randomized Controlled Trials as Topic/ (314138)

21 (randomi#ation? or randomi#ed or randomly or RCT$1 or placebo*).tw,kf. (2531945)

22 ((singl* or doubl* or trebl* or tripl*) adj (mask* or blind* or dumm*)).tw,kf. (440951)

23 trial.ti. (554231)

24 or/18-23 (3168468)

25 17 and 24 [RCTs - DEPRESSION & SCREENING] (17939)

26 exp Pregnancy/ (1727769)

27 exp Pregnancy Complications/ (567358)

28 Pregnant Women/ (79628)

29 exp Pregnancy Trimesters/ (838768)

30 pregnan*.tw,kf. (1265685)

31 Maternal Health Services/ (15036)

32 Peripartum Period/ (29135)

33 exp Perinatal Care/ (69257)

34 Prenatal Care/ (69999)

35 exp Postpartum Period/ (135903)

36 (prenatal* or pre-natal* or antenatal* or ante natal* or antepartum or ante partum or perinatal* or peri-natal* or peripartum or peri-partum or postnatal* or post-natal* or postpartum or post partum or puerperal or puerperium).tw,kf. (862689)

37 Maternal Health/ (16065)

38 (maternal* or maternit*).tw,kf. (701206)

39 (expectant mother* or "new mother" or "new mothers" or "mother-to-be" or "mothers-to-be").tw,kf. (13394)

40 or/26-39 [PREGNANCY/ANTENATAL/POSTNATAL PERIOD] (2875332)

41 25 and 40 [RCTs - DEPRESSION & SCREENING - PREGNANCY/ANTENATAL/POSTNATAL PERIOD] (1373)

42 exp Animals/ not (exp Animals/ and Humans/) (18180610)

43 41 not 42 [ANIMAL-ONLY REMOVED] (937)

44 (comment or editorial or news or newspaper article).pt. (2062221)

45 (letter not (letter and randomized controlled trial)).pt. (2183088)

46 43 not (44 or 45) [OPINION PIECES REMOVED] (934)

47 46 use medall [MEDLINE RECORDS] (442)

48 exp Depressive Disorder/ (587318)

49 depress*.tw,kw. (1415327)

50 dysthym*.tw,kw. (11346)

51 blues.tw,kw. (4837)

52 melanchol*.tw,kw. (12266)

53 MDD.tw,kw. (42943)

54 PND.tw,kw. (12867)

55 PPD.tw,kw. (24670)

56 or/48-55 [GENERAL DEPRESSION] (1634462)

57 screening/ (297353)

58 mass screening/ (161885)

59 screening test/ (77214)

60 (screen* or detect*).tw,kw. (6946324)

61 (identif* or recogni*).ti. (896293)

62 ((early or earlier or earliest) adj5 (identif* or recogni*)).tw,kw. (188966)

63 (case finding? or casefinding?).tw,kw. (13733)

64 or/57-63 [GENERAL SCREENING] (7836093)

65 depression assessment/ (1860)

66 56 and 64 (173664)

67 65 or 66 [DEPRESSION & SCREENING/ASSESSMENT] (175126)

68 randomized controlled trial/ or controlled clinical trial/ (1385531)

69 exp "clinical trial (topic)"/ (321709)

70 (randomi#ation? or randomi#ed or randomly or RCT$1 or placebo*).tw,kw. (2534131)

71 ((singl* or doubl* or trebl* or tripl*) adj (mask* or blind* or dumm*)).tw,kw. (441112)

72 trial.ti. (554231)

73 or/68-72 (3353037)

74 67 and 73 [RCTs - DEPRESSION & SCREENING/ASSESSMENT] (19441)

75 exp pregnancy/ (1727769)

76 exp pregnancy disorder/ (612624)

77 exp named groups by pregnancy/ (113531)

78 pregnan*.tw,kw. (1275841)

79 exp maternal care/ (46132)

80 perinatal care/ (18677)

81 prenatal care/ (69999)

82 postnatal care/ (12933)

83 perinatal period/ (37680)

84 prenatal period/ (11119)

85 puerperium/ (69494)

86 (prenatal* or pre-natal* or antenatal* or ante natal* or antepartum or ante partum or perinatal* or peri-natal* or peripartum or peri-partum or postnatal* or post-natal* or postpartum or post partum or puerperal or puerperium).tw,kw. (868788)

87 (maternal* or maternit*).tw,kw. (706438)

88 expectant mother/ (1383)

89 (expectant mother* or "new mother" or "new mothers" or "mother-to-be" or "mothers-to-be").tw,kw. (13397)

90 or/75-89 [PREGNANCY/ANTENATAL/POSTNATAL PERIOD] (2982941)

91 74 and 90 [RCTs - DEPRESSION & SCREENING/ASSESSMENT - PREGNANCY/ANTENATAL/POSTNATAL PERIOD] (1483)

92 exp animal experimentation/ or exp animal model/ or exp animal experiment/ or nonhuman/ or exp vertebrate/ (51755327)

93 exp human/ or exp human experimentation/ or exp human experiment/ (40670435)

94 92 not 93 (11086649)

95 91 not 94 [ANIMAL-ONLY REMOVED] (1379)

96 editorial.pt. (1179153)

97 letter.pt. not (letter.pt. and randomized controlled trial/) (2177800)

98 95 not (96 or 97) [OPINION PIECES REMOVED] (1375)

99 conference abstract.pt. (3771767)

100 98 not 99 [CONFERENCE ABSTRACTS REMOVED] (1192)

101 100 use emczd [EMBASE RECORDS] (590)

102 "Depression (Emotion)"/ (142567)

103 exp Major Depression/ (193311)

104 depress*.tw. (1400815)

105 dysthym*.tw. (11213)

106 blues.tw. (4807)

107 melanchol*.tw. (12127)

108 MDD.tw. (42657)

109 PND.tw. (12791)

110 PPD.tw. (24510)

111 or/102-110 [GENERAL DEPRESSION] (1481292)

112 Screening/ (297353)

113 exp Screening Tests/ (30883)

114 exp Health Screening/ (258834)

115 (screen* or detect*).tw. (6926974)

116 (identif* or recogni*).ti. (896293)

117 ((early or earlier or earliest) adj5 (identif* or recogni*)).tw. (188743)

118 (case finding? or casefinding?).tw. (13521)

119 or/112-118 [GENERAL SCREENING] (7876514)

120 111 and 119 [SCREENING FOR DEPRESSION] (162813)

121 Clinical Trials/ (105129)

122 (randomi#ation? or randomi#ed or randomly or RCT$1 or placebo*).tw. (2529583)

123 ((singl* or doubl* or trebl* or tripl*) adj (mask* or blind* or dumm*)).tw. (440841)

124 trial.ti. (554231)

125 or/121-124 (2819283)

126 120 and 125 [RCTs - SCREENING FOR DEPRESSION] (16155)

127 Pregnancy/ (1618307)

128 Adolescent Pregnancy/ (20253)

129 exp Obstetrical Complications/ (1546)

130 pregnan*.tw. (1245968)

131 Perinatal Period/ (37680)

132 exp Prenatal Care/ (183998)

133 Postnatal Period/ (39477)

134 (prenatal* or pre-natal* or antenatal* or ante natal* or antepartum or ante partum or perinatal* or peri-natal* or peripartum or peri-partum or postnatal* or post-natal* or postpartum or post partum or puerperal or puerperium).tw. (857630)

135 (maternal* or maternit*).tw. (694817)

136 Expectant Mothers/ (702)

137 (expectant mother* or "new mother" or "new mothers" or "mother-to-be" or "mothers-to-be").tw. (13389)

138 or/127-137 [PREGNANCY/ANTENATAL/POSTNATAL PERIOD] (2774931)

139 126 and 138 [SCREENING FOR DEPRESSION - PREGNANCY/ANTENATAL/POSTNATAL PERIOD] (1244)

140 exp Animals/ not (exp Animals/ and Humans/) (18180610)

141 139 not 140 [ANIMAL-ONLY REMOVED] (867)

142 141 use medall,emczd (688)

143 141 not 142 [PSYCINFO RECORDS] (179)

144 47 or 101 or 143 [ALL DATABASES] (1211)

145 144 use medall [MEDLINE RECORDS] (442)

146 (201810* or 201811* or 201812* or 2019* or 2020*).dt. (2120253)

147 145 and 146 [MEDLINE UPDATE PERIOD] (72)

148 144 use emczd [EMBASE RECORDS] (590)

149 (201810* or 201811* or 201812* or 2019* or 2020*).dc. (3132108)

150 148 and 149 [EMBASE UPDATE PERIOD] (95)

151 144 not (145 or 148) [PSYCINFO RECORDS] (179)

152 (201809* or 201810* or 201811* or 201812* or 2019* or 2020*).up. (35074348)

153 151 and 152 [PSYCINFO UPDATE PERIOD] (26)

154 147 or 150 or 153 [ALL DATABASES - UPDATE PERIOD] (193)

155 remove duplicates from 154 (117) [TOTAL UNIQUE RECORDS]

156 155 use medall [MEDLINE UNIQUE RECORDS - UPDATE PERIOD] (70)

157 155 use emczd [EMBASE UNIQUE RECORDS - UPDATE PERIOD] (43)

158 155 not (156 or 157) [PSYCINFO UNIQUE RECORDS - UPDATE PERIOD] (4)

### **Ovid Multifile (2018 Oct 3)**

Ovid Multifile

2018 Sep 25 (*Updated to 2018 Oct 4*)

Database: Embase Classic+Embase <1947 to 2018 September 24>, Ovid MEDLINE(R) ALL <1946 to September 24, 2018>, PsycINFO <1806 to September Week 3 2018>

Search Strategy:

--------------------------------------------------------------------------------

1 exp Depressive Disorder/ (525659)

2 Depression/ (459247)

3 depress*.tw,kf. (1276494)

4 dysthym*.tw,kf. (10890)

5 blues.tw,kf. (4536)

6 melanchol*.tw,kf. (11670)

7 MDD.tw,kf. (35588)

8 PND.tw,kf. (11472)

9 PPD.tw,kf. (22341)

10 or/1-9 [GENERAL DEPRESSION] (1502164)

11 Mass Screening/ (151048)

12 (screen* or detect*).tw,kf. (6199421)

13 (identif* or recogni*).ti. (804246)

14 ((early or earlier or earliest) adj5 (identif* or recogni*)).tw,kf. (164364)

15 (case finding? or casefinding?).tw,kf. (12220)

16 or/11-15 [GENERAL SCREENING] (6975361)

17 10 and 16 (150596)

18 (controlled clinical trial or randomized controlled trial or pragmatic clinical trial).pt. (556602)

19 clinical trials as topic.sh. (184793)

20 exp Randomized Controlled Trials as Topic/ (269439)

21 (randomi#ation? or randomi#ed or randomly or RCT$1 or placebo*).tw,kf. (2237960)

22 ((singl* or doubl* or trebl* or tripl*) adj (mask* or blind* or dumm*)).tw,kf. (404403)

23 trial.ti. (473473)

24 or/18-23 (2834719)

25 17 and 24 [RCTs - DEPRESSION & SCREENING] (15360)

26 exp Pregnancy/ (1614217)

27 exp Pregnancy Complications/ (532077)

28 Pregnant Women/ (64159)

29 exp Pregnancy Trimesters/ (786958)

30 pregnan*.tw,kf. (1159128)

31 Maternal Health Services/ (13298)

32 Peripartum Period/ (25363)

33 exp Perinatal Care/ (62687)

34 Prenatal Care/ (62600)

35 exp Postpartum Period/ (122793)

36 (prenatal* or pre-natal* or antenatal* or ante natal* or antepartum or ante partum or perinatal* or peri-natal* or peripartum or peri-partum or postnatal* or post-natal* or postpartum or post partum or puerperal or puerperium).tw,kf. (786024)

37 Maternal Health/ (14086)

38 (maternal* or maternit*).tw,kf. (629151)

39 (expectant mother* or "new mother" or "new mothers" or "mother-to-be" or "mothers-to-be").tw,kf. (12000)

40 or/26-39 [PREGNANCY/ANTENATAL/POSTNATAL PERIOD] (2662805)

41 25 and 40 [RCTs - DEPRESSION & SCREENING - PREGNANCY/ANTENATAL/POSTNATAL PERIOD] (1136)

42 exp Animals/ not (exp Animals/ and Humans/) (17261236)

43 41 not 42 [ANIMAL-ONLY REMOVED] (735)

44 (comment or editorial or news or newspaper article).pt. (1833084)

45 (letter not (letter and randomized controlled trial)).pt. (2027319)

46 43 not (44 or 45) [OPINION PIECES REMOVED] (733)

47 46 use medall [MEDLINE RECORDS] (366)

48 exp Depressive Disorder/ (525659)

49 depress*.tw,kw. (1284029)

50 dysthym*.tw,kw. (10981)

51 blues.tw,kw. (4553)

52 melanchol*.tw,kw. (11757)

53 MDD.tw,kw. (35725)

54 PND.tw,kw. (11481)

55 PPD.tw,kw. (22438)

56 or/48-55 [GENERAL DEPRESSION] (1485267)

57 screening/ (283438)

58 mass screening/ (151048)

59 screening test/ (70964)

60 (screen* or detect*).tw,kw. (6209418)

61 (identif* or recogni*).ti. (804246)

62 ((early or earlier or earliest) adj5 (identif* or recogni*)).tw,kw. (164481)

63 (case finding? or casefinding?).tw,kw. (12344)

64 or/57-63 [GENERAL SCREENING] (7012951)

65 depression assessment/ (1237)

66 56 and 64 (151820)

67 65 or 66 [DEPRESSION & SCREENING/ASSESSMENT] (152799)

68 randomized controlled trial/ or controlled clinical trial/ (1253703)

69 exp "clinical trial (topic)"/ (273834)

70 (randomi#ation? or randomi#ed or randomly or RCT$1 or placebo*).tw,kw. (2240072)

71 ((singl* or doubl* or trebl* or tripl*) adj (mask* or blind* or dumm*)).tw,kw. (404551)

72 trial.ti. (473473)

73 or/68-72 (3003708)

74 67 and 73 [RCTs - DEPRESSION & SCREENING/ASSESSMENT] (16656)

75 exp pregnancy/ (1614217)

76 exp pregnancy disorder/ (560149)

77 exp named groups by pregnancy/ (95160)

78 pregnan*.tw,kw. (1168162)

79 exp maternal care/ (41293)

80 perinatal care/ (17354)

81 prenatal care/ (62600)

82 postnatal care/ (11665)

83 perinatal period/ (33799)

84 prenatal period/ (9816)

85 puerperium/ (63000)

86 (prenatal* or pre-natal* or antenatal* or ante natal* or antepartum or ante partum or perinatal* or peri-natal* or peripartum or peri-partum or postnatal* or post-natal* or postpartum or post partum or puerperal or puerperium).tw,kw. (791262)

87 (maternal* or maternit*).tw,kw. (633557)

88 expectant mother/ (1188)

89 (expectant mother* or "new mother" or "new mothers" or "mother-to-be" or "mothers-to-be").tw,kw. (12003)

90 or/75-89 [PREGNANCY/ANTENATAL/POSTNATAL PERIOD] (2761644)

91 74 and 90 [RCTs - DEPRESSION & SCREENING/ASSESSMENT - PREGNANCY/ANTENATAL/POSTNATAL PERIOD] (1232)

92 exp animal experimentation/ or exp animal model/ or exp animal experiment/ or nonhuman/ or exp vertebrate/ (47851101)

93 exp human/ or exp human experimentation/ or exp human experiment/ (37400820)

94 92 not 93 (10451986)

95 91 not 94 [ANIMAL-ONLY REMOVED] (1143)

96 editorial.pt. (1044960)

97 letter.pt. not (letter.pt. and randomized controlled trial/) (2022423)

98 95 not (96 or 97) [OPINION PIECES REMOVED] (1141)

99 conference abstract.pt. (3151029)

100 98 not 99 [CONFERENCE ABSTRACTS REMOVED] (995)

101 100 use emczd [EMBASE RECORDS] (492)

102 "Depression (Emotion)"/ (128391)

103 exp Major Depression/ (174970)

104 depress*.tw. (1271702)

105 dysthym*.tw. (10872)

106 blues.tw. (4529)

107 melanchol*.tw. (11631)

108 MDD.tw. (35498)

109 PND.tw. (11408)

110 PPD.tw. (22291)

111 or/102-110 [GENERAL DEPRESSION] (1346393)

112 Screening/ (283438)

113 exp Screening Tests/ (24701)

114 exp Health Screening/ (229651)

115 (screen* or detect*).tw. (6193289)

116 (identif* or recogni*).ti. (804246)

117 ((early or earlier or earliest) adj5 (identif* or recogni*)).tw. (164292)

118 (case finding? or casefinding?).tw. (12169)

119 or/112-118 [GENERAL SCREENING] (7046364)

120 111 and 119 [SCREENING FOR DEPRESSION] (142325)

121 Clinical Trials/ (92465)

122 (randomi#ation? or randomi#ed or randomly or RCT$1 or placebo*).tw. (2236103)

123 ((singl* or doubl* or trebl* or tripl*) adj (mask* or blind* or dumm*)).tw. (404314)

124 trial.ti. (473473)

125 or/121-124 (2496825)

126 120 and 125 [RCTs - SCREENING FOR DEPRESSION] (13745)

127 Pregnancy/ (1533135)

128 Adolescent Pregnancy/ (19199)

129 exp Obstetrical Complications/ (1311)

130 pregnan*.tw. (1140451)

131 Perinatal Period/ (33799)

132 exp Prenatal Care/ (168769)

133 Postnatal Period/ (35529)

134 (prenatal* or pre-natal* or antenatal* or ante natal* or antepartum or ante partum or perinatal* or peri-natal* or peripartum or peri-partum or postnatal* or post-natal* or postpartum or post partum or puerperal or puerperium).tw. (781733)

135 (maternal* or maternit*).tw. (623551)

136 Expectant Mothers/ (616)

137 (expectant mother* or "new mother" or "new mothers" or "mother-to-be" or "mothers-to-be").tw. (11995)

138 or/127-137 [PREGNANCY/ANTENATAL/POSTNATAL PERIOD] (2580563)

139 126 and 138 [SCREENING FOR DEPRESSION - PREGNANCY/ANTENATAL/POSTNATAL PERIOD] (1035)

140 exp Animals/ not (exp Animals/ and Humans/) (17261236)

141 139 not 140 [ANIMAL-ONLY REMOVED] (684)

142 141 use medall,emczd (530)

143 141 not 142 [PSYCINFO RECORDS] (154)

144 47 or 101 or 143 [ALL DATABASES] (1012)

145 remove duplicates from 144 (597)

146 145 use medall (364)

147 145 use emczd (193)

148 145 not (146 or 147) (40)

### **Cochrane Library (2020 May 11)**


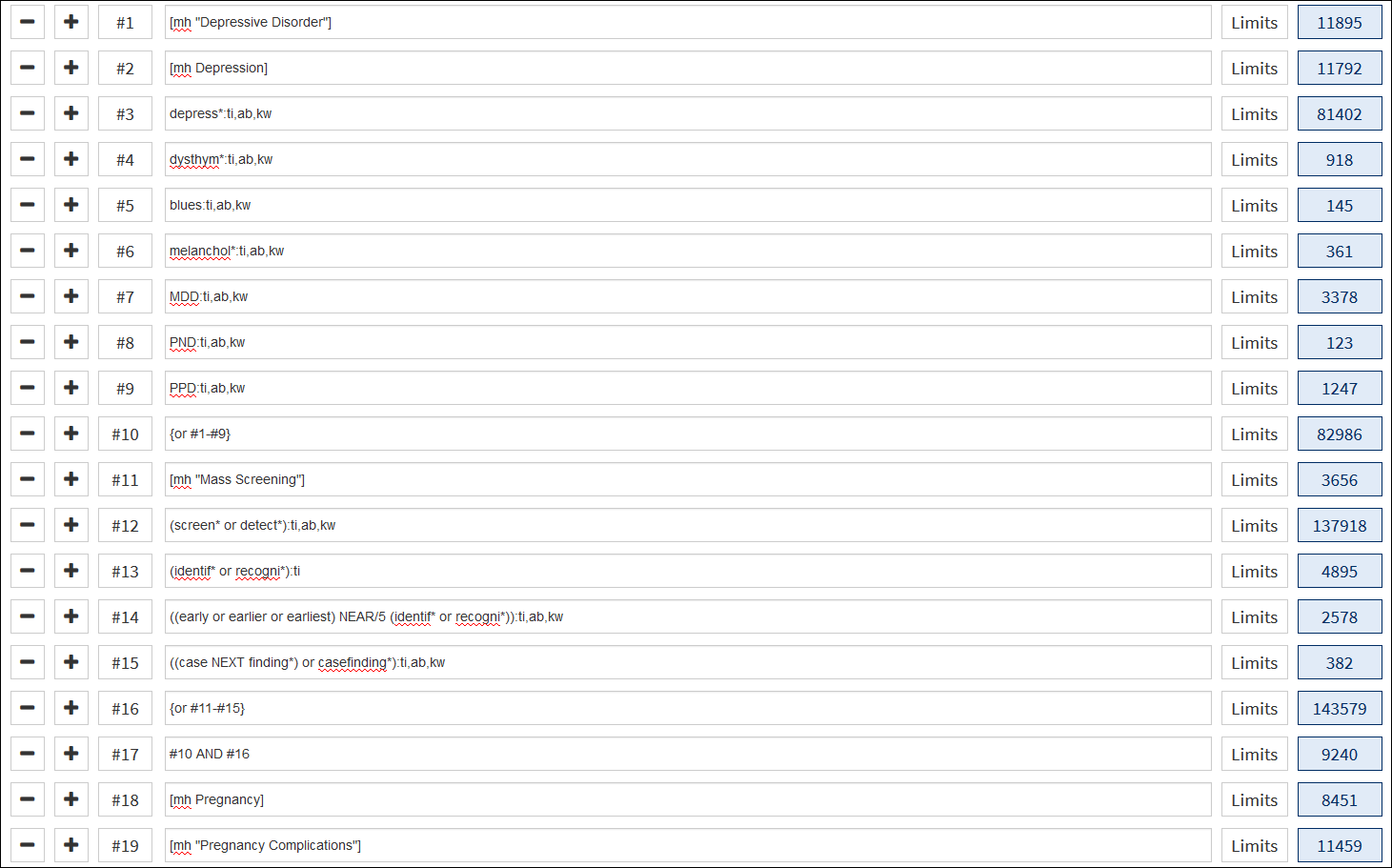


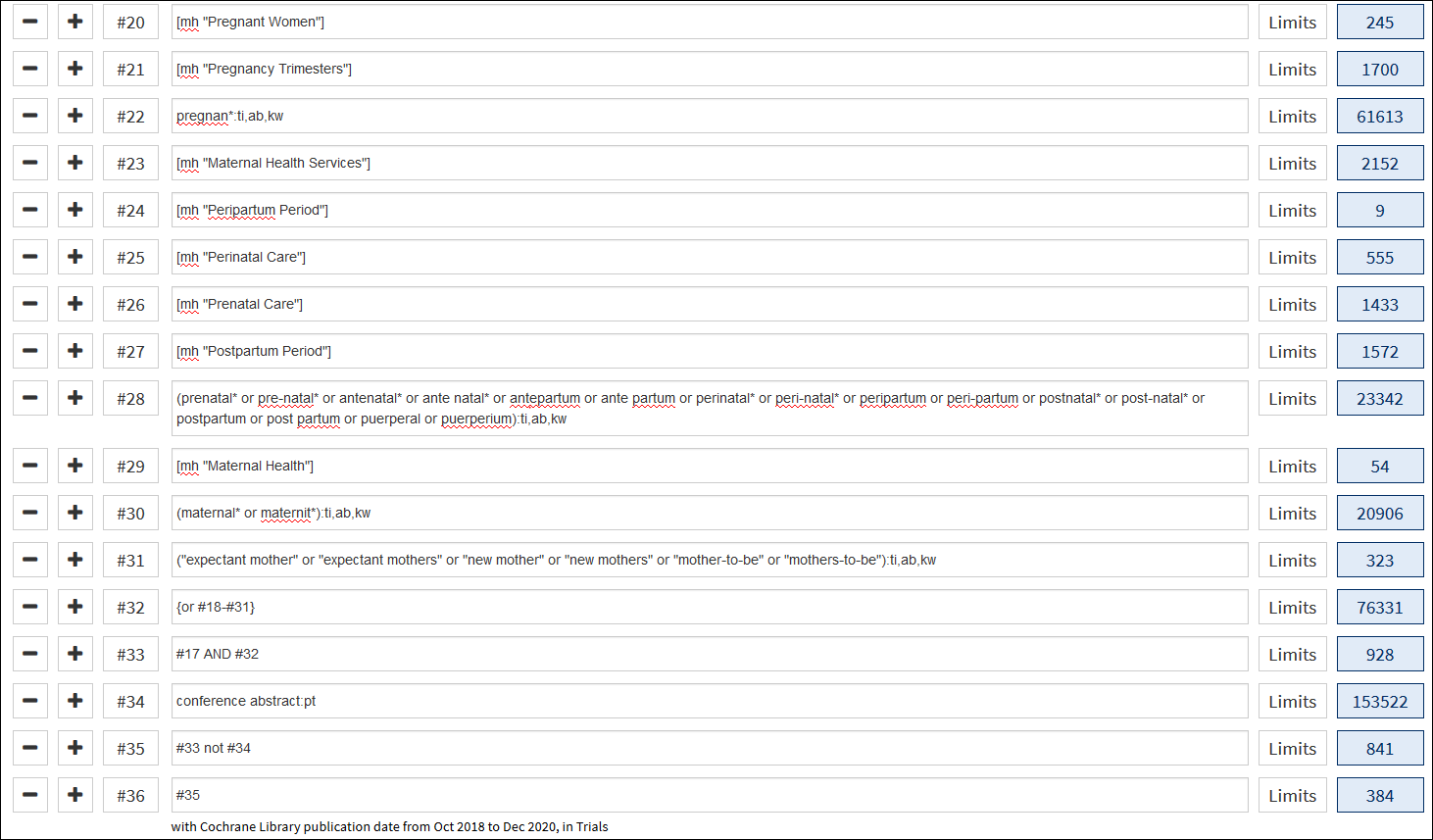


### **Cochrane Library (2018 Oct 4)**

Search Name: CTFPHC - Depression - Screening - Pregnancy & Postpartum

Date Run: 04/10/2018 18:26:48

ID Search Hits

#1 MeSH descriptor: ["Depressive Disorder"] explode all trees 10295

#2 MeSH descriptor: [Depression] explode all trees 9672

#3 depress*:ti,ab,kw 61914

#4 dysthym*:ti,ab,kw 755

#5 blues:ti,ab,kw 97

#6 melanchol*:ti,ab,kw 320

#7 MDD:ti,ab,kw 2560

#8 PND:ti,ab,kw 83

#9 PPD:ti,ab,kw 877

#10 {or #1-#9} 63086

#11 MeSH descriptor: ["Mass Screening"] explode all trees 2885

#12 (screen* or detect*):ti,ab,kw 104808

#13 (identif* or recogni*):ti 4474

#14 ((early or earlier or earliest) near/5 (identif* or recogni*)):ti,ab,kw 2161

#15 ((case next finding*) or casefinding*):ti,ab,kw 297

#16 {or #11-#15} 109756

#17 #10 and #16 6411

#18 MeSH descriptor: [Pregnancy] explode all trees 6926

#19 MeSH descriptor: ["Pregnancy Complications"] explode all trees 9894

#20 MeSH descriptor: ["Pregnant Women"] explode all trees 182

#21 MeSH descriptor: ["Pregnancy Trimesters"] explode all trees 1618

#22 pregnan*:ti,ab,kw 40240

#23 MeSH descriptor: ["Maternal Health Services"] explode all trees 1898

#24 MeSH descriptor: ["Peripartum Period"] explode all trees 7

#25 MeSH descriptor: ["Perinatal Care"] explode all trees 493

#26 MeSH descriptor: ["Prenatal Care"] explode all trees 1268

#27 MeSH descriptor: ["Postpartum Period"] explode all trees 1414

#28 (prenatal* or pre-natal* or antenatal* or ante natal* or antepartum or ante partum or perinatal* or peri-natal* or peripartum or peri-partum or postnatal* or post-natal* or postpartum or post partum or puerperal or puerperium):ti,ab,kw 17540

#29 MeSH descriptor: ["'Maternal Health"] explode all trees 37

#30 (maternal* or maternit*):ti,ab,kw 16029

#31 ((expectant next mother*) or "new mother" or "new mothers" or "mother-to-be" or "mothers-to-be"):ti,ab,kw 1133

#32 {or #18-#31} 51831

#33 #17 and #32 566

#34 conference abstract:pt 121946

#35 #33 NOT #34 with Cochrane Library publication date between Jan 2012 and Jan 2018, in Trials 172

Trials: 172

### **CINAHL (2020 May 11)**

| # | Query | Limiters/Expanders | Results |
| --- | --- | --- | --- |
| S44 | S40 OR S41 | Limiters - Published Date: 20180101-20201231; Exclude MEDLINE records  Expanders - Apply related words  Search modes - Boolean/Phrase | 21 |
| S43 | S40 OR S41 | Limiters - Exclude MEDLINE records  Expanders - Apply related words  Search modes - Boolean/Phrase | 97 |
| S42 | S40 OR S41 | Expanders - Apply related words  Search modes - Boolean/Phrase | 264 |
| S41 | S37 not S39 | Expanders - Apply related words  Search modes - Boolean/Phrase | 244 |
| S40 | S37 not S38 | Expanders - Apply related words  Search modes - Boolean/Phrase | 259 |
| S39 | (MH "Child+") NOT ( (MH "Child+" AND MH "Adolescence+") ) | Expanders - Apply related words  Search modes - Boolean/Phrase | 466,209 |
| S38 | (MH "Child+") NOT ( (MH "Adult+" AND MH "Child+") ) | Expanders - Apply related words  Search modes - Boolean/Phrase | 529,599 |
| S37 | S35 NOT S36 | Expanders - Apply related words  Search modes - Boolean/Phrase | 288 |
| S36 | (MH "Male") NOT ( (MH "Female" AND MH "Male") ) | Expanders - Apply related words  Search modes - Boolean/Phrase | 360,731 |
| S35 | S23 AND S34 | Expanders - Apply related words  Search modes - Boolean/Phrase | 288 |
| S34 | S24 OR S25 OR S26 OR S27 OR S28 OR S29 OR S30 OR S31 OR S32 OR S33 | Expanders - Apply related words  Search modes - Boolean/Phrase | 360,550 |
| S33 | TI ( "expectant mother" or "expectant mothers" or "new mother" or "new mothers" or "mother-to-be" or "mothers-to-be" ) OR AB ( "expectant mother" or "expectant mothers" or "new mother" or "new mothers" or "mother-to-be" or "mothers-to-be" ) | Expanders - Apply related words  Search modes - Boolean/Phrase | 82,346 |
| S32 | TI ( maternal* or maternit* ) OR AB ( maternal* or maternit* ) | Expanders - Apply related words  Search modes - Boolean/Phrase | 91,833 |
| S31 | TI ( prenatal* or pre-natal* or antenatal* or ante natal* or antepartum or ante partum or perinatal* or peri-natal* or peripartum or peri-partum or postnatal* or post-natal* or postpartum or post partum or puerperal or puerperium ) OR AB ( prenatal* or pre-natal* or antenatal* or ante natal* or antepartum or ante partum or perinatal* or peri-natal* or peripartum or peri-partum or postnatal* or post-natal* or postpartum or post partum or puerperal or puerperium ) | Expanders - Apply related words  Search modes - Boolean/Phrase | 95,193 |
| S30 | (MH "Postnatal Period+") | Expanders - Apply related words  Search modes - Boolean/Phrase | 14,043 |
| S29 | (MH "Perinatal Care") OR (MH "Postnatal Care+") OR (MH "Prenatal Care") | Expanders - Apply related words  Search modes - Boolean/Phrase | 26,017 |
| S28 | (MH "Maternal Health Services") | Expanders - Apply related words  Search modes - Boolean/Phrase | 9,884 |
| S27 | TI pregnan* OR AB pregnan* | Expanders - Apply related words  Search modes - Boolean/Phrase | 139,256 |
| S26 | (MH "Pregnancy Trimesters") | Expanders - Apply related words  Search modes - Boolean/Phrase | 1,123 |
| S25 | (MH "Expectant Mothers") | Expanders - Apply related words  Search modes - Boolean/Phrase | 8,213 |
| S24 | (MH "Pregnancy+") OR (MH "Pregnancy, Unwanted") OR (MH "Pregnancy, Multiple") | Expanders - Apply related words  Search modes - Boolean/Phrase | 218,780 |
| S23 | S16 AND S22 | Expanders - Apply related words  Search modes - Boolean/Phrase | 2,585 |
| S22 | S17 OR S18 OR S19 OR S20 OR S21 | Expanders - Apply related words  Search modes - Boolean/Phrase | 399,870 |
| S21 | TI trial | Expanders - Apply related words  Search modes - Boolean/Phrase | 111,730 |
| S20 | TI ( ((singl* or doubl* or trebl* or tripl*) N1 (mask* or blind* or dumm*)) ) OR AB ( ((singl* or doubl* or trebl* or tripl*) N1 (mask* or blind* or dumm*)) ) | Expanders - Apply related words  Search modes - Boolean/Phrase | 50,868 |
| S19 | TI ( randomi?ed or randomly or RCT or RCTs or placebo* ) OR AB ( randomi?ed or randomly or RCT or RCTs or placebo* ) | Expanders - Apply related words  Search modes - Boolean/Phrase | 336,728 |
| S18 | (MH "Double-Blind Studies") OR (MH "Single-Blind Studies") OR (MH "Triple-Blind Studies") | Expanders - Apply related words  Search modes - Boolean/Phrase | 64,816 |
| S17 | (MH "Randomized Controlled Trials") | Expanders - Apply related words  Search modes - Boolean/Phrase | 118,438 |
| S16 | S9 AND S15 | Expanders - Apply related words  Search modes - Boolean/Phrase | 22,741 |
| S15 | S10 OR S11 OR S12 OR S13 OR S14 | Expanders - Apply related words  Search modes - Boolean/Phrase | 468,145 |
| S14 | TI ( "case finding" or "case findings" or casefinding or casefindings ) OR AB ( "case finding" or "case findings" or casefinding or casefindings ) | Expanders - Apply related words  Search modes - Boolean/Phrase | 1,588 |
| S13 | TI ( ((early or earlier or earliest) N5 (identif* or recogni*)) ) OR AB ( ((early or earlier or earliest) N5 (identif* or recogni*)) ) | Expanders - Apply related words  Search modes - Boolean/Phrase | 22,820 |
| S12 | TI (identif* or recogni*) | Expanders - Apply related words  Search modes - Boolean/Phrase | 62,534 |
| S11 | TI ( (screen* or detect*) ) OR AB ( (screen* or detect*) ) | Expanders - Apply related words  Search modes - Boolean/Phrase | 382,286 |
| S10 | (MH "Health Screening") | Expanders - Apply related words  Search modes - Boolean/Phrase | 48,758 |
| S9 | S1 OR S2 OR S3 OR S4 OR S5 OR S6 OR S7 OR S8 | Expanders - Apply related words  Search modes - Boolean/Phrase | 197,301 |
| S8 | TI PPD OR AB PPD | Expanders - Apply related words  Search modes - Boolean/Phrase | 1,861 |
| S7 | TI PND OR AB PND | Expanders - Apply related words  Search modes - Boolean/Phrase | 697 |
| S6 | TI MDD OR AB MDD | Expanders - Apply related words  Search modes - Boolean/Phrase | 3,995 |
| S5 | TI melanchol* OR AB melanchol* | Expanders - Apply related words  Search modes - Boolean/Phrase | 499 |
| S4 | TI blues OR AB blues | Expanders - Apply related words  Search modes - Boolean/Phrase | 9,665 |
| S3 | TI dysthym* OR AB dysthym* | Expanders - Apply related words  Search modes - Boolean/Phrase | 838 |
| S2 | TI depress* OR AB depress* | Expanders - Apply related words  Search modes - Boolean/Phrase | 156,217 |
| S1 | (MH "Depression+") | Expanders - Apply related words  Search modes - Boolean/Phrase | 120,272 |

### **CINAHL (2018 Oct 4)**

| # | Query | Limiters/Expanders | Results |
| --- | --- | --- | --- |
| S43 | S40 OR S41 | Limiters - Exclude MEDLINE records  Expanders - Apply related words  Search modes - Boolean/Phrase | 29 |
| S42 | S40 OR S41 | Expanders - Apply related words  Search modes - Boolean/Phrase | 116 |
| S41 | S37 not S39 | Expanders - Apply related words  Search modes - Boolean/Phrase | 104 |
| S40 | S37 not S38 | Expanders - Apply related words  Search modes - Boolean/Phrase | 113 |
| S39 | (MH "Child+") NOT ( (MH "Child+" AND MH "Adolescence+") ) | Expanders - Apply related words  Search modes - Boolean/Phrase | 256,396 |
| S38 | (MH "Child+") NOT ( (MH "Adult+" AND MH "Child+") ) | Expanders - Apply related words  Search modes - Boolean/Phrase | 285,658 |
| S37 | S35 NOT S36 | Expanders - Apply related words  Search modes - Boolean/Phrase | 120 |
| S36 | (MH "Male") NOT ( (MH "Female" AND MH "Male") ) | Expanders - Apply related words  Search modes - Boolean/Phrase | 168,288 |
| S35 | S23 AND S34 | Expanders - Apply related words  Search modes - Boolean/Phrase | 120 |
| S34 | S24 OR S25 OR S26 OR S27 OR S28 OR S29 OR S30 OR S31 OR S32 OR S33 | Expanders - Apply related words  Search modes - Boolean/Phrase | 184,966 |
| S33 | TI ( "expectant mother" or "expectant mothers" or "new mother" or "new mothers" or "mother-to-be" or "mothers-to-be" ) OR AB ( "expectant mother" or "expectant mothers" or "new mother" or "new mothers" or "mother-to-be" or "mothers-to-be" ) | Expanders - Apply related words  Search modes - Boolean/Phrase | 40,200 |
| S32 | TI ( maternal* or maternit* ) OR AB ( maternal* or maternit* ) | Expanders - Apply related words  Search modes - Boolean/Phrase | 42,155 |
| S31 | TI ( prenatal* or pre-natal* or antenatal* or ante natal* or antepartum or ante partum or perinatal* or peri-natal* or peripartum or peri-partum or postnatal* or post-natal* or postpartum or post partum or puerperal or puerperium ) OR AB ( prenatal* or pre-natal* or antenatal* or ante natal* or antepartum or ante partum or perinatal* or peri-natal* or peripartum or peri-partum or postnatal* or post-natal* or postpartum or post partum or puerperal or puerperium ) | Expanders - Apply related words  Search modes - Boolean/Phrase | 44,338 |
| S30 | (MH "Postnatal Period+") | Expanders - Apply related words  Search modes - Boolean/Phrase | 7,162 |
| S29 | (MH "Perinatal Care") OR (MH "Postnatal Care+") OR (MH "Prenatal Care") | Expanders - Apply related words  Search modes - Boolean/Phrase | 15,138 |
| S28 | (MH "Maternal Health Services") | Expanders - Apply related words  Search modes - Boolean/Phrase | 5,658 |
| S27 | TI pregnan* OR AB pregnan* | Expanders - Apply related words  Search modes - Boolean/Phrase | 63,749 |
| S26 | (MH "Pregnancy Trimesters") | Expanders - Apply related words  Search modes - Boolean/Phrase | 530 |
| S25 | (MH "Expectant Mothers") | Expanders - Apply related words  Search modes - Boolean/Phrase | 3,693 |
| S24 | (MH "Pregnancy+") OR (MH "Pregnancy, Unwanted") OR (MH "Pregnancy, Multiple") | Expanders - Apply related words  Search modes - Boolean/Phrase | 120,652 |
| S23 | S16 AND S22 | Expanders - Apply related words  Search modes - Boolean/Phrase | 1,128 |
| S22 | S17 OR S18 OR S19 OR S20 OR S21 | Expanders - Apply related words  Search modes - Boolean/Phrase | 186,312 |
| S21 | TI trial | Expanders - Apply related words  Search modes - Boolean/Phrase | 49,258 |
| S20 | TI ( ((singl* or doubl* or trebl* or tripl*) N1 (mask* or blind* or dumm*)) ) OR AB ( ((singl* or doubl* or trebl* or tripl*) N1 (mask* or blind* or dumm*)) ) | Expanders - Apply related words  Search modes - Boolean/Phrase | 23,339 |
| S19 | TI ( randomi?ed or randomly or RCT or RCTs or placebo* ) OR AB ( randomi?ed or randomly or RCT or RCTs or placebo* ) | Expanders - Apply related words  Search modes - Boolean/Phrase | 155,628 |
| S18 | (MH "Double-Blind Studies") OR (MH "Single-Blind Studies") OR (MH "Triple-Blind Studies") | Expanders - Apply related words  Search modes - Boolean/Phrase | 33,052 |
| S17 | (MH "Randomized Controlled Trials") | Expanders - Apply related words  Search modes - Boolean/Phrase | 43,953 |
| S16 | S9 AND S15 | Expanders - Apply related words  Search modes - Boolean/Phrase | 10,536 |
| S15 | S10 OR S11 OR S12 OR S13 OR S14 | Expanders - Apply related words  Search modes - Boolean/Phrase | 207,189 |
| S14 | TI ( "case finding" or "case findings" or casefinding or casefindings ) OR AB ( "case finding" or "case findings" or casefinding or casefindings ) | Expanders - Apply related words  Search modes - Boolean/Phrase | 730 |
| S13 | TI ( ((early or earlier or earliest) N5 (identif* or recogni*)) ) OR AB ( ((early or earlier or earliest) N5 (identif* or recogni*)) ) | Expanders - Apply related words  Search modes - Boolean/Phrase | 10,843 |
| S12 | TI (identif* or recogni*) | Expanders - Apply related words  Search modes - Boolean/Phrase | 31,342 |
| S11 | TI ( (screen* or detect*) ) OR AB ( (screen* or detect*) ) | Expanders - Apply related words  Search modes - Boolean/Phrase | 162,964 |
| S10 | (MH "Health Screening") | Expanders - Apply related words  Search modes - Boolean/Phrase | 25,530 |
| S9 | S1 OR S2 OR S3 OR S4 OR S5 OR S6 OR S7 OR S8 | Expanders - Apply related words  Search modes - Boolean/Phrase | 93,973 |
| S8 | TI PPD OR AB PPD | Expanders - Apply related words  Search modes - Boolean/Phrase | 818 |
| S7 | TI PND OR AB PND | Expanders - Apply related words  Search modes - Boolean/Phrase | 291 |
| S6 | TI MDD OR AB MDD | Expanders - Apply related words  Search modes - Boolean/Phrase | 982 |
| S5 | TI melanchol* OR AB melanchol* | Expanders - Apply related words  Search modes - Boolean/Phrase | 196 |
| S4 | TI blues OR AB blues | Expanders - Apply related words  Search modes - Boolean/Phrase | 4,232 |
| S3 | TI dysthym* OR AB dysthym* | Expanders - Apply related words  Search modes - Boolean/Phrase | 415 |
| S2 | TI depress* OR AB depress* | Expanders - Apply related words  Search modes - Boolean/Phrase | 72,017 |
| S1 | (MH "Depression+") | Expanders - Apply related words  Search modes - Boolean/Phrase | 60,285 |

## Additional file 5. Adult population evidence sets

### Additional file 5.1. Adult study characteristics table

| **Author Year, Study design, Funding** | **Participants, Location** | **Intervention / Comparison** | **Outcomes** |
| --- | --- | --- | --- |
| Kronish 2019, USA  **Study design**: Multicenter randomized clinical trial  **Funding**: National Heart, Lung, and Blood Institute | Participants identified via EMR aged 21 years or older with documented ACS within 2 to 12 months of enrollment.  Participants excluded if they: (1) were currently receiving treatment for depression or had a prior history of depression, (2) had prior or current history of bipolar disorder, suicidal risk, or psychosis, (3) were currently pregnant, (4) had current substance abuse, dementia, severe arthritis or rheumatologic illness, advanced heart failure, advanced liver-, lung-disease needing oxygen at home, advanced HIV/AIDS, advanced cancer, or (5) life expectancy less than 1 year.  **Location**: Four health care systems located in Minnesota, North Carolina, Oregon, and New York states in the USA | **Intervention**: Participants assigned to the screen and notify group (n=501) were systematically screened for depression using the PHQ-8. Cardiologists and/or primary care providers were notified of clinically significant depressive symptoms (PHQ-8 score ≥10) via letter. Subsequent depression treatment decisions were made by the participants’ treating clinician and participants were responsible for out-of-pocket costs of treatment.  A second intervention group was included in the study who were assigned to the screen, notify, and treat group (n=499). However, this group was not included in this systematic review because of treatment intervention (i.e., did not meet the inclusion criteria).  **Comparison**: Participants (n=500) received usual care from their treating clinician and were able to seek mental health screening and/or depression treatment at their own expense. | **Depression:**   - measured with the CESD-10. Scores 0-30. A higher score suggesting a greater severity of depressive symptoms. - measured with PHQ-8. Score ≥10 defined as depression (baseline data for screening arm not recorded).   **Depression-free days**: converted from CESD-10 scores. Ranged from 0 depression day (CESD score <4) to 1 depression day (CESD ≥10).  **Health-related quality of life:**   - *Quality-of-life utility scores*: estimated using SF-6 (scores derived from SF-12 responses). Overall assessment of well-being on a scale from 0 (death) to 1 (perfect health). - *Change in QALYs from baseline to 18 months*: estimated using SF-6 (scores derived from SF-12 responses).   **Harms of depression screening**: assessed through patient interview. Potential adverse effects from antidepressant medication use (i.e., appetite problems, sleep problems, gastrointestinal upset, and bleeding).  **Mortality:** assessed by surveying patient surrogates and through review of the electronic medical record.  **Follow-up:** 6, 12, and 18 months. PHQ-8 only at 18 months. |
| Leung 2011, Hong Kong, China  **Study design**: Randomized controlled trial  **Funding**: Not reported. Sponsors and collaborators were Chinese University of Hong Kong, Hong Kong Department of Health, and The University of Hong Kong | 462 Chinese mothers with 2-month-old babies visiting Maternal and Child Health Centres in Hong Kong.  Those who participated in other postnatal depression screening programmes or were receiving psychiatric treatment were excluded.  **Location**: Maternal and Child Health Centres in Hong Kong | **Intervention**: n=231 participants were allocated to the intervention group. The Edinburgh Postnatal Depression Scale was used to identify participants with postnatal depression. Women were also clinically assessed. The EPDS has 10 questions, and scores range from 0-30 (worse). Those with scores above the cut-off (≥10) or suicidal ideation were offered non-directive counselling by MCH nurses or management by the community psychiatric team as appropriate. The Chinese version of the EPDS was validated with Hong Kong women at 6 weeks postnatal, against the structured clinical interview for DSM-III-R.  Treatment: 55 received treatment (46 received counselling by MCH nurses, of whom eight received additional psychiatric nurse counselling whereas three received psychiatric nurse counselling and treatment by a psychiatrist; another 8 received psychiatric nurse counselling initially, with one of them receiving further treatment by a psychiatrist; and 1 participant received treatment by a psychiatrist only).  **Comparison: n=**231 participants were allocated to the control group: General Self-Efficacy Scale plus usual practice where nurses carried out clinical assessment. Mothers deemed to require further management were offered non-directive counselling or psychiatric referral.  Treatment: 11 received treatment (10 received MCH nurse counselling initially; four of whom received further psychiatric nurse counselling, with one of these having received additional psychiatric treatment; 1 participant received counselling by a psychiatric nurse only). | **Depression:** measured with the EPDS (score ≥10 (score 0-30)). A higher score represents a worse rating.  **Adverse events:** unclear how this was collected/reported.  **General health:** measured with GHQ (score 0-12). A higher score represents a worse rating.  **Follow-up:** 2 months postpartum (baseline), 6 months postpartum (i.e., 4 months after randomization), 18 months postpartum (i.e., 16 months after randomization).  *At 18 months postpartum, the control group had received the EPDS at 6-months postpartum and offered treatment/follow-up services for those who scored ≥10, thereby removing the screened versus not screened comparison.* |
| Mallen 2017, UK  **Study design**: Pragmatic cluster randomized trial (clinical practices were randomized)  **Funding**: National  Institute for Health Research Programme Grant, and by Christian Mallen's  Arthritis Research UK Clinician Scientist Award | Participants aged 45 years or older who consulted for osteoarthritis (OA) and an OA Read code was recorded in their electronic general practitioner (GP) records. Consultations relating to a clinical diagnosis of OA (index consultation) could be first, new episode, or ongoing consultations.  Patients were excluded by their GP at the point of template completion if they were under active care for, or having a diagnosis of, depression and/or an anxiety disorder in the past 12 months.  **Location**: General practices in the West Midlands North region of England | **Intervention**: Point-of-care anxiety and depression screening questions posed by the GP, prompted by electronic template. The template consisted of five questions: a two-item ultra-brief depression tool (PHQ-2), a two-item ultra-brief anxiety assessment tool (GAD-2), and an item on current pain intensity rated from 0-10. The template then signposted and encouraged GPs to follow NICE clinical guidelines on the management of OA, depression in adults with a chronic physical health problem, and anxiety. At a post-randomization meeting approximately 1 week prior to the template being activated in the practice, a GP research facilitator (GPRF) explained and discussed the study procedures with GPs and practice staff. Brief face-to-face training was provided, explaining NICE-recommended evidence-based approaches to managing comorbid anxiety and depression, and hard copies of the screening questions and quick reference versions of the guidelines were placed in all consulting rooms in the intervention practices. Reminder posters were placed in all consulting rooms to act as further prompts to the study. n=501.  **Comparison**: Point-of-care pain intensity assessment by the GP, also prompted by the electronic template but containing only the item on current pain intensity. At the post-randomization meeting with the GPRF, GPs were advised to follow their usual approach for responding to a patient's pain intensity rating. No additional information or signposting on management was provided. n=911. | **Depression**: measured with the PHQ-8. Score 0-24. A higher score represents a worse rating.  **Health-related quality of life**: measured by general health status with the SF-MCS and SF-PCS. Score 0-100. Lower scores represent a worse rating.  **Follow-up**: post-consultation, 3, 6 and 12 months |

**ACS**: acute coronary syndrome; **CESD-10**: 10-Item Center for Epidemiologic Studies Depression Scale; **DSM:** Diagnostic and Statistical Manual of Mental Disorders, Third edition-Revised; **EMR**: electronic medical record; **EPDS**: Edinburgh Postpartum Depression Score; **GAD-2**: ultra-brief anxiety assessment tool; **GHQ**: General Health Questionnaire; **GP**: General practitioner; **GPRF**: general practitioner research facilitator; **kg**: kilograms; **MCH**: Maternal and Child Health Centres; **OA**: osteoarthritis; **PHQ**: Patient Health Questionnaire; **QALYs**: quality-adjusted life-years; **SF-MCS**: Medical Outcomes Study Short Form 12-Mental Component Score; **SF-6D**: Short-Form Six-Dimension; **SF-12**: 12-Item Short Form Health Survey (version 2); **SF-PCS**: Medical Outcomes Study Short Form 12-Physical Component Score

### Additional file 5.2. Adult results table (continuous data)

| **Author Year** | **Outcome** | **Results: Mean (SD)** | | | | | **Absolute effect**  **SMD (95% CI)** | **Certainty** |
| --- | --- | --- | --- | --- | --- | --- | --- | --- |
|  |  | **Intervention** | **Control** | **MD (95% CI)** | **Adjusted MD (95% CI)** | **p-value** |  |  |
| **Symptoms of depression or diagnosis of MDD outcomes** | | | | | | | | |
| **Symptoms of depression** (measured by the CESD-10. Scores range from 0 to 30. Higher scores suggesting a greater severity of depressive symptoms.) | | | | | | | | |
| Kronish 2019 | Baseline | 4.8 (4.9) | 4.7 (4.6) | 0.10  (-0.49 to 0.69) | - | - | SMD **0.02** **higher**  (0.10 lower to 0.15 higher) | ⨁⨁⨁◯  MODERATE |
|  | 6 months | 6.1 (5.8) | 6.0 (5.3) | 0.10  (-0.59 to 0.79) | - | - | SMD **0.02** **higher**  (0.11 lower to 0.14 higher) | ⨁⨁⨁◯  MODERATE |
|  | 12 months | 5.9 (5.5) | 6.5 (5.8) | -0.60  (-1.30 to 0.10) | - | - | SMD **0.11** **lower**  (0.23 lower to 0.02 higher) | ⨁⨁⨁◯  MODERATE |
|  | 18 months | 5.3 (5.5) | 5.6 (5.4) | -0.30  (-0.98 to 0.38) | - | - | SMD **0.06** **lower**  (0.18 lower to 0.07 higher) | ⨁⨁⨁◯  MODERATE |
| **Change in depressive symptoms** (change in CESD-10 score from baseline to 18-month follow-up) | | | | | | | | |
| Kronish 2019 | Women | 0.8 (3.4) | 1.1 (3.5) | -0.30  (-1.11 to 0.51) | - | - | SMD **0.09 lower**  (0.32 lower to 0.15 higher) | ⨁⨁◯◯  LOW |
|  | Men | 0.9 (3.2) | 1.2 (3.4) | -0.30  (-0.78 to 0.18) | - | - | SMD **0.09 lower**  (0.24 lower to 0.06 higher) | ⨁⨁⨁◯  MODERATE |
| **Symptoms of depression** (measured by the PHQ-8. Scores range from 0 to 24. Higher scores suggesting a greater severity of depressive symptoms.) | | | | | | | | |
| Kronish 2019 | Baseline | 2.7 (3.5) | NA | Not estimable | - | - | Not estimable | - |
|  | 18 months | 3.6 (4.1) | 3.7 (4.2) | -0.10  (-0.61 to 0.41) | - | - | SMD **0.02** **lower**  (0.15 lower to 0.10 higher) | ⨁⨁⨁◯  MODERATE |
|  | Women (18-mths) | 4.6 (4.8) | 4.5 (4.4) | 0.10  (-0.98 to 1.18) | - | - | SMD **0.02** **higher**  (0.21 lower to 0.26 higher) | ⨁⨁◯◯  LOW |
|  | Men (18-mths) | 3.3 (4.0) | 3.6 (4.3) | -0.30  (-0.91 to 0.31) | - | - | SMD **0.07** **lower**  (0.22 lower to 0.07 higher) | ⨁⨁⨁◯  MODERATE |
| **Symptoms of depression** (measured with the PHQ-8. Scores range from 0 to 24, with higher scores representing a worse rating) | | | | | | | | |
| Mallen 2017 | Post-consultation | 6.4 (6.1) | 6.0 (6.0) | 0.40  (-0.27 to 1.07) | 0.30  (-0.40 to 1.00) **^Ϯ^** | 0.402 | SMD **0.07 higher** (0.04 lower to 0.18 higher) **^ꬹ^** | ⨁◯◯◯ VERY LOW |
|  | 3 months | 6.0 (6.1) | 5.2 (5.7) | 0.80  (0.06 to 1.54) | 0.52  (-0.22 to 1.26) **^Ϯ^** | 0.172 | SMD **0.14 higher**  (0.01 higher to 0.26 higher) **^ꬹ^** | ⨁◯◯◯ VERY LOW |
|  | 6 months | 6.6 (6.3) | 5.3 (5.7) | 1.30  (0.53 to 2.07) | 0.74  (-0.02 to 1.49) **^Ϯ^** | 0.055 | SMD **0.22 higher**  (0.09 higher to 0.35 higher) **^ꬹ^** | ⨁◯◯◯ VERY LOW |
|  | 12 months | 6.0 (6.1) | 5.4 (6.0) | 0.60  (-0.18 to 1.38) | 0.36  (-0.41 to 1.14) **^Ϯ^** | 0.360 | SMD **0.10 higher**  (0.03 lower to 0.23 higher) **^ꬹ^** | ⨁◯◯◯ VERY LOW |
| **Symptoms of depression** (measured with the EPDS. Scores range from 0 to 30, with higher scores representing a worse rating) | | | | | | | | |
| Leung 2011 | Women (6-mths postpartum/4-mths after randomization) | 5.1 (3.6) | 6.5 (4.4) | -1.4  (-0.6 to -2.1) | - | <0.001 | SMD **0.34 lower**  (0.15 lower to 0.52 lower) | ⨁◯◯◯ VERY LOW |
| **Symptoms of depression** (measured with the GHQ-12. Scores range from 0 to 12, with higher scores representing a worse rating) | | | | | | | | |
| Leung 2011 | Women (6-mths postpartum/4-mths after randomization) | 1.1 (1.8) | 1.4 (2.2) | -0.3  (-0.7 to 0.04) | - | 0.084 | SMD **0.16 lower**  (0.35 lower to 0.02 higher) | ⨁◯◯◯ VERY LOW |
| **Depression-free days** (cumulative mean (SD)) (converted CESD-10 score to depression day. Ranged from 0 depression free day [CESD score <4] to 1 depression free day [CESD ≥10].) | | | | | | | | |
| Kronish 2019 | All | 351.3 (175) days | 339 (176.6) days | 12.3  (-9.5 to 34.1) | - | - | SMD **0.07 higher**  (0.05 lower to 0.19 higher) | ⨁⨁⨁◯  MODERATE |
|  | Women | 317.9 (176.1) days | 306.6 (182.3) days | 11.3  (-30.7 to 53.3) | - | - | SMD **0.06 higher**  (0.17 lower to 0.30 higher) | ⨁⨁◯◯  LOW |
|  | Men | 363.5 (173.4) days | 349.8 (171.7) days | 13.7  (-11.5 to 38.9) | - | - | SMD **0.08 higher**  (0.07 lower to 0.23 higher) | ⨁⨁⨁◯  MODERATE |
| ***Health-related quality of life outcomes*** | | | | | | | | |
| **Change in QALYs** (estimated using the SF-6 dimension, with scores derived from SF-12 v2 responses. Change in QALYs: Observed QALY for 18-month period minus QALY at baseline.) | | | | | | | | |
| Kronish 2019 | All | −0.06 (0.2) | −0.06 (0.18) | 0.00  (-0.02 to 0.02) | - | - | SMD **0.00**  (0.12 lower to 0.12 higher) | ⨁⨁⨁◯  MODERATE |
|  | Women | -0.08 (0.2) | -0.04 (0.17) | -0.04  (-0.08 to 0.00) | - | - | SMD **0.22 lower**  (0.45 lower to 0.02 higher) | ⨁⨁◯◯  LOW |
|  | Men | -0.05 (0.19) | -0.06 (0.18) | 0.01  (-0.02 to 0.04) | - | - | SMD **0.05 higher**  (0.09 lower to 0.20 higher) | ⨁⨁⨁◯  MODERATE |
| **Quality-of-life utility scores** (estimated using the SF-6 dimension, with scores derived from SF-12 v2 responses. Overall assessment of well-being on a scale from 0 [death] to 1 [perfect health].) | | | | | | | | |
| Kronish 2019 | Baseline | 0.78 (0.14) | 0.77 (0.15) | 0.01  (-0.01 to 0.03) | - | - | SMD **0.07 higher**  (0.06 lower to 0.19 higher) | ⨁⨁⨁◯  MODERATE |
|  | 6 months | 0.74 (0.17) | 0.73 (0.17) | 0.01  (-0.01 to 0.03) | - | - | SMD **0.06 higher**  (0.07 lower to 0.18 higher) | ⨁⨁⨁◯  MODERATE |
|  | 12 months | 0.73 (0.20) | 0.73 (0.19) | 0.00  (-0.02 to 0.02) | - | - | SMD **0.00**  (0.12 lower to 0.12 higher) | ⨁⨁⨁◯  MODERATE |
|  | 18 months | 0.71 (0.24) | 0.72 (0.22) | -0.01  (-0.04 to 0.02) | - | - | SMD **0.04 lower**  (0.17 lower to 0.08 higher) | ⨁⨁⨁◯  MODERATE |
| **Mental component** (measured with the SF-MCS. Scores range from 0 to 100, with lower score representing a worse rating) | | | | | | | | |
| Mallen 2017 | Post-consultation | 49.1 (11.2) | 49.9 (11.4) | -0.80  (-2.04 to 0.44) | -0.61  (-1.98 to 0.76) | 0.383 | SMD **0.07 lower**  (0.18 lower to 0.04 higher) | ⨁◯◯◯ VERY LOW |
|  | 3 months | 48.4 (11.5) | 49.6 (11.5) | -1.20  (-2.63 to 0.23) | -0.79  (-2.26 to 0.69) | 0.295 | SMD **0.10 lower**  (0.23 lower to 0.02 higher) | ⨁◯◯◯ VERY LOW |
|  | 6 months | 47.6 (12.0) | 49.0 (11.7) | -1.40  (-6.44 to 3.64) | -0.12  (-1.62 to 1.39) | 0.878 | SMD **0.05 lower**  (0.17 lower to 0.08 higher) | ⨁◯◯◯ VERY LOW |
|  | 12 months | 48.8 (11.6) | 49.2 (11.3) | -0.40  (-1.87 to 1.07) | -0.32  (-1.88 to 1.25) | 0.691 | SMD **0.04 lower**  (0.16 lower to 0.09 higher) | ⨁◯◯◯ VERY LOW |
| **Physical** **component** (measured with the SF-PCS. Scores range from 0 to 100, with lower scores representing a worse rating) | | | | | | | | |
| Mallen 2017 | Post-consultation | 35.5 (10.5) | 36.0 (11.1) | -0.50  (-1.68 to 0.68) | 0.24  (-1.07 to 1.55) | 0.717 | SMD **0.05 lower**  (0.16 lower to 0.06 higher) | ⨁◯◯◯ VERY LOW |
|  | 3 months | 36.3 (10.8) | 37.9 (11.4) | -1.60  (-2.97 to -0.23) | -0.23  (-1.63 to 1.17) | 0.749 | SMD **0.14 lower**  (0.02 lower to 0.27 lower) | ⨁◯◯◯ VERY LOW |
|  | 6 months | **36.3 (11.3)** | **39.3 (11.8)** | **-3.00**  **(-4.45 to -1.55)** | **-1.77**  **(-3.22 to -0.32)** | **0.017** | SMD **0.26 lower**  (0.13 lower to 0.38 lower) | ⨁◯◯◯ VERY LOW |
|  | 12 months | 38.1 (11.6) | 39.1 (11.9) | -1.00  (-2.50 to 0.50) | -0.66  (-2.25 to 0.93) | 0.419 | SMD **0.08 lower**  (0.21 lower to 0.04 higher) | ⨁◯◯◯ VERY LOW |
| **CI**: confidence interval; **EPDS**: Edinburgh Postpartum Depression Score; **GHQ**: General Health Questionnaire; **MD**: mean difference; **PHQ**: Patient Health Questionnaire; **SD**: standard deviation; **SF-PCS**: Medical Outcomes Study Short Form 12 Physical Component Score; **SF-MCS**: Medical Outcomes Study Short Form 12 Mental Component Score; **SMD**: Standardized mean difference  Ϯ All analyses adjusted using general practice and repeated measures as cluster-level random effects, and fixed-effect covariates at practice level and patient level (age, sex, and time between consultation and post-consultation response).  ꬹ This is a cluster trial. The SMD is based on raw data (i.e., not adjusted). Note the that the intraclass correlation coefficient of 0.011 implies very little difference between adjusted and non-adjusted 95% CI | | | | | | | |  |

### Additional file 5.3. Adult results table (binary data)

| **Author Year** | | **Outcome** | **Results: Events/Total No. (%)** | | | **Relative effect**  **(95% CI)** | | **Absolute effect**  **(95%CI)** | | **Certainty** |
| --- | --- | --- | --- | --- | --- | --- | --- | --- | --- | --- |
|  |  |  | **Intervention** | **Control** | |  | |  | |  |
| **Symptoms of depression or diagnosis of MDD outcomes** | | | | | | | | | | |
| **Symptoms** **of** **depression** (measured EPDS. Scores ≥10, with higher scores representing a worse rating) | | | | | | | | | | |
| Leung 2011 | Women (6-mths postpartum/4-mths after randomization) | | 30/215  (14 scored <10 and 16 scored ≥10 at two months) | | 51/215  (45 assessed as negative and six assessed as having probable PND at two months) | -- | -- | | ⨁◯◯◯ VERY LOW | |
|  |  |  | ITT: 30/231 (13.0) | | ITT: 51/231 (22.1) | RR 0.59  (0.39 to 0.89) | 91 fewer per 1000 patients  (24 fewer to 135 fewer per 1000 patients) | |  |  |
| ***Harms of treatment outcomes*** | | | | | | | | | | |
| **Any bleeding** | | | | | | | | | | |
| Kronish 2019 | | 6 months | 51/455 (11.2) | 72/457 (15.8) | | RR 0.71  (0.51 to 0.99) | | 46 fewer per 1000 patients  (77 fewer to 2 fewer per 1000 patients) | | ⨁⨁⨁◯  MODERATE |
|  |  | 12 months | 50/429 (11.7) | 50/427 (11.7) | | RR 1.00  (0.69 to 1.44) | | 0 fewer per 1000 patients  (36 fewer to 52 more per 1000 patients) | | ⨁⨁⨁◯  MODERATE |
|  |  | 18 months | 50/429 (11.7) | 50/427 (11.7) | | RR 1.00  (0.69 to 1.44) | | 0 fewer per 1000 patients  (36 fewer to 52 more per 1000 patients) | | ⨁⨁⨁◯  MODERATE |
| **Increased appetite** | | | | | | | | | | |
| Kronish 2019 | | 6 months | 84/455 (18.5) | 91/457 (19.9) | | RR 0.93  (0.71 to 1.21) | | 14 fewer per 1000 patients  (58 fewer to 42 more per 1000 patients) | | ⨁⨁⨁◯  MODERATE |
|  |  | 12 months | 77/431 (17.9) | 76/427 (17.8) | | RR 1.00  (0.75 to 1.34) | | 0 fewer per 1000 patients  (44 fewer to 61 more per 1000 patients) | | ⨁⨁⨁◯  MODERATE |
|  |  | 18 months | 77/431 (17.9) | 76/427 (17.8) | | RR 1.00  (0.75 to 1.34) | | 0 fewer per 1000 patients  (44 fewer to 61 more per 1000 patients) | | ⨁⨁⨁◯  MODERATE |
| **Decreased appetite** | | | | | | | | | | |
| Kronish 2019 | | 6 months | 74/455 (16.3) | 76/457 (16.6) | | RR 0.98  (0.73 to 1.31) | | 3 fewer per 1000 patients  (45 fewer to 52 more per 1000 patients) | | ⨁⨁⨁◯  MODERATE |
|  |  | 12 months | 65/431 (15.1) | 76/427 (17.8) | | RR 0.85  (0.63 to 1.15) | | 27 fewer per 1000 patients  (66 fewer to 27 more per 1000 patients) | | ⨁⨁⨁◯  MODERATE |
|  |  | 18 months | 65/431 (15.1) | 76/427 (17.8) | | RR 0.85  (0.63 to 1.15) | | 27 fewer per 1000 patients  (66 fewer to 27 more per 1000 patients) | | ⨁⨁⨁◯  MODERATE |
| **Drowsiness** | | | | | | | | | | |
| Kronish 2019 | | 6 months | 212/455 (46.6) | 217/458 (47.4) | | RR 0.98  (0.86 to 1.13) | | 9 fewer per 1000 patients  (66 fewer to 62 more per 1000 patients) | | ⨁⨁⨁◯  MODERATE |
|  |  | 12 months | 187/431 (43.4) | 198/427 (46.4) | | RR 0.94  (0.81 to 1.09) | | 28 fewer per 1000 patients  (88 fewer to 42 more per 1000 patients) | | ⨁⨁⨁◯  MODERATE |
|  |  | 18 months | 187/431 (43.4) | 198/427 (46.4) | | RR 0.94  (0.81 to 1.09) | | 28 fewer per 1000 patients  (88 fewer to 42 more per 1000 patients) | | ⨁⨁⨁◯  MODERATE |
| **Gastrointestinal upset** | | | | | | | | | | |
| Kronish 2019 | | 6 months | 116/454 (25.6) | 112/457 (24.5) | | RR 1.04  (0.83 to 1.31) | | 10 more per 1000 patients  (42 fewer to 76 more per 1000 patients) | | ⨁⨁⨁◯  MODERATE |
|  |  | 12 months | 95/431 (22.0) | 107/427 (25.1) | | RR 0.88  (0.69 to 1.12) | | 30 fewer per 1000 patients  (78 fewer to 30 more per 1000 patients) | | ⨁⨁⨁◯  MODERATE |
|  |  | 18 months | 95/431 (22.0) | 107/427 (25.1) | | RR 0.88  (0.69 to 1.12) | | 30 fewer per 1000 patients  (78 fewer to 30 more per 1000 patients) | | ⨁⨁⨁◯  MODERATE |
| **Adverse events** | | | | | | | | | | |
| Leung 2011 | | Women (6-mths postpartum/4-mths after randomization) | 0/231 (0) | 0/231 (0) | | -- | | -- | | ⨁◯◯◯ VERY LOW |

**CI**: confidence interval; **EPDS**: Edinburgh Postpartum Depression Score; **ITT**: Intention to Treat; **PND**: postnatal depression; **RR**: Risk Ratio

### Additional file 5.4. Adult risk of bias assessment

Shaded rows are evaluated based on class of outcome or outcome, as recommended in the Cochrane Handbook.

***Kronish 2019***

**Patient-reported outcomes**: Depression (depressive symptoms [CESD-10 score, PHQ-8 score], depression-free days [converted CESD-10 score]). Health-related quality of life (change in QALYs [SF-12 & SF-6D], quality-of-life utility scores [converted SF-12 & SF-6D]). Harms of treatment (harms potentially attributable to the use of antidepressant medications [bleeding, increased appetite, decreased appetite, drowsiness, gastrointestinal upset])

**Other outcome**: Mortality

| **Domain** | **Judgement** | **Support** |
| --- | --- | --- |
| Random sequence generation | Low risk | A web-based random number generator integrated into the patient tracking system produced a blocked randomization assignment within strata with randomly selected block sizes of 3, 6, or 9. |
| Allocation concealment | Low risk | Randomization assignment became visible to an unblinded coordinator only after all baseline data had been entered. Concealment was ensured as group allocation occurred in real time using the web-based computer algorithm. |
| Blinding of personnel/ participants *(Patient-reported outcomes)* | Low risk | Participants, site investigators, and other personnel not designated as the blinded coordinator could not be blinded to group allocation but were encouraged to not disclose the allocation when assisting the blinded coordinator and we do not think any deviations from the intended interventions arose. |
| Blinding of personnel/ participants *(Mortality)* | Low risk | Objective outcome which would not be influenced by knowledge of group allocation. |
| Blinding of outcome assessors *(Patient-reported outcomes)* | Low risk | Blinded coordinators conducted all outcome assessments over the phone while masked to group allocation. Unblinded patients completed the CESD-10 and 12-Item Short-Form Health Survey at baseline and during follow-up assessments with the blinded coordinator. The latter included a symptom checklist assessing potential adverse consequences from depression screening and treatment. |
| Blinding of outcome assessors *(Mortality)* | Low risk | Objective outcome assessed by blinded coordinators through review of the electronic medical record. |
| Incomplete outcome data *(Patient-reported outcomes & Mortality)* | Low risk | Reasons for incomplete outcome data were similar across groups with 13% of the intervention group and 12% of the control group not completing the 18-month follow-up or had died. Performed intention-to-treat analyses, used multiple imputation for missing data, and performed sensitivity analyses for missing data. |
| Selective reporting | Low risk | Although the clinical trial registry did not report the outcome on the harms of treatment, the protocol did. The protocol and to a lesser extent the clinical trial registry reported the outcomes on the cost of health care utilization and loss of productivity, this was not reported in the publication, but was explained in the clinical trial registry to be delayed in reporting. |
| Other bias | Low risk | Baseline characteristics were similar between groups. No other concerns. |
| **Overall judgement** | **Low risk**  **Low risk** | Patient reported outcomes  Mortality |

***Leung 2011***

**Mental Health Outcomes**: EPDS score, GHQ-12 score

**Other Outcome**: Harms of treatment (adverse events)

| **Domain** | **Judgement** | **Support** |
| --- | --- | --- |
| Random sequence generation | Low risk | Random numbers were generated by a research officer who was not involved in the rest of the study using a computerized random number generator. |
| Allocation concealment | Low risk | The numbers were placed in sequentially numbered opaque and sealed envelopes. |
| Blinding of personnel/participants (*Mental Health Outcomes*) | Unclear risk | A nurse who was blind to participants’ assignment and scores initially reviewed scores and made treatment recommendations. However, after clinical assessment, a research officer directed patients to further management according to their EPDS scores and/or clinical assessment results. This officer would be able to tell identify which group the participant was allocated to based on whether they had an EPDS score or not. Different treatments were given to these participants, and it is not clear how it was decided. |
| Blinding of personnel/participants *(Harms of treatment)* | Unclear risk | It is unclear how adverse events were reported/collected. |
| Blinding of outcome assessors (*Mental Health Outcomes*) | High risk | Participants completed the questionnaires and would know whether they received treatment and had been assessed for depression. |
| Blinding of outcome assessors *(Harms of treatment)* | Unclear risk | It is unclear how adverse events were reported/collected. |
| Incomplete outcome data (*Mental Health Outcomes*) | Low risk | The same number of participants were missing at 6-months follow-up in both groups (~7% per group). |
| Incomplete outcome data *(Harms of treatment)* | Unclear risk | It is unclear how adverse events were reported/collected. |
| Selective reporting | High risk | Registered in clinicaltrials.gov. GHQ at 6 months was listed as a primary outcome, however this is listed as a secondary outcome in the paper and states that there was no significant different between groups. Minimal information of this outcome is presented. |
| Other bias | Unclear risk | There is no information about who funded the project in the publication, but non-profit organizations are listed in the trials registry. There are no conflicts of interest statements provided. Authors reports that baseline characteristics were “largely the same”, but do not provide any additional information. |
| **Overall judgement** | **High risk**  **High risk** | Mental health outcomes  Harms of treatment |

***Mallen 2017***

**All outcomes**: Depression (PHQ-8 score) and Health-related quality of life (mental component score (SF-MCS) and physical component score (SF-PCS))

| **Domain** | **Judgement** | **Support** |
| --- | --- | --- |
| Random sequence generation | Low risk | General practices were randomized using a balanced algorithm based on practice list size, area deprivation, and clinical commissioning group. |
| Allocation concealment | Low risk | General practices were randomly allocated by the independent statistician on the trial steering committee and then passed to the Primary Care Research Network, who installed the appropriate template into each practice. |
| Blinding of personnel/participants | Low risk | Individual patients were not informed about which arm of the trial they were in. The intervention condition was not disclosed to control practices and the control condition was not disclosed to intervention practices. The chief investigator, principal investigator, trial statistician, and members of the administration team who inputted data from the study questionnaires were blinded to cluster allocation. |
| Blinding of outcome assessors | Low risk | Individual patient outcomes were obtained from postal self-complete questionnaires and medical record review (consenting patients) covering the period up to 12-months post-consultation. Individual patients were not informed about which arm of the trial they were in. |
| Incomplete outcome data | High risk | High loss to follow-up. Only 68% of the control group and 71.3% of the intervention group responded to the post-consultation questionnaire. By 12 months, 48.2% of the control group and 52.8% of the intervention group responded to the questionnaires (among those who responded to the post-consultation questionnaire). There were no missing clusters. |
| Selective reporting | Low risk | Although the clinical trials registry lists the SF-36 and EQ-5D, the protocol posted with the publication lists the SF-12, which was used. The EQ-5D was not reported as the evidence did not lend to performing the health economic analysis. |
| Other bias | Low risk | Some differences between groups, but adjustments (age, sex, and time between consultation and post-consultation response) were made in analysis. With respect to recruitment bias, practices were randomized prior to enrolling participants. Reasons for nonparticipation were similar across groups. Individual patients were not informed to which arm of the trial they were in. Small proportion of those decline to take part, however around 30% in each group of those who were potential eligible did not take part for “reasons not known”. |
| **Overall judgement** | **High risk** |  |

### Additional file 5.5. Adult GRADE evidence profile

#### **Symptoms of depression or diagnosis of MDD outcomes**

| **Certainty assessment** | | | | | | | **№ of patients** | | **Effect** | | **Certainty** | **Importance** |
| --- | --- | --- | --- | --- | --- | --- | --- | --- | --- | --- | --- | --- |
| **№ of studies** | **Study design** | **Risk of bias** | **Inconsistency** | **Indirectness** | **Imprecision** | **Other considerations** | **Screening** | **No screening** | **Absolute (95% CI)** | **SMD**  **(95% CI)** |  |  |
| **Depression score (follow up: baseline; assessed with: CESD-10 (higher scores suggesting a greater severity of depressive symptoms); Scale from: 0 to 30)** | | | | | | | | | | | | |
| 1  (Kronish) | RCT | not serious | not serious | serious ^a^ | not serious | none | 501 | 500 | MD **0.1 higher**  (0.49 lower to 0.69 higher) | SMD **0.02 higher**  (0.10 lower to 0.15 higher) | ⨁⨁⨁◯  MODERATE | Critical |
| **Depression score (follow up: 6 months; assessed with: CESD-10 (higher scores suggesting a greater severity of depressive symptoms); Scale from: 0 to 30)** | | | | | | | | | | | | |
| 1  (Kronish) | RCT | not serious | not serious | serious ^a^ | not serious | none | 501 | 500 | MD **0.1 higher**  (0.59 lower to 0.79 higher) | SMD **0.02 higher**  (0.11 lower to 0.14 higher) | ⨁⨁⨁◯  MODERATE | Critical |
| **Depression score (follow up: 12 months; assessed with: CESD-10 (higher scores suggesting a greater severity of depressive symptoms); Scale from: 0 to 30)** | | | | | | | | | | | | |
| 1  (Kronish) | RCT | not serious | not serious | serious ^a^ | not serious | none | 501 | 500 | MD **0.6 lower**  (1.3 lower to 0.1 higher) | SMD **0.11 lower**  (0.23 lower to 0.02 higher) | ⨁⨁⨁◯  MODERATE | Critical |
| **Depression score (follow up: 18 months; assessed with: CESD-10 (higher scores suggesting a greater severity of depressive symptoms); Scale from: 0 to 30)** | | | | | | | | | | | | |
| 1  (Kronish) | RCT | not serious | not serious | serious ^a^ | not serious | none | 501 | 500 | MD **0.3 lower**  (0.98 lower to 0.38 higher) | SMD **0.06** **lower**  (0.18 lower to 0.07 higher) | ⨁⨁⨁◯  MODERATE | Critical |
| **Change in depressive symptoms among women (range: baseline to 18 months; change in CESD-10 scores)** | | | | | | | | | | | | |
| 1  (Kronish) | RCT | not serious | not serious | serious ^a^ | serious ^b^ | none | 137 | 143 | MD **0.3 lower**  (1.11 lower to 0.51 higher) | SMD **0.09 lower**  (0.32 lower to 0.15 higher) | ⨁⨁◯◯  LOW | Critical |
| **Change in depressive symptoms among men (range: baseline to 18 months; change in CESD-10 scores)** | | | | | | | | | | | | |
| 1  (Kronish) | RCT | not serious | not serious | serious ^a^ | not serious | none | 364 | 355 | MD **0.3 lower**  (0.78 lower to 0.18 higher) | SMD **0.09 lower**  (0.24 lower to 0.06 higher) | ⨁⨁⨁◯  MODERATE | Critical |
| **Depression score (follow up: 18 months; assessed with: PHQ-8 (higher score represents a worse rating); Scale from: 0 to 24)** | | | | | | | | | | | | |
| 1  (Kronish) | RCT | not serious | not serious | serious ^a^ | not serious | none | 501 | 500 | MD **0.1 lower** (0.61 lower to 0.41 higher) | SMD **0.02** **lower**  (0.15 lower to 0.10 higher) | ⨁⨁⨁◯  MODERATE | Critical |
| **Depression score among women (follow up: 18 months; assessed with: PHQ-8 (higher score represents a worse rating); Scale from: 0 to 24)** | | | | | | | | | | | | |
| 1  (Kronish) | RCT | not serious | not serious | serious ^a^ | serious ^b^ | none | 137 | 143 | MD **0.1 higher** (0.98 lower to 1.18 higher) | SMD **0.02** **higher**  (0.21 lower to 0.26 higher) | ⨁⨁◯◯  LOW | Critical |
| **Depression score among men (follow up: 18 months; assessed with: PHQ-8 (higher score represents a worse rating); Scale from: 0 to 24)** | | | | | | | | | | | | |
| 1  (Kronish) | RCT | not serious | not serious | serious ^a^ | not serious | none | 364 | 355 | MD **0.3 lower** (0.91 lower to 0.31 higher) | SMD **0.07** **lower**  (0.22 lower to 0.07 higher) | ⨁⨁⨁◯  MODERATE | Critical |
| **Depression score (follow up: post-consultation;** **assessed with PHQ-8 (higher score represents a worse rating; Scale from 0 to 24))** | | | | | | | | | | | | |
| 1  (Mallen) | RCT | very serious ^c^ | not serious | very serious ^d^ | not serious | none | 493 **^Ϯ^** | 898 **^Ϯ^** | MD **0.30 higher** (0.40 lower to 1.00 higher) ꬹ | SMD **0.07 higher** (0.04 lower to 0.18 higher) **^‡^** | ⨁◯◯◯ VERY LOW | Critical |
| **Depression score (follow up: 3 months;** **assessed with PHQ-8 (higher score represents a worse rating; Scale from 0 to 24))** | | | | | | | | | | | | |
| 1  (Mallen) | RCT | very serious ^c^ | not serious | very serious ^d^ | not serious | none | 383 **^Ϯ^** | 701 **^Ϯ^** | MD **0.52 higher** (0.22 lower to 1.26 higher) ꬹ | SMD **0.14 higher**  (0.01 higher to 0.26 higher) **^‡^** | ⨁◯◯◯ VERY LOW | Critical |
| **Depression score (follow up: 6 months;** **assessed with PHQ-8 (higher score represents a worse rating; Scale from 0 to 24))** | | | | | | | | | | | | |
| 1  (Mallen) | RCT | very serious ^c^ | not serious | very serious ^d^ | not serious | none | 374 **^Ϯ^** | 680 **^Ϯ^** | MD **0.74 higher** (0.02 lower to 1.49 higher) ꬹ | SMD **0.22 higher**  (0.09 higher to 0.35 higher) **^‡^** | ⨁◯◯◯ VERY LOW | Critical |
| **Depression score (follow up: 12 months;** **assessed with PHQ-8 (higher score represents a worse rating; Scale from 0 to 24))** | | | | | | | | | | | | |
| 1  (Mallen) | RCT | very serious ^c^ | not serious | very serious ^d^ | not serious | none | 368 **^Ϯ^** | 644 **^Ϯ^** | MD **0.36 higher** (0.41 lower to 1.14 higher) ꬹ | SMD **0.10 higher**  (0.03 lower to 0.23 higher) **^‡^** | ⨁◯◯◯ VERY LOW | Critical |
| **Cumulative depression-free days (range: baseline to 18 months; assessed with: CESD-10 score converted to depression day. Ranged from 0 depression day [CESD score <4] to 1 depression day [CESD ≥10])** | | | | | | | | | | | | |
| 1  (Kronish) | RCT | not serious | not serious | serious ^a^ | not serious | none | 501 | 500 | MD **12.3 days higher** (9.48 lower to 34.08 higher) | SMD **0.07 higher**  (0.05 lower to 0.19 higher) | ⨁⨁⨁◯  MODERATE | Critical |
| **Cumulative depression-free days among women (range: baseline to 18 months; assessed with: CESD-10 score converted to depression day. Ranged from 0 depression day [CESD score <4] to 1 depression day [CESD ≥10])** | | | | | | | | | | | | |
| 1  (Kronish) | RCT | not serious | not serious | serious ^a^ | serious ^b^ | none | 137 | 143 | MD **11.3 days higher** (30.68 lower to 53.28 higher) | SMD **0.06 higher**  (0.17 lower to 0.30 higher) | ⨁⨁◯◯  LOW | Critical |
| **Cumulative depression-free days among men (range: baseline to 18 months; assessed with: CESD-10 score converted to depression day. Ranged from 0 depression day [CESD score <4] to 1 depression day [CESD ≥10])** | | | | | | | | | | | | |
| 1  (Kronish) | RCT | not serious | not serious | serious ^a^ | not serious | none | 364 | 355 | MD **13.7 days higher** (11.53 lower to 38.93 higher) | SMD **0.08 higher**  (0.07 lower to 0.23 higher) | ⨁⨁⨁◯  MODERATE | Critical |
| **Number identified as depressed among women (follow up: 6-mths postpartum/4-mths after randomization; assessed with: EPDS score ≥ 10, answer positive to question on suicidal ideation)** | | | | | | | | | | | | |
| 1  (Leung) | RCT | very serious ^e,f^ | not serious | serious ^g^ | serious ^h^ | none | 30 | 51 | **91 fewer per 1,000** (24 fewer to 135 fewer) | - | ⨁◯◯◯ VERY LOW | Critical |
| **Depression score among women (follow up: 6-mths postpartum/4-mths after randomization; assessed with: EPDS (higher score represents a worse rating); Scale from: 0 to 30)** | | | | | | | | | | | | |
| 1  (Leung) | RCT | very serious ^e,f^ | not serious | serious ^g^ | serious ^h^ | none | 231 | 231 | MD **1.36 lower** (0.63 lower to 2.09 lower) | SMD **0.34 lower**  (0.15 lower to 0.52 lower) | ⨁◯◯◯ VERY LOW | Critical |
| **Depression score among women (follow up: 6-mths postpartum/4-mths after randomization; assessed with: GHQ-12 (higher score represents a worse rating); Scale from: 0 to 12)** | | | | | | | | | | | | |
| 1  (Leung) | RCT | very serious ^e,f^ | not serious | serious ^g^ | serious ^h^ | none | 231 | 231 | MD **0.33 lower** (0.70 lower to 0.04 higher) | SMD **0.16 lower**  (0.35 lower to 0.02 higher) | ⨁◯◯◯ VERY LOW | Critical |

**CI:** Confidence interval; **EPDS**: Edinburgh Postpartum Depression Score; **GHQ**: General Health Questionnaire; **MD:** Mean difference; **SMD**: Standardized Mean Difference; **RCT**: Randomized Controlled Trial

^Ϯ^ The numbers presented in the table as based on the numbers provided for the primary outcome of pain intensity, and may not be 100% accurate in the number of participants who provided questionnaires at each follow-up time.

ꬹ Adjusted using general practice and repeated measures as cluster-level random effects, and fixed-effect covariates at practice level and patient level (age, sex, and time between consultation and post-consultation response).

‡ Based on raw data (i.e., not adjusted)

**Explanations**

a. Adults with documented acute coronary syndrome

b. The sample size (>800 participants) is not met

c. Large proportion of participants lost to follow-up from those who were screened, to post-consultation and each subsequent time point

d. Adults seeking consultation for osteoarthritis and also receiving screening for anxiety

e. Questionnaire was completed by participants.

f. Selective reporting identified. One of the primary outcomes listed in clinicaltrials.gov was listed as a secondary outcome in the publication. The primary outcome reported as such was statistically significant, and the other was not statistically significant.

g. Postpartum women

h. Due to uncertainty and lack of empirical evidence to support, we have elected not to calculate an OIS. Further, GRADE suggests a rule of thumb of a minimum of 400 events for dichotomous outcomes and 800 participants for continuous outcomes. As there were 462 participants in this study and without a known threshold, a judgement of serious concern was applied.

**Health-related quality of life outcomes**

| **Certainty assessment** | | | | | | | **№ of patients** | | **Effect** | | **Certainty** | **Importance** |
| --- | --- | --- | --- | --- | --- | --- | --- | --- | --- | --- | --- | --- |
| **№ of studies** | **Study design** | **Risk of bias** | **Inconsistency** | **Indirectness** | **Imprecision** | **Other considerations** | **Screening** | **No screening** | **Absolute (95% CI)** | **SMD**  **(95% CI)** |  |  |
| **Change in quality-adjusted life-years (QALYs) (range: baseline to 18 months; assessed with: SF-6 dimension, with scores derived from SF-12 v2 response)** | | | | | | | | | | | | |
| 1  (Kronish) | RCT | not serious | not serious | serious ^a^ | not serious | none | 501 | 500 | MD **0**  (0.02 lower to 0.02 higher) | SMD **0.00**  (0.12 lower to 0.12 higher) | ⨁⨁⨁◯  MODERATE | Critical |
| **Change in quality-adjusted life-years (QALYs) among women (range: baseline to 18 months; assessed with: SF-6 dimension, with scores derived from SF-12 v2 response)** | | | | | | | | | | | | |
| 1  (Kronish) | RCT | not serious | not serious | serious ^a^ | serious ^b^ | none | 137 | 143 | MD **0.04 lower** (0.08 lower to 0) | SMD **0.22 lower**  (0.45 lower to 0.02 higher) | ⨁⨁◯◯  LOW | Critical |
| **Change in quality-adjusted life-years (QALYs) among men (range: baseline to 18 months; assessed with: SF-6 dimension, with scores derived from SF-12 v2 response)** | | | | | | | | | | | | |
| 1  (Kronish) | RCT | not serious | not serious | serious ^a^ | not serious | none | 364 | 355 | MD **0.01 higher** (0.02 lower to 0.04 higher) | SMD **0.05 higher**  (0.09 lower to 0.20 higher) | ⨁⨁⨁◯  MODERATE | Critical |
| **Quality-of-life utility scores (follow up: baseline; assessed with: SF-6 dimension, with scores derived from SF-12 v2 responses; Scale from: 0 (death) to 1 (perfect health))** | | | | | | | | | | | | |
| 1  (Kronish) | RCT | not serious | not serious | serious ^a^ | not serious | none | 501 | 500 | MD **0.01 higher** (0.01 lower to 0.03 higher) | SMD **0.07 higher**  (0.06 lower to 0.19 higher) | ⨁⨁⨁◯  MODERATE | Critical |
| **Quality-of-life utility scores (follow up: 6 months; assessed with: SF-6 dimension, with scores derived from SF-12 v2 responses; Scale from: 0 (death) to 1 (perfect health))** | | | | | | | | | | | | |
| 1  (Kronish) | RCT | not serious | not serious | serious ^a^ | not serious | none | 501 | 500 | MD **0.01 higher** (0.01 lower to 0.03 higher) | SMD **0.06 higher**  (0.07 lower to 0.18 higher) | ⨁⨁⨁◯  MODERATE | Critical |
| **Quality-of-life utility scores (follow up: 12 months; assessed with: SF-6 dimension, with scores derived from SF-12 v2 responses; Scale from: 0 (death) to 1 (perfect health))** | | | | | | | | | | | | |
| 1  (Kronish) | RCT | not serious | not serious | serious ^a^ | not serious | none | 501 | 500 | MD **0**  (0.02 lower to 0.02 higher) | SMD **0.00**  (0.12 lower to 0.12 higher) | ⨁⨁⨁◯  MODERATE | Critical |
| **Quality-of-life utility scores (follow up: 18 months; assessed with: SF-6 dimension, with scores derived from SF-12 v2 responses; Scale from: 0 (death) to 1 (perfect health))** | | | | | | | | | | | | |
| 1  (Kronish) | RCT | not serious | not serious | serious ^a^ | not serious | none | 501 | 500 | MD **0.01 lower** (0.04 lower to 0.02 higher) | SMD **0.04 lower**  (0.17 lower to 0.08 higher) | ⨁⨁⨁◯  MODERATE | Critical |
| **Mental component score (follow up: post-consultation;** **assessed with SF-MCS (lower score represents a worse rating; Scale from 0 to 100))** | | | | | | | | | | | | |
| 1  (Mallen) | RCT | very serious ^c^ | not serious | very serious ^d^ | not serious | none | 493 ^Ϯ^ | 898 ^Ϯ^ | MD **0.61 lower** (1.98 lower to 0.76 higher) ꬹ | SMD **0.07 lower**  (0.18 lower to 0.04 higher) ‡ | ⨁◯◯◯ VERY LOW | Critical |
| **Mental component score (follow up: 3 months;** **assessed with SF-MCS (lower score represents a worse rating; Scale from 0 to 100))** | | | | | | | | | | | | |
| 1  (Mallen) | RCT | very serious ^c^ | not serious | very serious ^d^ | not serious | none | 383 ^Ϯ^ | 701 ^Ϯ^ | MD **0.79 lower** (2.26 lower to 0.69 higher) ꬹ | SMD **0.10 lower**  (0.23 lower to 0.02 higher) ‡ | ⨁◯◯◯ VERY LOW | Critical |
| **Mental component score (follow up: 6 months;** **assessed with SF-MCS (lower score represents a worse rating; Scale from 0 to 100))** | | | | | | | | | | | | |
| 1  (Mallen) | RCT | very serious ^c^ | not serious | very serious ^d^ | not serious | none | 374 ^Ϯ^ | 680 ^Ϯ^ | MD **0.12 lower** (1.62 lower to 1.39 higher) ꬹ | SMD **0.05 lower**  (0.17 lower to 0.08 higher) ‡ | ⨁◯◯◯ VERY LOW | Critical |
| **Mental component score (follow up: 12 months;** **assessed with SF-MCS (lower score represents a worse rating; Scale from 0 to 100))** | | | | | | | | | | | | |
| 1  (Mallen) | RCT | very serious ^c^ | not serious | very serious ^d^ | not serious | none | 368 ^Ϯ^ | 644 ^Ϯ^ | MD **0.32 lower** (1.88 lower to 1.25 higher) ꬹ | SMD **0.04 lower**  (0.16 lower to 0.09 higher) ‡ | ⨁◯◯◯ VERY LOW | Critical |
| **Physical component score (follow up: post-consultation;** **assessed with SF-PCS (lower score represents a worse rating; Scale from 0 to 100))** | | | | | | | | | | | | |
| 1  (Mallen) | RCT | very serious ^c^ | not serious | very serious ^d^ | not serious | none | 493 ^Ϯ^ | 898 ^Ϯ^ | MD **0.24 higher** (1.07 lower to 1.55 higher) ꬹ | SMD **0.05 lower**  (0.15 lower to 0.06 higher) ‡ | ⨁◯◯◯ VERY LOW | Critical |
| **Physical component score (follow up: 3 months;** **assessed with SF-PCS (lower score represents a worse rating; Scale from 0 to 100))** | | | | | | | | | | | | |
| 1  (Mallen) | RCT | very serious ^c^ | not serious | very serious ^d^ | not serious | none | 383 ^Ϯ^ | 701 ^Ϯ^ | MD **0.23 lower** (1.63 lower to 1.17 higher) ꬹ | SMD **0.14 lower**  (0.02 lower to 0.27 lower) ‡ | ⨁◯◯◯ VERY LOW | Critical |
| **Physical component score (follow up: 6 months;** **assessed with SF-PCS (lower score represents a worse rating; Scale from 0 to 100))** | | | | | | | | | | | | |
| 1  (Mallen) | RCT | very serious ^c^ | not serious | very serious ^d^ | not serious | none | 374 ^Ϯ^ | 680 ^Ϯ^ | MD **1.77 lower** (3.22 lower to 0.32 lower) ꬹ | SMD **0.26 lower**  (0.13 lower to 0.38 lower) ‡ | ⨁◯◯◯ VERY LOW | Critical |
| **Physical component score (follow up: 12 months;** **assessed with SF-PCS (lower score represents a worse rating; Scale from 0 to 100))** | | | | | | | | | | | | |
| 1  (Mallen) | RCT | very serious ^c^ | not serious | very serious ^d^ | not serious | none | 368 ^Ϯ^ | 644 ^Ϯ^ | MD **0.66 lower** (2.25 lower to 0.93 higher) ꬹ | SMD **0.08 lower**  (0.21 lower to 0.04 higher) ‡ | ⨁◯◯◯ VERY LOW | Critical |

**CI:** Confidence interval; **MD:** Mean difference; **SMD**: Standardized Mean Difference; **RCT**: Randomized Controlled Trial

^Ϯ^ The numbers presented in the table as based on the numbers provided for the primary outcome of pain intensity, and may not be 100% accurate in the number of participants who provided questionnaires at each follow-up time

ꬹ Adjusted using general practice and repeated measures as cluster-level random effects, and fixed-effect covariates at practice level and patient level (age, sex, and time between consultation and post-consultation response).

‡ Based on raw data (i.e., not adjusted)

**Explanations**

a. Adults with documented acute coronary syndrome

b. The sample size (>800 participants) is not met

c. Large proportion of participants lost to follow-up from those who were screened, to post-consultation and each subsequent time point

d. Adults seeking consultation for osteoarthritis and also receiving screening for anxiety

#### **Harms of treatment outcomes**

| **Certainty assessment** | | | | | | | **№ of patients** | | **Effect** | | **Certainty** | **Importance** |
| --- | --- | --- | --- | --- | --- | --- | --- | --- | --- | --- | --- | --- |
| **№ of studies** | **Study design** | **Risk of bias** | **Inconsistency** | **Indirectness** | **Imprecision** | **Other considerations** | **Screening** | **No screening** | **Relative (95% CI)** | **Absolute (95% CI)** |  |  |
| **Any bleeding (follow up: 6 months; assessed with: patient interview)** | | | | | | | | | | | | |
| 1  (Kronish) | RCT | not serious | not serious | serious ^a^ | not serious | none | 51/455 (11.2%) | 72/457 (15.8%) | RR 0.71  (0.51 to 0.99) | 46 fewer per 1000 patients  (77 to 2 fewer per 1000 patients) | ⨁⨁⨁◯  MODERATE | Critical |
| **Any bleeding (follow up: 12 months; assessed with: patient interview)** | | | | | | | | | | | | |
| 1  (Kronish) | RCT | not serious | not serious | serious ^a^ | not serious | none | 50/429 (11.7%) | 50/427 (11.7%) | RR 1.00  (0.69 to 1.44) | 0 fewer per 1000 patients  (36 fewer to 52 more per 1000 patients) | ⨁⨁⨁◯  MODERATE | Critical |
| **Any bleeding (follow up: 18 months; assessed with: patient interview)** | | | | | | | | | | | | |
| 1  (Kronish) | RCT | not serious | not serious | serious ^a^ | not serious | none | 50/427 (11.7%) | 50/427 (11.7%) | RR 1.00  (0.69 to 1.44) | 0 fewer per 1000 patients  (36 fewer to 52 more per 1000 patients) | ⨁⨁⨁◯  MODERATE | Critical |
| **Increased appetite (follow up: 6 months; assessed with: patient interview)** | | | | | | | | | | | | |
| 1  (Kronish) | RCT | not serious | not serious | serious ^a^ | not serious | none | 84/455 (18.5%) | 91/457 (19.9%) | RR 0.93 (0.71 to 1.21) | 14 fewer per 1,000 (from 58 fewer to 42 more) | ⨁⨁⨁◯  MODERATE | Critical |
| **Increased appetite (follow up: 12 months; assessed with: patient interview)** | | | | | | | | | | | | |
| 1  (Kronish) | RCT | not serious | not serious | serious ^a^ | not serious | none | 77/431 (17.9%) | 76/427 (17.8%) | RR 1.00 (0.75 to 1.34) | 0 fewer per 1,000 (from 44 fewer to 61 more) | ⨁⨁⨁◯  MODERATE | Critical |
| **Increased appetite (follow up: 18 months; assessed with: patient interview)** | | | | | | | | | | | | |
| 1  (Kronish) | RCT | not serious | not serious | serious ^a^ | not serious | none | 77/431 (17.9%) | 76/427 (17.8%) | RR 1.00 (0.75 to 1.34) | 0 fewer per 1,000 (from 44 fewer to 61 more) | ⨁⨁⨁◯  MODERATE | Critical |
| **Decreased appetite (follow up: 6 months; assessed with: patient interview)** | | | | | | | | | | | | |
| 1  (Kronish) | RCT | not serious | not serious | serious ^a^ | not serious | none | 74/455 (16.3%) | 76/457 (16.6%) | **RR 0.98** (0.73 to 1.31) | **3 fewer per 1,000** (from 45 fewer to 52 more) | ⨁⨁⨁◯  MODERATE | Critical |
| **Decreased appetite (follow up: 12 months; assessed with: patient interview)** | | | | | | | | | | | | |
| 1  (Kronish) | RCT | not serious | not serious | serious ^a^ | not serious | none | 65/431 (15.1%) | 76/427 (17.8%) | **RR 0.85** (0.63 to 1.15) | **27 fewer per 1,000** (from 66 fewer to 27 more) | ⨁⨁⨁◯  MODERATE | Critical |
| **Decreased appetite (follow up: 18 months; assessed with: patient interview)** | | | | | | | | | | | | |
| 1  (Kronish) | RCT | not serious | not serious | serious ^a^ | not serious | none | 65/431 (15.1%) | 76/427 (17.8%) | **RR 0.85** (0.63 to 1.15) | **27 fewer per 1,000** (from 66 fewer to 27 more) | ⨁⨁⨁◯  MODERATE | Critical |
| **Drowsiness (follow up: 6 months; assessed with: patient interview)** | | | | | | | | | | | | |
| 1  (Kronish) | RCT | not serious | not serious | serious ^a^ | not serious | none | 212/455 (46.6%) | 217/458 (47.4%) | **RR 0.98** (0.86 to 1.13) | **9 fewer per 1,000** (from 66 fewer to 62 more) | ⨁⨁⨁◯  MODERATE | Critical |
| **Drowsiness (follow up: 12 months; assessed with: patient interview)** | | | | | | | | | | | | |
| 1  (Kronish) | RCT | not serious | not serious | serious ^a^ | not serious | none | 187/431 (43.4%) | 198/427 (46.4%) | **RR 0.94** (0.81 to 1.09) | **28 fewer per 1,000** (from 88 fewer to 42 more) | ⨁⨁⨁◯  MODERATE | Critical |
| **Drowsiness (follow up: 18 months; assessed with: patient interview)** | | | | | | | | | | | | |
| 1  (Kronish) | RCT | not serious | not serious | serious ^a^ | not serious | none | 187/431 (43.4%) | 198/427 (46.4%) | **RR 0.94** (0.81 to 1.09) | **28 fewer per 1,000** (from 88 fewer to 42 more) | ⨁⨁⨁◯  MODERATE | Critical |
| **Gastrointestinal upset (follow up: 6 months; assessed with: patient interview)** | | | | | | | | | | | | |
| 1  (Kronish) | RCT | not serious | not serious | serious ^a^ | not serious | none | 116/454 (25.6%) | 112/457 (24.5%) | **RR 1.04** (0.83 to 1.31) | **10 more per 1,000** (from 42 fewer to 76 more) | ⨁⨁⨁◯  MODERATE | Critical |
| **Gastrointestinal upset (follow up: 12 months; assessed with: patient interview)** | | | | | | | | | | | | |
| 1  (Kronish) | RCT | not serious | not serious | serious ^a^ | not serious | none | 95/431 (22.0%) | 107/427 (25.1%) | **RR 0.88** (0.69 to 1.12) | **30 fewer per 1,000** (from 78 fewer to 30 more) | ⨁⨁⨁◯  MODERATE | Critical |
| **Gastrointestinal upset (follow up: 18 months; assessed with: patient interview)** | | | | | | | | | | | | |
| 1  (Kronish) | RCT | not serious | not serious | serious ^a^ | not serious | none | 95/431 (22.0%) | 107/427 (25.1%) | **RR 0.88** (0.69 to 1.12) | **30 fewer per 1,000** (from 78 fewer to 30 more) | ⨁⨁⨁◯  MODERATE | Critical |
| **Adverse events among women (follow up: 6-mths postpartum/4-mths after randomization; assessed with: not reported)** | | | | | | | | | | | | |
| 1  (Leung) | RCT | very serious ^b,c^ | not serious | serious ^d^ | serious ^e^ | none | 0/231 | 0/231 | Not estimable | Not estimable | ⨁◯◯◯ VERY LOW | Important |

**CI:** Confidence interval; **RCT**: Randomized Controlled Trial

**Explanations**

a. Adults with documented acute coronary syndrome

b. Selective reporting identified. One of the primary outcomes listed in clinicaltrials.gov was listed as a secondary outcome in the publication. The primary outcome reported as such was statistically significant, and the other was not statistically significant.

c. No information on how this information was collected, so many domains were unclear.

d. Postpartum women

e. Due to uncertainty and lack of empirical evidence to support, we have elected not to calculate an OIS. Further, GRADE suggests a rule of thumb of a minimum of 400 events for dichotomous outcomes and 800 participants for continuous outcomes. As there were 462 participants in this study and without a known threshold, a judgement of serious concern was applied.

### Additional file 5.6. Adult summary of findings table

| **Screening compared to no screening for depression in the general adult population** | | | | | | | |
| --- | --- | --- | --- | --- | --- | --- | --- |
| **Patient or population**: depression in the general adult population (adults under consultation for osteoarthritis, with documented acute coronary syndrome, or postpartum women)  **Setting**: General practitioners office or health care systems  **Intervention**: Screening  **Comparison**: No screening | | | | | | | |
| Outcomes | **Anticipated absolute effects^*^** (95% CI) | | | № of participants  (studies) | Certainty of the evidence (GRADE) | Comments |  |
|  | **Risk with no screening** | **Risk with screening** | **Risk with screening** |  |  |  |  |
| Depression score  assessed with: CESD-10 (higher scores suggesting a greater severity of depressive symptoms)  Scale from: 0 to 30  follow up: baseline | The mean depression score was **4.7** | The mean depression score in the intervention group was MD **0.1 higher**  (0.49 lower to 0.69 higher) | SMD **0.02 higher**  (0.10 lower to 0.15 higher) | 1001  (1 RCT) ^1^ | ⨁⨁⨁◯  MODERATE ^a^ |  |  |
| Depression score  assessed with: CESD-10 (higher scores suggesting a greater severity of depressive symptoms)  Scale from: 0 to 30  follow up: 6 months | The mean depression score was **6.0** | The mean depression score in the intervention group was MD **0.1 higher**  (0.59 lower to 0.79 higher) | SMD **0.02 higher**  (0.11 lower to 0.14 higher | 1001  (1 RCT) ^1^ | ⨁⨁⨁◯  MODERATE ^a^ |  |  |
| Depression score  assessed with: CESD-10 (higher scores suggesting a greater severity of depressive symptoms)  Scale from: 0 to 30  follow up: 12 months | The mean depression score was **6.5** | The mean depression score in the intervention group was MD **0.6 lower**  (1.3 lower to 0.1 higher) | SMD **0.11 lower**  (0.23 lower to 0.02 higher) | 1001  (1 RCT) ^1^ | ⨁⨁⨁◯  MODERATE ^a^ |  |  |
| Depression score  assessed with: CESD-10 (higher scores suggesting a greater severity of depressive symptoms)  Scale from: 0 to 30  follow up: 18 months | The mean depression score was **5.6** | The mean depression score in the intervention group was MD **0.3 lower**  (0.98 lower to 0.38 higher) | SMD **0.06 lower**  (0.18 lower to 0.07 higher) | 1001  (1 RCT) ^1^ | ⨁⨁⨁◯  MODERATE ^a^ |  |  |
| Change in depressive symptoms among women (change in CESD-10 scores)  range: baseline to 18 months | The mean change in depressive symptoms was **1.1** | The mean change in depressive symptoms in the intervention group was MD **0.3 lower** (1.11 lower to 0.51 higher) | SMD **0.09 lower**  (0.32 lower to 0.15 higher) | 280  (1 RCT) ^1^ | ⨁⨁◯◯  LOW ^a,b^ |  |  |
| Change in depressive symptoms among men (change in CESD-10 scores)  range: baseline to 18 months | The mean change in depressive symptoms was **1.2** | The mean change in depressive symptoms in the intervention group was MD **0.3 lower** (0.78 lower to 0.18 higher) | SMD **0.09 lower**  (0.24 lower to 0.06 higher) | 719  (1 RCT) ^1^ | ⨁⨁⨁◯  MODERATE ^a^ |  |  |
| Depression score assessed with: PHQ-8 (higher score represents a worse rating) Scale from: 0 to 24 follow up: 18 months | The mean depression score was **3.7** | The mean depression score in the intervention group was MD **0.1 lower** (0.61 lower to 0.41 higher) | SMD **0.02 lower**  (0.15 lower to 0.10 higher) | 1001 (1 RCT) ^1^ | ⨁⨁⨁◯  MODERATE ^a^ |  |  |
| Depression score among women assessed with: PHQ-8 (higher score represents a worse rating) Scale from: 0 to 24 follow up: 18 months | The mean depression score was **4.5** | The mean depression score in the intervention group was MD **0.1 higher** (0.98 lower to 1.18 higher) | SMD **0.02 higher**  (0.21 lower to 0.26 higher) | 280  (1 RCT) ^1^ | ⨁⨁◯◯  LOW ^a,b^ |  |  |
| Depression score among men  assessed with: PHQ-8 (higher score represents a worse rating) Scale from: 0 to 24 follow up: 18 months | The mean depression score was **3.6** | The mean depression score in the intervention group was MD **0.3 lower** (0.91 lower to 0.31 higher) | SMD **0.07 lower**  (0.22 lower to 0.07 higher) | 719  (1 RCT) ^1^ | ⨁⨁⨁◯  MODERATE ^a^ |  |  |
| Depression score assessed with: PHQ-8 (higher score represents a worse rating) Scale from: 0 to 24 follow up: post-consultation | The mean depression score was **6.0** | The mean depression score in the intervention group was MD **0.30 higher** (0.40 lower to 1.00 higher) | SMD **0.07 higher** (0.04 lower to 0.18 higher) | 1391 (1 RCT) ^2^ | ⨁◯◯◯ VERY LOW ^c,d^ |  |  |
| Depression score assessed with: PHQ-8 (higher score represents a worse rating) Scale from: 0 to 24 follow up: 3 months | The mean depression score was **6.5** SD | The mean depression score in the intervention group was **0.52 SD higher** (0.22 lower to 1.26 higher) | SMD **0.14 higher**  (0.01 higher to 0.26 higher) | 1084 (1 RCT) ^2^ | ⨁◯◯◯ VERY LOW ^c,d^ |  |  |
| Depression score assessed with: PHQ-8 (higher score represents a worse rating) Scale from: 0 to 24 follow up: 6 months | The mean depression score was **5.3** | The mean depression score in the intervention group was MD **0.74 higher** (0.02 lower to 1.49 higher) | SMD **0.22 higher**  (0.09 higher to 0.35 higher) | 1054 (1 RCT) ^2^ | ⨁◯◯◯ VERY LOW ^c,d^ |  |  |
| Depression score assessed with: PHQ-8 (higher score represents a worse rating) Scale from: 0 to 24 follow up: 12 months | The mean depression score was **5.4** | The mean depression score in the intervention group was MD **0.36 higher** (0.41 lower to 1.14 higher) | SMD **0.10 higher**  (0.03 lower to 0.23 higher) | 1012 (1 RCT) ^2^ | ⨁◯◯◯ VERY LOW ^c,d^ |  |  |
| Cumulative depression-free days assessed with: CESD-10 (score converted to depression day. Ranged from 0 depression day [CESD score <4] to 1 depression day [CESD ≥10]) range: baseline to 18 months | The mean cumulative depression-free days was **339** days | The mean cumulative depression-free days in the intervention group was MD **12.3 days higher** (9.48 lower to 34.08 higher) | SMD **0.07 higher**  (0.05 lower to 0.19 higher) | 1001  (1 RCT) ^1^ | ⨁⨁⨁◯  MODERATE ^a^ |  |  |
| Cumulative depression-free days among women assessed with: CESD-10 (score converted to depression day. Ranged from 0 depression day [CESD score <4] to 1 depression day [CESD ≥10]) range: baseline to 18 months | The mean cumulative depression-free days among women was **306.6** days | The mean cumulative depression-free days in the intervention group was MD **11.3 days higher** (30.68 lower to 53.28 higher) | SMD **0.06 higher**  (0.17 lower to 0.30 higher) | 280  (1 RCT) ^1^ | ⨁⨁◯◯  LOW ^a,b^ |  |  |
| Cumulative depression-free days among men assessed with: CESD-10 (score converted to depression day. Ranged from 0 depression day [CESD score <4] to 1 depression day [CESD ≥10]) range: baseline to 18 months | The mean cumulative depression-free days among men was **349.8** days | The mean cumulative depression-free days in the intervention group was MD **13.7 days higher** (11.53 lower to 38.93 higher) | SMD **0.08 higher**  (0.07 lower to 0.23 higher) | 719  (1 RCT) ^1^ | ⨁⨁⨁◯  MODERATE ^a^ |  |  |
| Number identified as depressed (EPDS ≥ 10) among women assessed with: EPDS follow up: 6-mths postpartum/4-mths after randomization | 221 per 1,000 | **130 per 1,000** (86 to 196) | **RR 0.59** (0.39 to 0.89) | 462 (1 RCT) ^3^ | ⨁◯◯◯ VERY LOW ^e,f,g,h^ |  | |
| EPDS score among women assessed with: EPDS Scale from: 0 to 30 (worse) follow up: 6-mths postpartum/4-mths after randomization | The mean EPDS score was **6.50** | The mean EPDS score in the intervention group was 1.36 lower (2.09 lower to 0.63 lower) | - | 462 (1 RCT) ^3^ | ⨁◯◯◯ VERY LOW ^e,f,g,h^ |  | |
| GHQ score among women assessed with: GHQ-12 Scale from: 0 to 12 (more severe) follow up: 6-mths postpartum/4-mths after randomization | The mean GHQ score was **1.39** | The mean GHQ score in the intervention group was 0.33 lower (0.70 lower to 0.04 higher) | - | 462 (1 RCT) ^3^ | ⨁◯◯◯ VERY LOW ^e,f,g,h^ |  | |
| Change in quality-adjusted life-years (QALYs) assessed with: SF-6 dimension, with scores derived from SF-12 v2 response range: baseline to 18 months | The mean change in QALYs was  **-0.06** | The mean change in QALYs in the intervention group was MD **0**  (0.02 lower to 0.02 higher) | SMD **0.00**  (0.12 lower to 0.12 higher) | 1001  (1 RCT) ^1^ | ⨁⨁⨁◯  MODERATE ^a^ |  |  |
| Change in quality-adjusted life-years (QALYs) among women assessed with: SF-6 dimension, with scores derived from SF-12 v2 response range: baseline to 18 months | The mean change in QALYs was  **-0.04** | The mean change in QALYs in the intervention group was MD **0.04 lower** (0.08 lower to 0) | SMD **0.22 lower**  (0.45 lower to 0.02 higher) | 280  (1 RCT) ^1^ | ⨁⨁◯◯  LOW ^a,b^ |  |  |
| Change in quality-adjusted life-years (QALYs) among men assessed with: SF-6 dimension, with scores derived from SF-12 v2 response range: baseline to 18 months | The mean change in QALYs was **-0.06** | The mean change in QALYs in the intervention group was MD **0.01 higher** (0.02 lower to 0.04 higher) | SMD **0.05 higher**  (0.09 lower to 0.20 higher) | 719  (1 RCT) ^1^ | ⨁⨁⨁◯  MODERATE ^a^ |  |  |
| Quality-of-life utility scores assessed with: SF-6 dimension, with scores derived from SF-12 v2 responses Scale from: 0 (death) to 1 (perfect health) follow up: baseline | The mean quality-of-life utility scores was **0.77** | The mean quality-of-life utility scores in the intervention group was MD **0.01 higher** (0.01 lower to 0.03 higher) | SMD **0.07 higher**  (0.06 lower to 0.19 higher) | 1001  (1 RCT) ^1^ | ⨁⨁⨁◯  MODERATE ^a^ |  |  |
| Quality-of-life utility scores assessed with: SF-6 dimension, with scores derived from SF-12 v2 responses Scale from: 0 (death) to 1 (perfect health) follow up: 6 months | The mean quality-of-life utility scores was **0.73** | The mean quality-of-life utility scores in the intervention group was MD **0.01 higher** (0.01 lower to 0.03 higher) | SMD **0.06 higher**  (0.07 lower to 0.18 higher) | 1001  (1 RCT) ^1^ | ⨁⨁⨁◯  MODERATE ^a^ |  |  |
| Quality-of-life utility scores assessed with: SF-6 dimension, with scores derived from SF-12 v2 responses Scale from: 0 (death) to 1 (perfect health) follow up: 12 months | The mean quality-of-life utility scores was **0.73** | The mean quality-of-life utility scores in the intervention group was MD **0**  (0.02 lower to 0.02 higher) | SMD **0.00**  (0.12 lower to 0.12 higher) | 1001  (1 RCT) ^1^ | ⨁⨁⨁◯  MODERATE ^a^ |  |  |
| Quality-of-life utility scores assessed with: SF-6 dimension, with scores derived from SF-12 v2 responses Scale from: 0 (death) to 1 (perfect health) follow up: 18 months | The mean quality-of-life utility scores was **0.72** | The mean quality-of-life utility scores in the intervention group was MD **0.01 lower** (0.04 lower to 0.02 higher) | SMD **0.04 lower**  (0.17 lower to 0.08 higher) | 1001  (1 RCT) ^1^ | ⨁⨁⨁◯  MODERATE ^a^ |  |  |
| Health-related quality of life: General health status: Physical component score assessed with: SF-PCS (lower score represents a worse rating) Scale from: 0 to 100 follow up: post-consultation | The mean general health status: Physical component score was **36.0** | The mean general health status: Physical component score in the intervention group was MD **0.24 higher** (1.07 lower to 1.55 higher) | SMD **0.07 lower**  (0.18 lower to 0.04 higher) | 1391 (1 RCT) ^2^ | ⨁◯◯◯ VERY LOW ^c,d^ |  |  |
| Health-related quality of life: General health status: Physical component score assessed with: SF-PCS (lower score represents a worse rating) Scale from: 0 to 100 follow up: 3 months | The mean general health status: Physical component score was **37.9** | The mean general health status: Physical component score in the intervention group was MD **0.23 lower** (1.63 lower to 1.17 higher) | SMD **0.10 lower**  (0.23 lower to 0.02 higher) | 1084 (1 RCT) ^2^ | ⨁◯◯◯ VERY LOW ^c,d^ |  |  |
| Health-related quality of life: General health status: Physical component score assessed with: SF-PCS (lower score represents a worse rating) Scale from: 0 to 100 follow up: 6 months | The mean general health status: Physical component score was **39.3** | The mean general health status: Physical component score in the intervention group was MD **1.77 lower** (3.22 lower to 0.32 lower) | SMD **0.05 lower**  (0.17 lower to 0.08 higher) | 1054 (1 RCT) ^2^ | ⨁◯◯◯ VERY LOW ^c,d^ |  |  |
| Health-related quality of life: General health status: Physical component score assessed with: SF-PCS (lower score represents a worse rating) Scale from: 0 to 100 follow up: 12 months | The mean general health status: Physical component score was **39.1** | The mean general health status: Physical component score in the intervention group was MD **0.66 lower** (2.25 lower to 0.93 higher) | SMD **0.04 lower**  (0.16 lower to 0.09 higher) | 1012 (1 RCT) ^2^ | ⨁◯◯◯ VERY LOW ^c,d^ |  |  |
| Health-related quality of life: General health status: Mental component score assessed with: SF-MCS (lower score represents a worse rating) Scale from: 0 to 100 follow up: post-consultation | The mean general health status: Mental component score was **49.9** | The mean general health status: Mental component score in the intervention group was MD **0.61 lower** (1.98 lower to 0.76 higher) | SMD **0.05 lower**  (0.15 lower to 0.06 higher) | 1391 (1 RCT) ^2^ | ⨁◯◯◯ VERY LOW ^c,d^ |  |  |
| Health-related quality of life: General health status: Mental component score assessed with: SF-MCS (lower score represents a worse rating) Scale from: 0 to 100 follow up: 3 months | The mean general health status: Mental component score was **49.6** | The mean general health status: Mental component score in the intervention group was MD **0.79 lower** (2.26 lower to 0.69 higher) | SMD **0.14 lower**  (0.02 lower to 0.27 lower) | 1084 (1 RCT) ^2^ | ⨁◯◯◯ VERY LOW ^c,d^ |  |  |
| Health-related quality of life: General health status: Mental component score assessed with: SF-MCS (lower score represents a worse rating) Scale from: 0 to 100 follow up: 6 months | The mean general health status: Mental component score was **49.0** | The mean general health status: Mental component score in the intervention group was MD **0.12 lower** (1.62 lower to 1.39 higher) | SMD **0.26 lower**  (0.13 lower to 0.38 lower) | 1054 (1 RCT) ^2^ | ⨁◯◯◯ VERY LOW ^c,d^ |  |  |
| Health-related quality of life: General health status: Mental component score assessed with: SF-MCS (lower score represents a worse rating) Scale from: 0 to 100 follow up: 12 months | The mean general health status: Mental component score was **49.2** | The mean general health status: Mental component score in the intervention group was MD **0.32 lower** (1.88 lower to 1.25 higher) | SMD **0.08 lower**  (0.21 lower to 0.04 higher) | 1012 (1 RCT) ^2^ | ⨁◯◯◯ VERY LOW ^c,d^ |  |  |
| Any bleeding assessed with: patient interview follow up: 6 months | 158 per 1,000 | **112 per 1,000** (80 to 156) | **46 fewer per 1000 patients**  (77 to 2 fewer per 1000 patients) | 912 (1 RCT) ^1^ | ⨁⨁⨁◯  MODERATE ^a^ |  |  |
| Any bleeding assessed with: patient interview follow up: 12 months | 117 per 1,000 | **117 per 1,000** (81 to 169) | **0 fewer per 1000 patients**  (36 fewer to 52 more per 1000 patients) | 856 (1 RCT) ^1^ | ⨁⨁⨁◯  MODERATE ^a^ |  |  |
| Any bleeding assessed with: patient interview follow up: 18 months | 117 per 1,000 | **117 per 1,000** (81 to 169) | **0 fewer per 1000 patients**  (36 fewer to 52 more per 1000 patients) | 856 (1 RCT) ^1^ | ⨁⨁⨁◯  MODERATE ^a^ |  |  |
| Increased appetite  assessed with: patient interview follow up: 6 months | 199 per 1,000 | **185 per 1,000** (141 to 241) | **14 fewer per 1,000**  (from 58 fewer to 42 more) | 912 (1 RCT) ^1^ | ⨁⨁⨁◯  MODERATE ^a^ |  |  |
| Increased appetite assessed with: patient interview follow up: 12 months | 178 per 1,000 | **178 per 1,000** (133 to 239) | **0 fewer per 1,000** (from 44 fewer to 61 more) | 858 (1 RCT) ^1^ | ⨁⨁⨁◯  MODERATE ^a^ |  |  |
| Increased appetite assessed with: patient interview follow up: 18 months | 178 per 1,000 | **178 per 1,000** (133 to 239) | **0 fewer per 1,000** (from 44 fewer to 61 more) | 858 (1 RCT) ^1^ | ⨁⨁⨁◯  MODERATE ^a^ |  |  |
| Decreased appetite assessed with: patient interview follow up: 6 months | 166 per 1,000 | **163 per 1,000** (121 to 218) | **3 fewer per 1,000** (from 45 fewer to 52 more) | 912 (1 RCT) ^1^ | ⨁⨁⨁◯  MODERATE ^a^ |  |  |
| Decreased appetite assessed with: patient interview follow up: 12 months | 178 per 1,000 | **151 per 1,000** (112 to 205) | **27 fewer per 1,000** (from 66 fewer to 27 more) | 858 (1 RCT) ^1^ | ⨁⨁⨁◯  MODERATE ^a^ |  |  |
| Decreased appetite  assessed with: patient interview follow up: 18 months | 178 per 1,000 | **151 per 1,000** (112 to 205) | **27 fewer per 1,000** (from 66 fewer to 27 more) | 858 (1 RCT) ^1^ | ⨁⨁⨁◯  MODERATE ^a^ |  |  |
| Drowsiness assessed with: patient interview follow up: 6 months | 474 per 1,000 | **464 per 1,000** (407 to 535) | **9 fewer per 1,000** (from 66 fewer to 62 more) | 913 (1 RCT) ^1^ | ⨁⨁⨁◯  MODERATE ^a^ |  |  |
| Drowsiness assessed with: patient interview follow up: 12 months | 464 per 1,000 | **436 per 1,000** (376 to 505) | **28 fewer per 1,000** (from 88 fewer to 42 more) | 858 (1 RCT) ^1^ | ⨁⨁⨁◯  MODERATE ^a^ |  |  |
| Drowsiness assessed with: patient interview follow up: 18 months | 464 per 1,000 | **436 per 1,000** (376 to 505) | **28 fewer per 1,000** (from 88 fewer to 42 more) | 858 (1 RCT) ^1^ | ⨁⨁⨁◯  MODERATE ^a^ |  |  |
| Gastrointestinal upset  assessed with: patient interview follow up: 6 months | 245 per 1,000 | **255 per 1,000** (203 to 321) | **10 more per 1,000** (from 42 fewer to 76 more) | 911 (1 RCT) ^1^ | ⨁⨁⨁◯  MODERATE ^a^ |  |  |
| Gastrointestinal upset  assessed with: patient interview follow up: 12 months | 251 per 1,000 | **221 per 1,000** (173 to 281) | **30 fewer per 1,000** (from 78 fewer to 30 more) | 858 (1 RCT) ^1^ | ⨁⨁⨁◯  MODERATE ^a^ |  |  |
| Gastrointestinal upset assessed with: patient interview follow up: 18 months | 251 per 1,000 | **221 per 1,000** (173 to 281) | **30 fewer per 1,000** (from 78 fewer to 30 more) | 858 (1 RCT) ^1^ | ⨁⨁⨁◯  MODERATE ^a^ |  |  |
| Harms of treatment: Adverse events among women  follow up: 6-mths postpartum/4-mths after randomization | Not estimable | Not estimable | Not estimable | 462  (1 RCT) ^3^ | ⨁◯◯◯ VERY LOW ^f,g,h,i^ | It is unclear how data on adverse events was collected or reported. Reported only as “No adverse events were  reported throughout the study” | |
| ***The risk in the intervention group** (and its 95% confidence interval) is based on the assumed risk in the comparison group and the **relative effect** of the intervention (and its 95% CI).  **CI:** Confidence interval; **MD:** Mean difference; **SMD**: Standardized mean difference | | | | | | | |
| **GRADE Working Group grades of evidence** **High certainty:** We are very confident that the true effect lies close to that of the estimate of the effect **Moderate certainty:** We are moderately confident in the effect estimate: The true effect is likely to be close to the estimate of the effect, but there is a possibility that it is substantially different **Low certainty:** Our confidence in the effect estimate is limited: The true effect may be substantially different from the estimate of the effect **Very low certainty:** We have very little confidence in the effect estimate: The true effect is likely to be substantially different from the estimate of effect | | | | | | | |

**Explanations**

a. Adults with documented acute coronary syndrome

b. The sample size (>800 participants) is not met

c. Large proportion of participants lost to follow-up from those who were screened, to post-consultation and each subsequent time point

d. Adults seeking consultation for osteoarthritis and also screened for anxiety

e. Questionnaire was completed by participants.

f. Selective reporting identified. One of the primary outcomes listed in clinicaltrials.gov was listed as a secondary outcome in the publication. The primary outcome reported as such was statistically significant, and the other was not statistically significant.

g. Postpartum women

h. Due to uncertainty and lack of empirical evidence to support, we have elected not to calculate an OIS. Further, GRADE suggests a rule of thumb of a minimum of 400 events for dichotomous outcomes and 800 participants for continuous outcomes. As there were 462 participants in this study and without a known threshold, a judgement of serious concern was applied. i. No information on how this information was collected, so many domains were unclear.

**References**

1. Kronish, I.M., Moise, N., Cheung, Y.K., Clarke, G.N., Dolor, R.J., Duer-Hefele, J., Margolis, K.L., St Onge, T., Parsons, F., Retuerto, J. and Thanataveerat, A., 2019. Effect of depression screening after acute coronary syndromes on quality of life: the CODIACS-QoL randomized clinical trial. JAMA Internal Medicine, 180(1), pp.45-53.
2. Mallen, C.D., Nicholl, B.I., Lewis, M., Bartlam, B., Green, D., Jowett, S., Kigozi, J., Belcher, J., Clarkson, K., Lingard, Z. and Pope, C., 2017. The effects of implementing a point-of-care electronic template to prompt routine anxiety and depression screening in patients consulting for osteoarthritis (the Primary Care Osteoarthritis Trial): A cluster randomised trial in primary care. PLoS medicine, 14(4), p.e1002273.
3. Leung, S.S., Leung, C., Lam, T.H., Hung, S.F., Chan, R., Yeung, T., Miao, M., Cheng, S., Leung, S.H., Lau, A. and Lee, D.T., 2011. Outcome of a postnatal depression screening programme using the Edinburgh Postnatal Depression Scale: a randomized controlled trial. Journal of Public Health, 33(2), pp.292-301.

## Additional file 6. Pregnancy and postpartum evidence sets

### Additional file 6.1. Postpartum study characteristics table

| **Author Year**  **Funding** | **Participants, Study design, and Location** | **Intervention (n=231)** | **Control (n=231)** | **Outcomes (n=462)** | **Notes** |
| --- | --- | --- | --- | --- | --- |
| Leung 2011, Hong Kong, China  **Funding**: Not reported. Sponsors and collaborators were Chinese University of Hong Kong, Hong Kong Department of Health, and The University of Hong Kong | 462 Chinese mothers with 2 month-old babies visiting Maternal and Child Health Centres in Hong Kong.  Those who participated in other postnatal depression screening programmes or were receiving psychiatric treatment were excluded.  **Study design**: Randomized controlled trial  **Location**: Maternal and Child Health Centres in Hong Kong | The Edinburgh Postnatal Depression Scale was used to identify participants with postnatal depression. Women were also clinically assessed.  The EPDS has 10 questions, and scores range from 0-30 (worse). Those with scores above the cut-off (≥10) or suicidal ideation were offered non-directive counselling by MCH nurses or management by the community psychiatric team as appropriate.  The Chinese version of the EPDS was validated with Hong Kong women at 6 weeks postnatal, against the structured clinical interview for DSM-III-R.  Treatment: 55 received treatment (46 received counselling by MCH nurses, of whom eight received additional psychiatric nurse counselling whereas three received psychiatric nurse counselling and treatment by a psychiatrist; another 8 received psychiatric nurse counselling initially, with one of them receiving further treatment by a psychiatrist; and 1 participant received treatment by a psychiatrist only) | General Self-Efficacy Scale plus usual practice where nurses carried out clinical assessment.  Mothers deemed to require further management were offered non-directive counselling or psychiatric referral.  Treatment: 11 received treatment (10 received MCH nurse counselling initially; four of whom received further psychiatric nurse counselling, with one of these having received additional psychiatric treatment; 1 participant received counselling by a psychiatric nurse only). | **Depression:** measured with the EPDS (score ≥10 (score 0-30)). A higher score represents a worse rating.  **Adverse events**: unclear how this was collected/reported  **General health**: measured with GHQ (score 0-12). A higher score represents a worse rating.  **Parental Stress**: measured with the Parental Stress index which includes a total score and three subscale scores (parental distress, parent-child dysfunctional interaction, difficult child) (total score 0-180, 0-60 per subscale). A higher score represents a worse rating.  **Marital satisfaction score**: measured with the Chinese Kansas Marital Satisfaction Scale (score 3-21). A higher score represents more satisfaction.  **Infant body weight in kg**: assessed with maternal and child health centre records.  **Infant number of hospitalizations**: as reported by mothers/caregivers.  **Follow-up:** 2 months postpartum (baseline), 6 months postpartum (i.e., 4 months after randomization), 18 months postpartum (i.e., 16 months after randomization).  *At 18 months postpartum, the control group had received the EPDS at 6-months postpartum and offered treatment/follow-up services for those who scored ≥10, thereby removing the screened versus not screened comparison.* | The clinical assessment included observing  participants’ expression and behaviour, enquiring about  feelings, appetite, sleep pattern, childcare and suicidal ideas. |

**DSM:** Diagnostic and Statistical Manual of Mental Disorders, Third edition-Revised; **EPDS**: Edinburgh Postpartum Depression Score; **GHQ**: General Health Questionnaire; **kg**: kilograms; **MCH**: Maternal and Child Health Centres

### Additional file 6.2. Postpartum results table (binary)

| **Author Year** | **Outcome (timing)** | **Results [n/N (95% CI)]** | |  | | **Absolute effect (95% CI)** |
| --- | --- | --- | --- | --- | --- | --- |
|  |  | **Intervention** | **Control** | **RR (95% CI)** | **p-value** |  |
| ***Maternal mental health outcome****: Symptoms of depression (EPDS score ≥10)* | | | | | | |
| Leung 2011 | Number with EPDS score ≥10 (6 months postpartum) | 30/215  (14 scored <10 and 16 scored ≥10 at two months) | 51/215  (45 assessed as negative and six assessed as having probable PND at two months) |  |  |  |
|  |  | **ITT: 30/231** | **ITT: 51/231** | **0.59**  **(0.39 to 0.89)** | **0.01** | **91 fewer per 1,000**  (24 fewer to 135 fewer) |
| ***Maternal outcome****: Harms of treatment* | | | | | | |
| Leung 2011 | Adverse events (at 6 months postpartum) | 0/231 | 0/231 | -- | -- | -- |
| **CI**: confidence interval; **EPDS**: Edinburgh Postpartum Depression Score; **ITT**: Intention to Treat; **PND**: postnatal depression; **RR**: Risk Ratio | | | | | | |

### Additional file 6.3. Postpartum results table (continuous data)

| **Author Year** | **Outcome (timing)** | **Results [mean (95% confidence interval)]** | | | **Absolute effect (95% confidence interval)** | |
| --- | --- | --- | --- | --- | --- | --- |
|  |  | **Intervention** | **Control** | **p-value** | **MD** | **SMD** |
| ***Maternal mental health outcome****: Symptoms of depression (assessed with: EPDS; Scale from: 0 to 30 (worse))* | | | | | | |
| Leung 2011 | EPDS score (6 months postpartum) | **5.14**  **(4.67 to 5.60)** | **6.50**  **(5.94 to 7.07)** | **<0.001** | MD 1.36 lower (0.63 lower to 2.09 lower) | SMD 0.34 lower  (0.15 lower to 0.52 lower) |
| ***Maternal mental health outcome****: Symptoms of depression (assessed with: GHQ; Scale from: 0 to 12 (worse))* | | | | | | |
| Leung 2011 | GHQ score (6 months postpartum) | 1.06  (0.83 to 1.30) | 1.39  (1.10 to 1.67) | 0.084 | MD 0.33 lower (0.7 lower to 0.04 higher) | SMD 0.16 lower  (0.35 lower to 0.02 higher) |
| ***Parenting & Relationship outcomes****: mother-child interactions, Relationship with partner and other supports* | | | | | | |
| Leung 2011 | PSI-Total score (6 months postpartum) [scale 0-180] | 80.89  (78.80 to 82.97) | 83.67  (81.56 to 85.77) | 0.065 | MD 2.78 lower (5.74 lower to 0.18 higher) | SMD 0.17 lower  (0.35 lower to 0.01 higher) |
| Leung 2011 | PSI-Parental Distress subscale score (6 months postpartum) [scale 0-60] | 29.93  (29.03 to 30.84) | 31.14  (30.24 to 32.03) | 0.063 | MD 1.21 lower (2.48 lower to 0.06 higher) | SMD 0.17 lower  (0.36 lower to 0.01 higher) |
| Leung 2011 | PSI-Parent-child dysfunctional interaction subscale score (6 months postpartum) [scale 0-60] | 24.77  (24.03 to 25.51) | 25.85  (25.05 to 26.65) | 0.05 | MD 1.08 lower (2.17 lower to 0.01 higher) | SMD 0.18 lower  (0.36 lower to 0.00) |
| Leung 2011 | PSI-Difficult Child subscale score (6 months postpartum) [scale 0-60] | 26.19  (25.37 to 27.01) | 26.68  (25.88 to 27.48) | 0.397 | MD 0.49 lower (1.64 lower to 0.66 higher) | SMD 0.08 lower  (0.26 lower to 0.10 higher) |
| Leung 2011 | Marital satisfaction score (6 months postpartum) | 16.94  (16.59 to 17.30) | 16.47  (16.03 to 16.90) | 0.093 | MD 0.47 higher (0.09 lower to 1.03 higher) | SMD 0.15 higher  (0.03 lower to 0.34 higher) |
| ***Infant Outcomes****: Infant health and development* | | | | | | |
| Leung 2011 | Infant body weight in kg (6 months postpartum) | 7.71  (7.60 to 7.82) | 7.66  (7.56 to 7.76) | 0.504 | MD 0.05 higher (0.1 lower to 0.2 higher) | SMD 0.06 higher  (0.12 lower to 0.24 higher) |
| Leung 2011 | Infant number of hospitalizations (6 months postpartum) | 0.37  (0.28 to 0.46) | 0.33  (0.23 to 0.42) | 0.518 | MD 0.04 higher (0.09 lower to 0.17 higher) | SMD 0.06 higher  (0.13 lower to 0.24 higher) |
| **EPDS**: Edinburgh Postpartum Depression Score; **GHQ**: General Health Questionnaire; **MD**: mean difference; **PSI**: Parenting Stress Index; **SMD**: Standardized mean difference | | | | | | |

### Additional file 6.4. Postpartum risk of bias assessments

Highlighted rows are evaluated based on class of outcome or outcome, as recommended in the Cochrane Handbook.

***Leung 2011***

**Maternal/Parenting Outcomes**: EPDS score, GHQ score, Parental Stress Index score (including subscales), and Marital Satisfaction score at 6 months

| **Domain** | **Judgement** | **Support** |
| --- | --- | --- |
| Random sequence generation | Low risk | Random numbers were generated by a research officer who was not involved in the rest of the study using a computerized random number generator. |
| Allocation concealment | Low risk | The numbers were placed in sequentially numbered opaque and sealed envelopes. |
| Blinding of personnel/participants | Unclear risk | A nurse who was blind to participants’ assignment and scores initially reviewed scores and made treatment recommendations. However, after clinical assessment, a research officer directed patients to further management according to their EPDS scores and/or clinical assessment results. This officer would be able to tell identify which group the participant was allocated to based on whether they had an EPDS score or not. Different treatments were given to these participants, and it is not clear how it was decided. |
| Blinding of outcome assessors | High risk | Participants completed the questionnaires and would know whether they received treatment and had been assessed for depression. |
| Incomplete outcome data | Low risk | The same number of participants were missing at 6-months follow-up in both groups (~7% per group). |
| Selective reporting | High risk | Registered in clinicaltrials.gov. GHQ at 6 months was listed as a primary outcome, however this is listed as a secondary outcome in the paper and states that there was no significant different between groups. Minimal information of this outcome is presented. |
| Other bias | Unclear risk | There is no information about who funded the project in the publication, but non-profit organizations are listed in the trials registry. There are no conflicts of interest statements provided. Authors reports that baseline characteristics were “largely the same”, but do not provide any additional information. |
| **Overall judgement** | **High risk** |  |

***Leung 2011***

**Maternal/Parenting & Relationship Outcomes**: Harms of treatment (adverse events)

| **Domain** | **Judgement** | **Support** |
| --- | --- | --- |
| Random sequence generation | Low risk | Random numbers were generated by a research officer who was not involved in the rest of the study using a computerized random number generator. |
| Allocation concealment | Low risk | The numbers were placed in sequentially numbered opaque and sealed envelopes. |
| Blinding of personnel/participants | Unclear risk | It is unclear how adverse events were reported/collected. |
| Blinding of outcome assessors | Unclear risk | It is unclear how adverse events were reported/collected |
| Incomplete outcome data | Unclear risk | It is unclear how adverse events were reported/collected. |
| Selective reporting | High risk | Registered in clinicaltrials.gov. GHQ at 6 months was listed as a primary outcome, however this is listed as a secondary outcome in the paper and states that there was no significant different between groups. Minimal information of this outcome is presented. Adverse events was not listed as an outcome. |
| Other bias | Unclear risk | There is no information about who funded the project in the publication, but non-profit organizations are listed in the trials registry. There are no conflicts of interest statements provided. Authors reports that baseline characteristics were “largely the same”, but do not provide any additional information. |
| **Overall judgement** | **High risk** |  |

***Leung 2011***

**Infant Outcome**: Infant body weight at 6 months

| **Domain** | **Judgement** | **Support** |
| --- | --- | --- |
| Random sequence generation | Low risk | Random numbers were generated by a research officer who was not involved in the rest of the study using a computerized random number generator. |
| Allocation concealment | Low risk | The numbers were placed in sequentially numbered opaque and sealed envelopes. |
| Blinding of personnel/participants | Low risk | Objective outcome that would not be influenced by knowledge of group allocation. |
| Blinding of outcome assessors | Low risk | Body weight taken from MCHC records. It is unlikely those taking the body weight would know about screening status. It would also not influence the results. |
| Incomplete outcome data | Unclear risk | Authors do not state how many children contributed to this outcome and use an intent to treat analysis. They state that substituting the missing values by the overall mean of participants in both groups was used, but it is unclear if this was specific to the EPDS outcome or all outcomes. |
| Selective reporting | High risk | Registered in clinicaltrials.gov. GHQ at 6 months was listed as a primary outcome, however this is listed as a secondary outcome in the paper and states that there was no significant different between groups. Minimal information of this outcome is presented. |
| Other bias | Unclear risk | There is no information about who funded the project in the publication, but non-profit organizations are listed in the trials registry. There are no conflicts of interest statements provided. Authors reports that baseline characteristics were “largely the same”, but do not provide any additional information, and there appears to be some differences between groups in prognostic factors. |
| **Overall judgement** | **Moderate risk** |  |

***Leung 2011***

**Infant Outcomes**: Number of hospitalization at 6 months

| **Domain** | **Judgement** | **Support** |
| --- | --- | --- |
| Random sequence generation | Low risk | Random numbers were generated by a research officer who was not involved in the rest of the study using a computerized random number generator. |
| Allocation concealment | Low risk | The numbers were placed in sequentially numbered opaque and sealed envelopes. |
| Blinding of personnel/participants | Unclear risk | It is unclear who would make the decision to bring the baby to the hospital (e.g., mothers, fathers, caregivers). |
| Blinding of outcome assessors | High risk | Self-reported by mothers/caregivers. Depression status could influence the decision to take babies to the hospital and could also influence reporting of this. |
| Incomplete outcome data | Unclear risk | Authors do not state how many children contributed to this outcome and use an intent to treat analysis. They state that substituting the missing values by the overall mean of participants in both groups was used, but it is unclear if this was specific to the EPDS outcome or all outcomes. |
| Selective reporting | High risk | Registered in clinicaltrials.gov. GHQ at 6 months was listed as a primary outcome, however this is listed as a secondary outcome in the paper and states that there was no significant different between groups. Minimal information of this outcome is presented. |
| Other bias | Unclear risk | There is no information about who funded the project in the publication, but non-profit organizations are listed in the trials registry. There are no conflicts of interest statements provided. Authors reports that baseline characteristics were “largely the same”, but do not provide any additional information, and there appears to be some differences between groups in prognostic factors. |
| **Overall judgement** | **High risk** |  |

### Additional file 6.5. Postpartum GRADE evidence profile

**Maternal health outcomes**

| **Certainty assessment** | | | | | | | **№ of patients** | | | **Effect** | | **Certainty** | **Importance** |
| --- | --- | --- | --- | --- | --- | --- | --- | --- | --- | --- | --- | --- | --- |
| **№ of studies** | **Study design** | **Risk of bias** | **Inconsistency** | **Indirectness** | **Imprecision** | **Other considerations** | **Screening** | **No screening** | **Absolute (95% CI)**  **[MD]** | | **Absolute**  **(95% CI)**  **[SMD]** |  |  |
| Depression: Number identified as depressed (follow up: 6 months postpartum; assessed with: EPDS score ≥ 10, answer positive to question on suicidal ideation) | | | | | | | | | | | | | |
| 1  (Leung) | RCT | very serious ^a,b^ | not serious | not serious | serious ^c^ | none | 30/231 (13.0%) | 51/231 (22.1%) | **91 fewer per 1,000** (from 24 fewer to 135 fewer) | | - | ⨁◯◯◯ VERY LOW | CRITICAL |
| Depression: EPDS score (follow up: 6 months postpartum; assessed with: EPDS; Scale from: 0 to 30 (worse)) | | | | | | | | | | | | | |
| 1  (Leung) | RCT | very serious ^a,b^ | not serious | not serious | serious ^c^ | none | 231 | 231 | MD **1.36 lower** (0.63 lower to 2.09 lower) | | SMD **0.34 lower**  (0.15 lower to 0.52 lower) | ⨁◯◯◯ VERY LOW | CRITICAL |
| Symptoms of depression: GHQ score (follow up: 6 months postpartum; assessed with: GHQ-12; Scale from: 0 to 12 (more severe)) | | | | | | | | | | | | | |
| 1  (Leung) | RCT | very serious ^a,b^ | not serious | not serious | serious ^c^ | none | 231 | 231 | MD **0.33 lower** (0.70 lower to 0.04 higher) | | SMD **0.16 lower**  (0.35 lower to 0.02 higher) | ⨁◯◯◯ VERY LOW | CRITICAL |
| Harms of treatment: Adverse events (follow up: 6 months postpartum; assessed with: not reported) | | | | | | | | | | | | | |
| 1  (Leung) | RCT | very serious ^b,d^ | not serious | not serious | serious ^c^ | none | 0/231 | 0/231 | Not estimable | | Not estimable | ⨁◯◯◯ VERY LOW | IMPORTANT |

**CI:** Confidence interval; **GHQ**: General Health Questionnaire; **MD:** Mean difference; **RCT**: Randomized Controlled Trial; **SMD**: Standardized Mean difference

**Explanations**

a. Questionnaire was completed by participants.

b. Selective reporting identified. One of the primary outcomes listed in clinicaltrials.gov was listed as a secondary outcome in the publication. The primary outcome reported as such was statistically significant, and the other was not statistically significant.

c. Due to uncertainty and lack of empirical evidence to support, we have elected not to calculate an OIS. Further, GRADE suggests a rule of thumb of a minimum of 400 events for dichotomous outcomes and 800 participants for continuous outcomes. As there were 462 participants in this study and without a known threshold, a judgement of serious concern was applied.

d. No information on how this information was collected, so many domains were unclear.

**Parenting & Relationship outcomes**

| **Certainty assessment** | | | | | | | **№ of patients** | | **Effect** | | **Certainty** | **Importance** |
| --- | --- | --- | --- | --- | --- | --- | --- | --- | --- | --- | --- | --- |
| **№ of studies** | **Study design** | **Risk of bias** | **Inconsistency** | **Indirectness** | **Imprecision** | **Other considerations** | **Screening** | **No screening** | **Absolute (95% CI)**  **[MD]** | **Absolute**  **(95% CI)**  **[SMD]** |  |  |
| Parenting Stress Index (PSI) total score (follow up: 6 months postpartum; assessed with: PSI-SF; Scale from: 36 to 180 (more stress)) | | | | | | | | | | | | |
| 1  (Leung) | RCT | very serious ^a,b^ | not serious | not serious | serious ^c^ | none | 231 | 231 | MD **2.78 lower** (5.74 lower to 0.18 higher) | SMD **0.17 lower**  (0.35 lower to 0.01 higher) | ⨁◯◯◯ VERY LOW | IMPORTANT |
| PSI-Parental Distress (PD) subscale score (follow up: 6 months postpartum; assessed with: PSI-SF PD subscale; Scale from: 12 to 60 (more stress)) | | | | | | | | | | | | |
| 1  (Leung) | RCT | very serious ^a,b^ | not serious | not serious | serious ^c^ | none | 231 | 231 | MD **1.21 lower** (2.48 lower to 0.06 higher) | SMD **0.17 lower**  (0.36 lower to 0.01 higher) | ⨁◯◯◯ VERY LOW | IMPORTANT |
| PSI-Parent-Child Dysfunctional Interaction (PCDI) subscale score (follow up: 6 months postpartum; assessed with: PSI-SF PCDI subscale; Scale from: 12 to 60 (more stress)) | | | | | | | | | | | | |
| 1  (Leung) | RCT | very serious ^a,b^ | not serious | not serious | serious ^c^ | none | 231 | 231 | MD **1.08 lower** (2.17 lower to 0.01 higher) | SMD **0.18 lower**  (0.36 lower to 0.00) | ⨁◯◯◯ VERY LOW | IMPORTANT |
| PSI-Difficult Child (DC) subscale score (follow up: 6 months postpartum; assessed with: PSI-SF DC subscale; Scale from: 12 to 60 (more stress)) | | | | | | | | | | | | |
| 1  (Leung) | RCT | very serious ^a,b^ | not serious | not serious | serious ^c^ | none | 231 | 231 | MD **0.49 lower** (1.64 lower to 0.66 higher) | SMD **0.08 lower**  (0.26 lower to 0.10 higher) | ⨁◯◯◯ VERY LOW | IMPORTANT |
| Marital satisfaction score (follow up: 6 months postpartum; assessed with: Chinese Kansas Marital Satisfaction Scale (CKMSS); Scale from: 3 to 21 (more satisfied)) | | | | | | | | | | | | |
| 1  (Leung) | RCT | very serious ^a,b^ | not serious | not serious | serious ^c^ | none | 231 | 231 | MD **0.47 higher** (0.09 lower to 1.03 higher) | SMD **0.15 higher**  (0.03 lower to 0.34 higher) | ⨁◯◯◯ VERY LOW | IMPORTANT |

**CI:** Confidence interval; **GHQ**: General Health Questionnaire; **MD:** Mean difference; **RCT**: Randomized Controlled Trial; **SMD**: Standardized Mean difference

**Explanations**

a. Questionnaire was completed by participants.

b. Selective reporting identified. One of the primary outcomes listed in clinicaltrials.gov was listed as a secondary outcome in the publication. The primary outcome reported as such was statistically significant, and the other was not statistically significant.

c. Due to uncertainty and lack of empirical evidence to support, we have elected not to calculate an OIS. Further, GRADE suggests a rule of thumb of a minimum of 800 participants for continuous outcomes. As there were 462 participants in this study and without a known threshold, a judgement of serious concern was applied.

**Infant outcomes**

| **Certainty assessment** | | | | | | | **№ of patients** | | **Effect** | | **Certainty** | **Importance** |
| --- | --- | --- | --- | --- | --- | --- | --- | --- | --- | --- | --- | --- |
| **№ of studies** | **Study design** | **Risk of bias** | **Inconsistency** | **Indirectness** | **Imprecision** | **Other considerations** | **Screening** | **No screening** | **Absolute (95% CI)**  **[MD]** | **Absolute**  **(95% CI)**  **[SMD]** |  |  |
| Child body weight (in kg) (follow up: 6 months postpartum; assessed with: Maternal and Child Health Centres records) | | | | | | | | | | | | |
| 1  (Leung) | RCT | serious ^b^ | not serious | not serious | serious ^c^ | none | 231 | 231 | MD **0.05 higher** (0.10 lower to 0.20 higher) | SMD **0.06 higher**  (0.12 lower to 0.24 higher) | ⨁⨁◯◯ LOW | IMPORTANT |
| Child number of hospitalization (follow up: 6 months postpartum; assessed with: reported by mothers or caregivers) | | | | | | | | | | | | |
| 1  (Leung) | RCT | very serious ^b,e^ | not serious | not serious | serious ^c^ | none | 231 | 231 | MD **0.04 higher** (0.09 lower to 0.17 higher) | SMD **0.06 higher**  (0.13 lower to 0.24 higher) | ⨁◯◯◯ VERY LOW | IMPORTANT |

**CI:** Confidence interval; **MD:** Mean difference; **RCT**: Randomized Controlled Trial; **SMD**: Standardized Mean difference

**Explanations**

b. Selective reporting identified. One of the primary outcomes listed in clinicaltrials.gov was listed as a secondary outcome in the publication. The primary outcome reported as such was statistically significant, and the other was not statistically significant.

c. Due to uncertainty and lack of empirical evidence to support, we have elected not to calculate an OIS. Further, GRADE suggests a rule of thumb of a minimum of 800 participants for continuous outcomes. As there were 462 participants in this study and without a known threshold, a judgement of serious concern was applied.

e. Reported by mothers/caregivers.

### Additional file 6.6. Postpartum summary of findings table

| **Screening compared to no screening for pregnant and postpartum depression** | | | | | | |
| --- | --- | --- | --- | --- | --- | --- |
| **Patient or population**: postpartum women  **Setting**: Maternal and Child Health Centres  **Intervention**: Screening  **Comparison**: no screening | | | | | | |
| Outcomes | **Anticipated absolute effects^*^** (95% CI) | | Relative effect (95% CI) | № of participants  (studies) | Certainty of the evidence (GRADE) | Comments |
|  | **Risk with no screening** | **Risk with Screening** |  |  |  |  |
| Number identified as depressed (EPDS ≥ 10) assessed with: EPDS follow up: 6 months postpartum | 221 per 1,000 | **130 per 1,000** (86 to 196) | **RR 0.59** (0.39 to 0.89) | 462 (1 RCT) ^1^ | ⨁◯◯◯ VERY LOW ^a,b,c^ |  |
| EPDS score assessed with: EPDS Scale from: 0 to 30 (worse) follow up: 6 m months postpartum | The mean EPDS score was **6.50** | The mean EPDS score in the intervention group was 1.36 lower (2.09 lower to 0.63 lower) | - | 462 (1 RCT) ^1^ | ⨁◯◯◯ VERY LOW ^a,b,c^ |  |
| GHQ score assessed with: GHQ-12 Scale from: 0 to 12 (more severe) follow up: 6 months postpartum | The mean GHQ score was **1.39** | The mean GHQ score in the intervention group was 0.33 lower (0.70 lower to 0.04 higher) | - | 462 (1 RCT) ^1^ | ⨁◯◯◯ VERY LOW ^a,b,c^ |  |
| Harms of treatment: Adverse events  follow up: 6 months postpartum | Not estimable | Not estimable | Not estimable | 462  (1 RCT) ^1^ | ⨁◯◯◯ VERY LOW ^b,c,d^ | It is unclear how data on adverse events was collected or reported. Reported only as “No adverse events were  reported throughout the study” |
| Parenting Stress Index - Total score assessed with: PSI-SF Scale from: 36 to 180 (more stress) follow up: 6 months postpartum | The mean parenting Stress Index - Total score was **83.67** | The mean parenting Stress Index - Total score in the intervention group was 2.78 lower (5.74 lower to 0.18 higher) | - | 462 (1 RCT) ^1^ | ⨁◯◯◯ VERY LOW ^a,b,c^ |  |
| PSI-Parental distress subscale score assessed with: PSI-SF PD subscale Scale from: 12 to 60 (more stress) follow up: 6 months postpartum | The mean PSI-Parental distress subscale score was **31.14** | The mean PSI-Parental distress subscale score in the intervention group was 1.21 lower (2.48 lower to 0.06 higher) | - | 462 (1 RCT) ^1^ | ⨁◯◯◯ VERY LOW ^a,b,c^ |  |
| PSI-Parent-child dysfunctional interaction subscale score assessed with: PSI-SF PCDI subscale Scale from: 12 to 60 (more stress) follow up: 6 months postpartum | The mean PSI-Parent-child dysfunctional interaction subscale score was **25.85** | The mean PSI-Parent-child dysfunctional interaction subscale score in the intervention group was 1.08 lower (2.17 lower to 0.01 higher) | - | 462 (1 RCT) ^1^ | ⨁◯◯◯ VERY LOW ^a,b,c^ |  |
| PSI-Difficult child subscale score assessed with: PSI-SF DC subscale Scale from: 12 to 60 (more stress) follow up: 6 months postpartum | The mean PSI-Difficult child subscale score was **26.68** | The mean PSI-Difficult child subscale score in the intervention group was 0.49 lower (1.64 lower to 0.66 higher) | - | 462 (1 RCT) ^1^ | ⨁◯◯◯ VERY LOW ^a,b,c^ |  |
| Marital satisfaction score (CKMSS) assessed with: Chinese Kansas Marital Satisfaction Scale Scale from: 3 to 21 (more satisfied) follow up: 6 months postpartum | The mean marital satisfaction score was **16.47** | The mean marital satisfaction score in the intervention group was 0.47 higher (0.09 lower to 1.03 higher) | - | 462 (1 RCT) ^1^ | ⨁◯◯◯ VERY LOW ^a,b,c^ |  |
| Child body weight (in kg) assessed with: Maternal and Child Health Centres records follow up: 6 months postpartum | The mean child body weight (in kg) was **7.66** | The mean child body weight (in kg) in the intervention group was 0.05 higher (0.10 lower to 0.20 higher) | - | 462 (1 RCT) ^1^ | ⨁⨁◯◯ LOW ^b,c^ |  |
| Child number of hospitalizations assessed with: Mothers or caregivers follow up: 6 months postpartum | The mean child number of hospitalizations was **0.37** | The mean child number of hospitalizations in the intervention group was 0.04 higher (0.09 lower to 0.17 higher) | - | 462  (1 RCT) ^1^ | ⨁◯◯◯ VERY LOW ^b,c,e^ |  |
| ***The risk in the intervention group** (and its 95% confidence interval) is based on the assumed risk in the comparison group and the **relative effect** of the intervention (and its 95% CI).  **CI:** Confidence interval; **RR:** Risk ratio; **MD:** Mean difference; **PSI**: Parenting Stress Index | | | | | | |
| **GRADE Working Group grades of evidence** **High certainty:** We are very confident that the true effect lies close to that of the estimate of the effect **Moderate certainty:** We are moderately confident in the effect estimate: The true effect is likely to be close to the estimate of the effect, but there is a possibility that it is substantially different **Low certainty:** Our confidence in the effect estimate is limited: The true effect may be substantially different from the estimate of the effect **Very low certainty:** We have very little confidence in the effect estimate: The true effect is likely to be substantially different from the estimate of effect | | | | | | |

**Explanations**

a. Questionnaire was completed by participants.

b. Selective reporting identified. One of the primary outcomes listed in clinicaltrials.gov was listed as a secondary outcome in the publication. The primary outcome reported as such was statistically significant, and the other was not statistically significant.

c. Due to uncertainty and lack of empirical evidence to support, we have elected not to calculate an OIS. Further, GRADE suggests a rule of thumb of a minimum of 400 events for dichotomous outcomes and 800 participants for continuous outcomes. As there were 462 participants in this study and without a known threshold, a judgement of serious concern was applied.

d. No information on how this information was collected, so many domains were unclear.

e. Reported by mothers/caregivers.

**References**

1. Leung, S.S., Leung, C., Lam, T.H., Hung, S.F., Chan, R., Yeung, T., Miao, M., Cheng, S., Leung, S.H., Lau, A. and Lee, D.T., 2011. Outcome of a postnatal depression screening programme using the Edinburgh Postnatal Depression Scale: a randomized controlled trial. Journal of Public Health, 33(2), pp.292-301.

## Additional file 7. General adult population excluded studies bibliography with reason

**Full-text not available (n=3)**

1. Felice E, Agius A, Sultana R, Felice EM, Calleja-Agius J. The effectiveness of psychosocial assessment in the detection and management of postpartum depression: a systematic review. Minerva Ginecologica 2018; 70(3):323-345.
2. Beglinger LJ, Adams WH, Langbehn D, Fiedorowicz JG, Caviness J, Biglan K, Olson B, Paulsen JS. Does interval between screening and baseline matter in HD cognitive clinical trials? Journal of Huntington's Disease 2014; 3(2):139-44.
3. Anonymous. Canadian Stroke Conference 2015. International Journal of Stroke 2015; 10 (SUPPL. 4).

**Not English or French (n=2)**

1. Zhan G-L, Li C-H, Zhao L-Y, Li J, Wu Y. Effects of community mental health services on depression, anxiety, and happiness of the elderly. Journal of Shanghai Jiaotong University (Medical Science) 2015; 35(6):839-842. [Chinese]
2. Zhan C-Y, Shi J-Y, Mao Z-Y, Zhao X-D. Perceived stress and coping style in college students with psychosis-risk syndrome. Chinese Mental Health Journal 2017; 31(8):614-618. [Chinese]

**Systematic reviews/Guidelines (n=17)**

1. Keshavarz H, Fitzpatrick-Lewis D, Streiner DL, Rice M, Ali U, Shannon HS, Raina P. Screening for depression: a systematic review and meta-analysis. CMAJ Open 2013; 1(4):E159-67.
2. Okolie C, Dennis M, Simon Thomas E, John A. A systematic review of interventions to prevent suicidal behaviors and reduce suicidal ideation in older people. International Psychogeriatrics 2017; 29(11):1801-1824.
3. Richardson R, Trepel D, Perry A, Ali S, Duffy S, Gabe R, Gilbody S, Glanville J, Hewitt C, Manea L, Palmer S, Wright B, McMillan D. Screening for psychological and mental health difficulties in young people who offend: a systematic review and decision model. Health technology assessment (Winchester, England) 2015; 19(1):1-128.
4. Martin MS, Colman I, Simpson AIF, McKenzie K. Mental health screening tools in correctional institutions: A systematic review. BMC Psychiatry 2013; 13:275.
5. Joffres M, Jaramillo A, Dickinson J, Lewin G, Pottie K, Shaw E, Gorber SC, Tonelli M. Recommendations on screening for depression in adults. CMAJ 2013; 185(9):775-782.
6. Gilbody S, House A, Sheldon T. Screening and case finding instruments for depression. CMAJ 2008; 178(8):997-1003.
7. Austin MP, Priest SR, Sullivan EA. Antenatal psychosocial assessment for reducing perinatal mental health morbidity. Cochrane Database of Systematic Reviews 2008; 4.
8. [Grey Lit] CADTH Rapid Response Report. Diagnosis, Screening and Monitoring Depression in the Elderly: A Review of Guidelines. 8 September 2015.
9. [Grey Lit] Keshavarz H, Fitzpatrick-Lewis D, Streiner D, Rice M, Raina P. Screening for Depression. November 2012.
10. Baker JM, Grant RW, Gopalan A. A systematic review of care management interventions targeting multimorbidity and high care utilization. BMC Health Services Research 2018; 18(1):65.
11. Fleming N, O'Driscoll T, Becker G, Spitzer RF, CANPAGO COMMITTEE. Adolescent Pregnancy Guidelines. Journal of Obstetrics and Gynaecology Canada 2015; 37(8):740-756.
12. Byatt N, Levin LL, Ziedonis D, Moore Simas TA, Allison J. Enhancing Participation in Depression Care in Outpatient Perinatal Care Settings: A Systematic Review. Obstetrics and Gynecology 2015; 126(5):1048-58.
13. Thombs BD, Arthurs E, Coronado-Montoya S, Roseman M, Delisle VC, Leavens A, Levis B, Azoulay L, Smith C, Ciofani L, Coyne JC, Feeley N, Gilbody S, Schinazi J, Stewart DE, Zelkowitz P. Depression screening and patient outcomes in pregnancy or postpartum: a systematic review. Journal of Psychosomatic research 2014; 76(6):433-46.
14. Thombs BD, Ziegelstein RC, Roseman M, Kloda LA, Ioannidis JPA. There are no randomized controlled trials that support the United States Preventive Services Task Force Guideline on screening for depression in primary care: a systematic review. BMC Medicine 2014; 12:13.
15. Health Quality Ontario. Screening and management of depression for adults with chronic diseases: an evidence-based analysis. Ontario Health Technology Assessment Series 2013; 13(8):1-45.
16. Conejo-Ceron S, Moreno-Peral P, Rodriguez-Morejon A, Motrico E, Navas-Campana D, Rigabert A, Martin-Perez C, Rodriguez-Bayon A, Ballesta-Rodriguez MI, de Dios Luna J, Garcia-Campayo J, Roca M, Bellon JA. Effectiveness of psychological and educational interventions to prevent depression in primary care: A systematic review and meta-analysis. Annals of Family Medicine 2017; 15(3):262-271.
17. [Grey Lit] Nieuwsma JA, Williams JW, Namdari N, Washam JB, Raitz G, Blumenthal JA, Jiang W, Yapa R, McBroom AJ, Lallinger K, Schmidt R, Kosinski AS, Sanders GD. Diagnostic Accuracy of Screening Tests and Treatment for Post-Acute Coronary Sydnrome Depression. A Systematic Review. Annals of Internal Medicine 2017; 167:725-735.

**Observational studies (e.g., cohorts, case-controls, cross-sectionals, case series) (n=46)**

1. [Grey Lit] Eng K, Jones A, Feeny D. Missing Data and Methods for Imputing Missing Depression Scores: Experience in a Study of Hip Fracture Patients. Working Paper 05-03. Legal Deposit 2000. National Library of Canada. ISSN 1481-3823. ISBN 978-1-926929-57-6 (online) October 2005.
2. [Grey Lit] Gonzalez OI, Novaco RW, Reger MA, Gahm GA. Anger Intensification With Combat-Related PTSD and Depression Comorbidity. Psychological Trauma: Theory, Research, Practice, and Policy 2016; 8(1):9-16.
3. [Grey Lit] Nadeau MM, Balsan MJ, Rochlen AB. Men’s Depression: Endorsed Experiences and Expressions. Psychology of Men & Masculinity 2016; 17(4):328-335.
4. Akena D, Stein DJ, Joska J. Does screening HIV-positive individuals in Uganda for major depressive disorder improve case detection rates and antidepressant prescription? AIDS and Behavior 2013; 17(8):2802-7.
5. Alkhadhari S, Alsabbrri OA, Mohammad IHA, Atwan AA, Alqudaihi F, Zahid MA. Prevalence of psychiatric morbidity in the primary health clinic attendees in Kuwait. Journal of Affective Disorders 2016; 195:15-20.
6. Barnett LA, Lewis M, Mallen CD, Peat G. Applying quantitative bias analysis to estimate the plausible effects of selection bias in a cluster randomised controlled trial: secondary analysis of the Primary care Osteoarthritis Screening Trial (POST). Trials 2017; 18(1):585.
7. Bekelman DB, Hooker S, Nowels CT, Main DS, Meek P, McBryde C, Hattler B, Lorenz KA, Heidenreich PA. Feasibility and acceptability of a collaborative care intervention to improve symptoms and quality of life in chronic heart failure: mixed methods pilot trial. Journal of Palliative Medicine 2014; 17(2):145-51.
8. Belsher BE, Evatt DP, Liu X, Freed MC, Engel CC, Beech EH, Jaycox LH. Collaborative Care for Depression and Posttraumatic Stress Disorder: Evaluation of Collaborative Care Fidelity on Symptom Trajectories and Outcomes. Journal of General Internal Medicine 2018; 33(7):1124-1130.
9. Chi CI. The early screening and intervention mode research of patients with depression and anxiety disorders in Guangzhou general hospitals. http://wwwwhoint/trialsearch/Trial2aspx?TrialID=ChiCTR-INR-16008066. 2016.
10. Chilcot J, Almond MK, Guirguis A, Friedli K, Day C, Davenport A, Wellsted D, Farrington K. Self-reported depression symptoms in haemodialysis patients: Bi-factor structures of two common measures and their association with clinical factors. General Hospital Psychiatry 2018; 54:31-36.
11. Devraj R, Singh VB, Ola V, Meena BL, Tundwal V, Singh K. Cognitive function in elderly population - An urban community based study in north-west Rajasthan. Journal, Indian Academy of Clinical Medicine 2014; 15(2):87-90.
12. Djukanovic I, Carlsson J, Arestedt K. Is the Hospital Anxiety and Depression Scale (HADS) a valid measure in a general population 65-80 years old? A psychometric evaluation study. Health and Quality of Life Outcomes 2017; 15(1):193.
13. Drummond KL, Painter JT, Curran GM, Stanley R, Gifford AL, Rodriguez-Barradas M, Rimland D, Monson TP, Pyne JM. HIV patient and provider feedback on a telehealth collaborative care for depression intervention. AIDS Care 2017; 29(3):290-298.
14. Du N, Yu K, Ye Y, Chen S. Validity study of Patient Health Questionnaire-9 items for Internet screening in depression among Chinese university students. Asia-Pacific psychiatry 2017; 9(3):e12266.
15. Englbrecht M, Alten R, Aringer M, Baerwald CG, Burkhardt H, Eby N, Fliedner G, Gauger B, Henkemeier U, Hofmann MW, Kleinert S, Kneitz C, Krueger K, Pohl C, Roske A-E, Schett G, Schmalzing M, Tausche A-K, Tony HP, Wendler J. Validation of Standardized Questionnaires Evaluating Symptoms of Depression in Rheumatoid Arthritis Patients: approaches to Screening for a Frequent Yet Underrated Challenge. Arthritis Care & Research 2017; 69(1):58‐66.
16. Frost J, Rich RL, Robbins CW, et al. Depression following acute coronary syndrome events: Screening and treatment guidelines from the AAFP. American Family Physician. 2019;99(12):786A-786J.
17. Gonzalez-Blanch C, Medrano LA, Munoz-Navarro R, Ruiz-Rodriguez P, Moriana JA, Limonero JT, Schmitz F, Cano-Vindel A, PsicAP Research Group. Factor structure and measurement invariance across various demographic groups and over time for the PHQ-9 in primary care patients in Spain. PloS One 2018; 13(2):e0193356.
18. Green KL, Brown GK, Jager-Hyman S, Cha J, Steer RA, Beck AT. The Predictive Validity of the Beck Depression Inventory Suicide Item. The Journal of Clinical Psychiatry 2015; 76(12):1683-6.
19. Han C, Voils CI, Williams JW Jr. Uptake of web-based clinical resources from the MacArthur Foundation's Depression and Primary Care. Community Mental Health Journal 2013; 49(2):166-171.
20. Hauffman A, Alfonsson S, Igelström H, Johansson B. Experiences of Internet-Based Stepped Care in Individuals With Cancer and Concurrent Symptoms of Anxiety and Depression: Qualitative Exploration Conducted Alongside the U-CARE AdultCan Randomized Controlled Trial. J Med Internet Res. 2020 Mar 30;22(3):e16547. doi: 10.2196/16547.
21. Ibrahim H, Ertl V, Catani C, Ismail AA, Neuner F. The validity of Posttraumatic Stress Disorder Checklist for DSM-5 (PCL-5) as screening instrument with Kurdish and Arab displaced populations living in the Kurdistan region of Iraq. BMC Psychiatry. 2018;18.
22. Implementation of comprehensive rehabilitation therapy in postoperative care of patients with cholangiocarcinoma and its impact on patients' quality of life. Experimental and therapeutic medicine. 2019;17(4):2703‐2707.
23. Inoguchi H, Shimizu K, Shimoda H, Yoshiuchi K, Akechi T, Uchida M, Ogawa A, Fujisawa D, Inoue S, Uchitomi Y. Screening for untreated depression in cancer patients: a Japanese experience. Japanese Journal of Clinical Oncology 2016; 46(11):993‐999.
24. Jamal-Omidi S, Collins C, Fulchiero E, Liu H, Colon-Zimmermann K, Fuentes-Casiano E, Tatsuoka C, Cassidy KA, Lhatoo S, Sajatovic M. Assessing depression severity with a self-rated vs. rater-administered instrument in patients with epilepsy. Epilepsy & Behavior: E&B 2018; 85:52-57.
25. Jarbin H, Ivarsson T, Andersson M, Bergman H, Skarphedinsson G. Screening efficiency of the Mood and Feelings Questionnaire (MFQ) and Short Mood and Feelings Questionnaire (SMFQ) in Swedish help seeking outpatients. PLoS ONE. 2020;15(3):e0230623.
26. Kigozi J, Jowett S, Nicholl BI, et al. Cost-Utility Analysis of Routine Anxiety and Depression Screening in Patients Consulting for Osteoarthritis: Results From a Clinical, Randomized Controlled Trial. Arthritis Care and Research. 2018;70(12):1787-1794.
27. Kim JM, Stewart R, Kang HJ, et al. Long-term cardiac outcomes of depression screening, diagnosis and treatment in patients with acute coronary syndrome: the DEPACS study. Psychological medicine. 2020:1-11.
28. Kubota C, Inada T, Nakamura Y, et al. Stable factor structure of the Edinburgh Postnatal Depression Scale during the whole peripartum period: Results from a Japanese prospective cohort study. Scientific Reports. 2018;8(1):17659.
29. Lazenby M, Dixon J, Bai M, McCorkle R. Comparing the distress thermometer (DT) with the patient health questionnaire (PHQ)-2 for screening for possible cases of depression among patients newly diagnosed with advanced cancer. Palliative & Supportive Care 2014; 12(1):63-8.
30. McKean M, Caughey AB, Yuracko McKean MA, Cabana MD, Flaherman VJ. Postpartum Depression: When Should Health Care Providers Identify Those at Risk? Clinical Pediatrics 2018; 57(6):689-693.
31. Morys JM, Bellwon J, Adamczyk K, Gruchala M. Depression and anxiety in patients with coronary artery disease, measured by means of self-report measures and clinician-rated instrument. Kardiologia Polska 2016; 74(1):53‐60.
32. Ofori SN, Adiukwu FN. Screening for Depressive Symptoms among Patients Attending Specialist Medical Outpatient Clinics in a Tertiary Hospital in Southern Nigeria. psychiatry j. 2018;2018:7603580.
33. Ono Y, Sakai A, Otsuka K, Uda H, Oyama H, Ishizuka N, Awata S, Ishida Y, Iwasa H, Kamei Y, Motohashi Y, Nakamura J, Nishi N, Watanabe N, Yotsumoto T, Nakagawa A, Suzuki Y, Tajima M, Tanaka E, Sakai H, Yonemoto N. Effectiveness of a multimodal community intervention program to prevent suicide and suicide attempts: a quasi-experimental study. PloS One 2013; 8(10):e74902.
34. Oyama H, Sakashita T. Community-based screening intervention for depression affects suicide rates among middle-aged Japanese adults. Psychological Medicine 2017; 47(8):1500‐1509.
35. Oyama H, Sakashita T. Differences in specific depressive symptoms among community-dwelling middle-aged Japanese adults before and after a universal screening intervention. Social Psychiatry and Psychiatric Epidemiology 2014; 49(2):251-8.
36. Oyama H, Sakashita T. Effects of universal screening for depression among middle-aged adults in a community with a high suicide rate. The Journal of Nervous and Mental Disease 2014; 202(4):280-6.
37. Salgado PCB, Nogueira MH, Yasuda CL, Cendes F. Screening symptoms of depression and suicidal ideation in people with epilepsy using the Beck depression inventory. Journal of Epilepsy and Clinical Neurophysiology 2012; 18(3):85-91.
38. Shah A, Scogin F, Pierpaoli CM, Shah A. Older adults' attitudes toward depression screening in primary care settings and exploring a brief educational pamphlet. International Journal of Geriatric Psychiatry 2018; 33(1):e40-e48.
39. Shaw RJ, Lilo EA, Storfer-Isser A, Ball MB, Proud MS, Vierhaus NS, Huntsberry A, Mitchell K, Adams MM, Horwitz SM. Screening for symptoms of postpartum traumatic stress in a sample of mothers with preterm infants. Issues in mental health nursing 2014; 35(3):198-207.
40. Topitzes J, Mersky JP, Mueller DJ, Bacalso E, Williams C. Implementing Trauma Screening, Brief Intervention, and Referral to Treatment (T-SBIRT) within Employment Services: A Feasibility Trial. Am J Community Psychol. 2019;64(3-4):298-309.
41. van Nuenen FM, Donofrio SM, Tuinman MA, van de Wiel HBM, Hoekstra-Weebers JEHM. Effects on patient-reported outcomes of "Screening of Distress and Referral Need" implemented in Dutch oncology practice. Supportive Care in Cancer. 2019.
42. Wang H, Pan Y, Guo C, Li F, Xu R, Liu M, Liu Z, Liu F, Cai H, Ke Y, He Z. Health-related quality of life among rural residents aged 45-69 years in Hua County, Henan Province, China: Results of ESECC Trial for esophageal cancer screening with endoscopy. Chinese Journal of Cancer Research 2018; 30(2):240-253.
43. Wikberg C, Nejati S, Larsson MEH, Petersson E-L, Westman J, Ariai N, Kivi M, Eriksson M, Eggertsen R, Hange D, Baigi A, Bjorkelund C. Comparison between the montgomery-asberg depression rating scale-self and the beck depression inventory II in primary care. Primary Care Companion to the Journal of Clinical Psychiatry 2015; 17(3):10.4088/PCC.14m01758.
44. Wikberg C, Pettersson A, Westman J, Björkelund C, Petersson EL. Patients' perspectives on the use of the Montgomery-Asberg depression rating scale self-assessment version in primary care. Scandinavian Journal of Primary Health Care 2016; 34(4):434‐442.
45. Wu C-Y, Lee J-I, Lee M-B,Liao S-C, Chang C-M, Chen H-C, Lung F-W. Predictive validity of a five-item symptom checklist to screen psychiatric morbidity and suicide ideation in general population and psychiatric settings. Journal of the Formosan Medical Association 2016; 115(6):395-403.
46. Yawn BP, Bertram S, Kurland M, Wollan PC. Repeated depression screening during the first postpartum year. Annals of Family Medicine 2015; 13(3):228-34.

**Narrative/literature reviews (non-systematic) (n=15)**

1. [Grey Lit] Bartha C, Parker C, Thomson C, Kitchen K. Depression An information Guide. Revised Edition. Centre for Addiction and Mental Health. 2013.
2. [Grey Lit] Holtzheimer PE, Roy-Byrne PP, Solomon D. Depression in adults: Overview of neuromodulation procedures. UpToDate 2018, accessed October 2018.
3. [Grey Lit] Mӧller HJ, Henkel V. What are the most effective diagnostic and therapeutic strategies for the management of depression in specialist care? 2005 Copenhagen, WHO Regional Office for Europe (Health Evidence Network report; <http://www.euro.who.int/Document/E86602.pdg>, accessed October 2018])
4. [Grey Lit] Savoy M, O’Gurek D. Screening Your Adult Patients for Depression. 2016 Family Practice Management website ([www.aafp.org/fpm](http://www.aafp.org/fpm)), accessed October 2018.
5. [Grey Lit]. Maurer DM. Screening for Depression. 2012 Family Practice Management website ([www.aafp.org/fpm](http://www.aafp.org/fpm)), accessed October 2018.
6. Alagiakrishnan K, Sclater A. Psychiatric disorders presenting in the elderly with type 2 diabetes mellitus. American Journal of Geriatric Psychiatry 2012; 20(8):645-652.
7. Baumgartner C, Blum MR, Rodondi N. Subclinical hypothyroidism: summary of evidence in 2014. Swiss medical weekly 2014; 144:w14058.
8. Clare CA, Yeh J. Postpartum depression in special populations: A review. Obstetrical and Gynecological Survey 2012; 67(5):313-323.
9. Craven MA, Bland R. Depression in primary care: current and future challenges. Canadian Journal of Psychiatry. 2013; 58(8):442-8.
10. Duerksen A, Dubey V, Iglar K. Annual adult health checkup: Update on the preventive care checklist form©. Canadian Family Physician 2012; 58(1):43-e20.
11. Goodarzi Z, Ismail Z. A practical approach to detection and treatment of depression in Parkinson disease and dementia. Neurology: Clinical Practice 2017; 7(2):128-140.
12. Hansotte E, Payne SI, Babich SM. Positive postpartum depression screening practices and subsequent mental health treatment for low-income women in Western countries: A systematic literature review. Public Health Reviews 2017; 38(1):3.
13. Langan RC, Goodbred AJ. Identification and management of peripartum depression. American Family Physician 2016; 93(10):852-858.
14. Reilly N, Kingston D, Loxton D, Talcevska K, Austin MP. A narrative review of studies addressing the clinical effectiveness of perinatal depression screening programs. Women Birth. 2020;33(1):51-59.
15. Wilkin T. Primary care for men who have sex with men. New England Journal of Medicine 2015; 373(9):854-862.

**Protocol (n=96)**

1. Biegler K, Mollica R, Elliott Sim S, Elisa Nicholas, Maria Chandler, Quyen Ngo-Metzger, Kittya Paigne, Sompia Paigne, Danh V Nguyen, Dara H Sorkin. Rationale and study protocol for a multi-component Health Information Technology (HIT) screening tool for depression and post-traumatic stress disorder in the primary care setting. Contemporary clinical trials 2016; 50:66-76.
2. Gladstone TG, Marko-Holguin M, Rothberg P, Nidetz J, Diehl A, DeFrino DT, Harris M, Ching E, Eder M, Canel J, Bell C, Beardslee WR, Hendricks Brown C, Griffiths K, Van Voorhees BW. An internet-based adolescent depression preventive intervention: study protocol for a randomized control trial. Trials 2015; 16:203.
3. Kingston D, Austin M-P, Hegadoren K, Lasiuk G, McDonald S, Heaman M, Biringer A, Sword W, Giallo R, Patel T, Lane-Smith M, van Zanten SV. Study protocol for a randomized, controlled, superiority trial comparing the clinical and cost- effectiveness of integrated online mental health assessment-referral-care in pregnancy to usual prenatal care on prenatal and postnatal mental health and infant health and development: The Integrated Maternal Psychosocial Assessment to Care Trial (IMPACT). Trials 2014; 15(1):72.
4. Kingston D, McDonald S, Biringer A, Austin M-P, Hegadoren K, McDonald S, Giallo R, Ohinmaa A, Lasiuk G, MacQueen G, Sword W, Lane-Smith M, Veldhuyzen van Zanten S. Comparing the feasibility, acceptability, clinical-, and cost-effectiveness of mental health e-screening to paper-based screening on the detection of depression, anxiety, and psychosocial risk in pregnant women: a study protocol of a randomized, parallel-group, superiority trial. Trials 2014; 15:3.
5. Marcano Belisario JS, Doherty K, O'Donoghue J, Ramchandani P, Majeed A, Doherty G, Morrison C, Car J. A bespoke mobile application for the longitudinal assessment of depression and mood during pregnancy: protocol of a feasibility study. BMJ Open 2017; 7(5):e014469.
6. Marcano-Belisario JS, Gupta AK, O'Donoghue J, Morrison C, Car J. Tablet computers for implementing NICE antenatal mental health guidelines: protocol of a feasibility study. BMJ Open 2016; 6(1):e009930.
7. McKenzie JE, French SD, O'Connor DA, Mortimer DS, Browning CJ, Russell GM, Grimshaw JM, Eccles MP, Francis JJ, Michie S, Murphy K, Kossenas F, Green SE, IRIS trial group. Evidence-based care of older people with suspected cognitive impairment in general practice: protocol for the IRIS cluster randomised trial. Implementation science 2013; 8:91.
8. Rawther SCH, Shivananda Pai M, Fernandes DJ, Mathew S, Binu VS, Chakrabarty J, Sanatombi Devi E, George A, Nayak BS. A Randomized controlled trial to evaluate the impact of a Nurse Navigator Programme on outcomes of people with breast cancer: study protocol. Journal of Advanced Nursing 2017; 73(4):977-988.
9. Schuurhuizen CS, Braamse AM, Beekman AT, Bomhof-Roordink H, Bosmans JE, Cuijpers P, Hoogendoorn AW, Konings IR, van der Linden MH, Neefjes EC, Verheul HMW, Deller J. Screening and treatment of psychological distress in patients with metastatic colorectal cancer: study protocol of the TES trial. BMC Cancer 2015; 15:302.
10. Singer S, Danker H, Briest S, Dietrich A, Dietz A, Einenkel J, Papsdorf K, Lordick F, Meixensberger J, Mossner J, Niederwieser D, Prietzel T, Schiefke F, Stolzenburg J-U, Wirtz H, Kersting A. Effect of a structured psycho-oncological screening and treatment model on mental health in cancer patients (STEPPED CARE): Study protocol for a cluster randomized controlled trial. Trials 2014; 15(1):482.
11. Tait L, Michail M. Educational interventions for general practitioners to identify and manage depression as a suicide risk factor in young people: a systematic review and meta-analysis protocol. Systematic Reviews 2014; 3:145.
12. Tancredi DJ, Slee CK, Jerant A, Franks P, Nettiksimmons J, Cipri C, Gottfeld D, Huerta J, Feldman MD, Jackson-Triche M, Kelly-Reif S, Hudnut A, Olson S, Shelton J, Kravitz RL. Targeted versus tailored multimedia patient engagement to enhance depression recognition and treatment in primary care: randomized controlled trial protocol for the AMEP2 study. BMC Health Services Research 2013; 13:141.
13. Terrazas C, Segre LS, Wolfe C. Moving beyond depression screening: integrating perinatal depression treatment into OB/GYN practices. Primary Health Care Research & Development 2018:1-9.
14. Kirkham JG, Takwoingi Y, Quinn TJ, Rapoport M, Lanctôt KL, Maxwell CJ, Herrmann N, Gill SS, Rochon PA, Seitz DP. Depression rating scales for detection of major depression in people with dementia. Cochrane Database of Systematic Reviews 2016; (8).
15. NCT01993017. Comparison of Depression Identification After Acute Coronary Syndrome: quality of Life and Cost Outcomes.
16. Eli Lilly Company. Effectiveness of Physicians' Education, Implementation and Follow up of Current Recommendations Regarding Screening for Major Depressive Disorder in High Risk Patients on Improving the Under-Recognition Rates of Depression in Primary Care. 2012.
17. NCT03191929. Depression Screening in Primary Care: using HIT for Patients With Limited English. 2017.
18. NCT01773629. Care Managers for Perinatal Depression (CMPD). 2013.
19. NCT02468466. A Community-based Depression Screening Intervention for Middle-aged Suicide. 2015.
20. NCT03534167. TODAY! A Mobile App Study. 2018.
21. R Gray. DEPRET-STROKE An exploratory randomised controlled trial of a DEPression Recognition and Treatment package for families living with STROKE. 2014.
22. Loewe B. Increasing the Efficiency of Depression-screening Using Patient-targeted Feedback: randomized Controlled Trial. 2013.
23. Davidson KW. Depression Screening RCT in ACS Patients: quality of Life and Cost Outcomes. 2013.
24. NCT01885026. Substance-use Focused Screening and Brief Intervention as a Complement to Internet-based Psychiatric Treatment for Depression, Panic Disorder or Social Phobia: a Randomized Controlled Trial (eScreeniPsy). 2013.
25. NCT01626703. Effect of Depression Screening and Care Program at Community Health Center. 2012.
26. NCT02702596. METRIC: measurement, Education and Tracking in Integrated Care. 2016.
27. NCT01843907. Patient Participation in Prevention of Loss of Functions. 2013.
28. NCT03448289. Use of a Reproductive Life Planning Tool at the Pediatric Well-Baby Visit With Postpartum Women. 2018.
29. [Grey Lit] ISRCTN15886353. The effectiveness of a postnatal psychoeducation programme on outcomes of first-time mothers in Singapore. 2014. [10023]
30. [Grey Lit] ISRCTN12154418. Improving the care of people with long term conditions (ENHANCE). 2015. [10024]
31. [Grey Lit] NCT00251342. Outcome of Postnatal Depression Screening using Edinburgh Postnatal Depression Scale. 2005. [10025]
32. [Grey Lit] NCT00056901. Screening Evaluation for Women With Postpartum Depression. 2003. [10026]
33. [Grey Lit] NCT01238614. Universal Screening for Maternal Depression With the CHICA System. 2010. [10027]
34. [Grey Lit] NCT00548743. Translating Research Into Practice for Postpartum Depression (TRIPPD). 2007. [10028]
35. [Grey Lit] NCT00404365. Depression Screening in Patients with Lung Cancer. 2006. [10029]
36. [Grey Lit] NCT01326793. Internet-Based Depression Screening for College Students. 2011. [10030]
37. [Grey Lit] NCT03203395. Screening for Depression and Anxiety in Patients with Heart Disease. 2017. [10031]
38. [Grey Lit] NCT00430404. Screening and Treatment of Depression in the Community. 2007. [10032]
39. [Grey Lit] NCT01630681. Internet Based Screening and Stepped Care for Cancer Patients with Anxiety and Depression Symptoms (AdultCan). 2012. [10033]
40. [Grey Lit] NCT01899534. Mental Health E-screening in Pregnant and Postpartum Women. 2013. [10034]
41. [Grey Lit] NCT00433238. Screening for Mental Health Concerns for at-Risk Community Living Chinese Seniors. 2007. [10035]
42. [Grey Lit] NCT00515372. Depression Treatment and Screening in Ovarian Cancer Patients. 2007. [10036]
43. [Grey Lit] NCT00282776. Identification and Therapy of Postpartum Depression. 2006. [10037]
44. [Grey Lit] ANZCTR12616001217493. Integrated e-Screening for Postnatal Depression and Anxiety. 2016. [10038]
45. [Grey Lit] NCT02491034. DESEO: Depression Screening and Education: Options to Reduce Barriers to Treatment (DESEO). 2015. [10039]
46. [Grey Lit] ACTRN12606000483550. Randomised controlled trial of a New Zealand developed screening questionnaire for depression compared with a gold standard. 2016. [10041]
47. [Grey Lit] NCT03215199. Northern Alberta Psychosocial Telecare (NAPT) Screening for HNC patients. 2017. [10043]
48. [Grey Lit] ISRCTN49546767. Identifying depression in older patients in the emergency department. 2017. [10044]
49. [Grey Lit] ISRCTN42298046. The effectiveness of screening for postpartum depression in child health care. 2016. [10045]
50. [Grey Lit] ISRCTN95425657. TARGET: Targeted depression prevention program for at-risk adolescents. 2013. [10046]
51. [Grey Lit] ISRCTN8670765. Integrating depression management in HIV care in Uganda. [10047]
52. Isrctn. A randomised, controlled trial of a community-based perinatal intervention for postnatal depression in India. http://wwwwhoint/trialsearch/Trial2aspx?TrialID=ISRCTN56588156. 2002.
53. Jin H, Wu S. Text Messaging as a Screening Tool for Depression and Related Conditions in Underserved, Predominantly Minority Safety Net Primary Care Patients: Validity Study. Journal of Medical Internet Research. 2020;22(3):e17282.
54. Carey M, Sanson-Fisher R, Zwar N, et al. Improving depression outcomes among Australian primary care patients: protocol for a cluster randomised controlled trial. BMJ Open. 2020;10(2):e032057.
55. Wang J, MacQueen G, Patten S, Manuel D, Lashewicz B, Schmitz N. A randomized controlled trial to examine the impacts of disclosing personalized depression risk information on the outcomes of individuals who are at high risk of developing major depression: a research protocol. BMC Psychiatry. 2019;19(1):285.
56. Tesky VA, Schall A, Schulze U, et al. Depression in the nursing home: A cluster-randomized stepped-wedge study to probe the effectiveness of a novel case management approach to improve treatment (the DAVOS project). Trials. 2019;20 (1) (no pagination)(424).
57. Sekhar DL, Pattison KL, Confair A, et al. Effectiveness of Universal School-Based Screening vs Targeted Screening for Major Depressive Disorder Among Adolescents: A Trial Protocol for the Screening in High Schools to Identify, Evaluate, and Lower Depression (SHIELD) Randomized Clinical Trial. JAMA Network Open. 2019;2(11):e1914427-e1914427.
58. Satre DD, Anderson AN, Leibowitz AS, et al. Implementing electronic substance use disorder and depression and anxiety screening and behavioral interventions in primary care clinics serving people with HIV: Protocol for the Promoting Access to Care Engagement (PACE) trial. Contemporary Clinical Trials. 2019;84:105833.
59. Nct. Harnessing Mobile Technology to Reduce Mental Health Disorders in College Populations. https://clinicaltrialsgov/show/NCT04162847. 2019.
60. Nct. Enhancing the Clinical Effectiveness of Depression Screening Using Patient-targeted Feedback in General Practices: the GET.FEEDBACK.GP Multicentre Randomized Controlled Trial. https://clinicaltrialsgov/show/NCT03988985. 2019.
61. Nct. Implementation of Prevention and Intervention of Maternal Perinatal Depression to Strengthen Maternal and Child Health. https://clinicaltrialsgov/show/NCT04069091. 2019.
62. Nct. MAMA NO STRESS Project. The Effects of the "HAPPY MAMA" Intervention. https://clinicaltrialsgov/show/NCT04093791. 2019.
63. Nct. Psychological Support Based on Positive Suggestions (PSBPS) on Mental Health Morbidity and Cognitive Function. https://clinicaltrialsgov/show/NCT04045210. 2019.
64. Moore Simas TA, Brenckle L, Sankaran P, et al. The PRogram In Support of Moms (PRISM): study protocol for a cluster randomized controlled trial of two active interventions addressing perinatal depression in obstetric settings. BMC Pregnancy Childbirth. 2019;19(1):256.
65. Hamel C, Lang E, Morissette K, et al. Screening for depression in women during pregnancy or the first year postpartum and in the general adult population: a protocol for two systematic reviews to update a guideline of the Canadian Task Force on Preventive Health Care. Systematic Reviews. 2019;8(1):27.
66. Fletcher S, Chondros P, Palmer VJ, et al. Link-me: Protocol for a randomised controlled trial of a systematic approach to stepped mental health care in primary care. Contemporary Clinical Trials. 2019;78:63-75.
67. Carlin E, Blondell SJ, Cadet-James Y, et al. Study protocol: a clinical trial for improving mental health screening for Aboriginal and Torres Strait Islander pregnant women and mothers of young children using the Kimberley Mum's Mood Scale. BMC Public Health. 2019;19(1):1521.
68. Actrn. Perinatal Identification, Referral and Integrated Management for Improving Depression: the PIRIMID Study. http://wwwwhoint/trialsearch/Trial2aspx?TrialID=ACTRN12619001433190. 2019.
69. Pactr. COMPARISON OF ROUTINE VERSUS SELECTIVE SCREENING FOR DEPRESSION STRATEGIES AMONG PLHIV ATTENDING PRINCESS DIANA MEMORIAL HEALTH CENTRE IV SOROTI. http://wwwwhoint/trialsearch/Trial2aspx?TrialID=PACTR201802003141213. 2018.
70. Nct. "Prevention and Treatment of Depression in the Elderly: a Population-Based Study". https://clinicaltrialsgov/show/NCT03538873. 2018.
71. Nct. Evaluating a Mobile App for Students Seeking Care for Depression and Anxiety at Harvard University Health Services. https://clinicaltrialsgov/show/NCT03552900. 2018.
72. Nct. Universal vs. Targeted School Screening for Adolescent Major Depressive Disorder. https://clinicaltrialsgov/show/NCT03716869. 2018.
73. Butow P, Shaw J, Shepherd HL, et al. Comparison of implementation strategies to influence adherence to the clinical pathway for screening, assessment and management of anxiety and depression in adult cancer patients (ADAPT CP): study protocol of a cluster randomised controlled trial. BMC Cancer. 2018;18(1):1077.
74. Actrn. The post-operative review as an opportunity to intervene for postnatal depression in mothers undergoing Caesarean section. http://wwwwhoint/trialsearch/Trial2aspx?TrialID=ACTRN12618001577202. 2018.
75. Actrn. The ADAPT Program to support the management of anxiety and depression in adult cancer patients: a cluster randomised trial to evaluate different implementation strategies. http://wwwwhoint/trialsearch/Trial2aspx?TrialID=ACTRN12617000411347. 2017.
76. Nct. PRogram In Support of Moms: an Innovative Stepped-Care Approach for Obstetrics and Gynecology Clinics. https://clinicaltrialsgov/show/NCT02760004. 2016.
77. Ntr. Screening and treatment of psychological distress in colorectal cancer with metastasized disease: the TES-trial. http://wwwwhoint/trialsearch/Trial2aspx?TrialID=NTR4034. 2013.
78. Nct. Increasing the Efficiency of Depression-screening Using Patient-targeted Feedback. https://clinicaltrialsgov/show/NCT01879111. 2013.
79. Nct. Integrated Maternal Psychosocial Assessment to Care Trial (IMPACT) Full Trial. https://clinicaltrialsgov/show/NCT01901796. 2013.
80. Nct. Comparison Study of Family Practice Interventions for Depression in Adults. https://clinicaltrialsgov/show/NCT01975207. 2013.
81. Actrn. Cluster randomised controlled trial of a psycho-educational intervention for people with a family history of depression for use in general practice. http://wwwwhoint/trialsearch/Trial2aspx?TrialID=ACTRN12613000402741. 2013.
82. Nct. Major Depressive Disorder (MDD). https://clinicaltrialsgov/show/NCT01662817. 2012.
83. Isrctn. COPD in Primary Care Study. http://wwwwhoint/trialsearch/Trial2aspx?TrialID=ISRCTN49632858. 2012.
84. Drks. Increasing the efficiency of depression-screening using patient-targeted feedback: randomized controlled trial. http://wwwwhoint/trialsearch/Trial2aspx?TrialID=DRKS00003277. 2012.
85. Actrn. Evaluation of a nurse-led educational clinic for patients within 5-7 days post-discharge from hospital following percutaneous coronary intervention (PCI). http://wwwwhoint/trialsearch/Trial2aspx?TrialID=ACTRN12612000971831. 2012.
86. Isrctn. Post-deployment health screening of members of the United Kingdom Armed Forces: the POST study. http://wwwwhoint/trialsearch/Trial2aspx?TrialID=ISRCTN19965528. 2011.
87. Nct. Depression Attention for Women Now (The DAWN Study). https://clinicaltrialsgov/show/NCT01096316. 2010.
88. Actrn. The CREDO Research Project: can mood problems be prevented and treated using e-health interventions in patients being treated for cardiovascular disease? http://wwwwhoint/trialsearch/Trial2aspx?TrialID=ACTRN12610000085077. 2010.
89. Nct. Effectiveness of a Web-based Prevention Program for Postpartum Depression. https://clinicaltrialsgov/show/NCT00816725. 2009.
90. Nct. A Collaborative Care Program to Improve Depression Treatment in Cardiac Patients. https://clinicaltrialsgov/show/NCT00847132. 2009.
91. Ntr. The evaluation of a careprogram to increase the detection and treatment of depression in nursing homes. http://wwwwhoint/trialsearch/Trial2aspx?TrialID=NTR1477. 2008.
92. Isrctn. Screening and support for depression in people with diabetes: a randomised controlled study. http://wwwwhoint/trialsearch/Trial2aspx?TrialID=ISRCTN93089066. 2008.
93. Actrn. Take Heart in Primary Care Study. http://wwwwhoint/trialsearch/Trial2aspx?TrialID=ACTRN12606000371594. 2006.
94. Actrn. Randomised controlled trial of a New Zealand developed screening questionnaire for depression compared with a gold standard. http://wwwwhoint/trialsearch/Trial2aspx?TrialID=ACTRN12606000483550. 2006.
95. Nct. Outcome of Postnatal Depression Screening Using Edinburgh Postnatal Depression Scale. [https://clinicaltrialsgov/show/NCT00251342. 2005](https://clinicaltrialsgov/show/NCT00251342.%202005).
96. NCT02023957. A Pilot With Interactive Computer-assisted Screening for Mental Health in Primary Care. 2013

**Abstract (n=4)**

1. Draper T, Ormseth SR, Custodio M, Weisman MH, Irwin MR, Nicassio PM. Identifying subgroups of rheumatoid arthritis patients based on levels of pain, disability, and depression. 2012; 64:S673.
2. Wang S, Rowsell L, Wong K, Yee B, Eckert DJ, Somogyi A. Identifying Obstructive Sleep Apnea Patients Vulnerable to Opioid-Induced Respiratory Depression - A Randomized Double-Blind Placebo-Controlled Crossover Trial. 2016; 193(Meeting Abstracts):A4321.
3. Pibernik-Okanovic M, Sekerija M, Ajdukovic D, Hermanns N. The reach of screening for and treating of subsyndromal depression in type 2 diabetic patients. 2012; 55:S111‐2.
4. Goetz DM, Rand K, Roach CM, Cogswell A, Smith BA. Screening for depression in caregivers of childrens with cystic fibrosis. 2016; 51 Suppl 45:456, Abstract no: 685.

**Other (e.g., editorial, commentaries, letters, opinion pieces, case report) (n=12)**

1. Bond S. Us preventive services task force recommends routine depression screening in adults including pregnant and postpartum women. Journal of Midwifery and Women's Health 2016; 61(3):390-391.
2. [Grey Lit] Finley CT. Got Depression? I can answer that in two questions! Tools for Practice. January 2018. [www.acfp.ca](http://www.acfp.ca), accessed October 2018.
3. [Grey Lit] Patient-Reported Outcomes Measurement in Alberta: Potential of the EQ-5D. Symposium Proceedings. October 18-19, 2012. Solara Resort and Spa Canmore, AB.
4. [Grey Lit] What older adults, their families and friends need to know about… Depression. Centre for Addiction and Mental Health. Improving Our Response 2018.
5. [Grey Lit] Sheehan A, McGee H. Screening for depression in medical research: ethical challenges and recommendations. BMC Medical Ethics 2013; 14:4.
6. [Grey lit] Corby-Edwards on behalf of the American Psychological Association. RE: U.S. Preventive Services Task Force DRAFT Research Plan Primary Care Screening for Depression in Adult. April 23, 2014
7. [Grey Lit] Kato E, Beswick-Escanlar V. Screening for Depression in Adults. American Family Physician ([www.aafp.org/afp](http://www.aafp.org/afp)), accessed October 2018.
8. [Grey Lit] US Preventive Services Task Force. Screening for Depression Adults: Recommendation Statement. American Family Physician 2016; 94(4):340A-340D.
9. [Grey Lit] Anxiety and Depression Association of America. Depression. Accessed October 2018.
10. Correction: effect of depression screening after acute coronary syndromes on quality of life: the CODIACS-QoL randomized clinical trial (JAMA Internal MedicineJAMA Internal Medicine (2019) DOI: 10.1001/jamainternmed.2019.4518). JAMA internal medicine. 2019;179(12):1739‐.
11. Moise N, Davidson KW, Cheung YKK, et al. Rationale, design, and baseline data for a multicenter randomized clinical trial comparing depression screening strategies after acute coronary syndrome: The comparison of depression identification after acute Coronary Syndromes-Quality of Life and Cost Outcomes (CODIACS-QOL) trial. Contemporary Clinical Trials. 2019;84:105826.
12. Schuurhuizen C, Braamse AMJ, Konings I, Verheul HMW, Dekker J. Predictors for use of psychosocial services in patients with metastatic colorectal cancer receiving first line systemic treatment. BMC cancer. 2019;19(1):115.

**Study does not take place in a primary care or other non-mental health clinic settings (n=2)**

1. de Beurs DP, Ghoncheh R, Geraedts AS, Kerkhof AJFM. Psychological Distress Because of Asking about Suicidal Thoughts: A Randomized Controlled Trial among Students. Archives of Suicide Research 2016; 20(2):153-9.
2. Konradt B, Hirsch RD, Jonitz MF, Junglas K. Evaluation of a standardized humor group in a clinical setting: a feasibility study for older patients with depression. International Journal of Geriatric Psychiatry 2013; 28(8):850-857.

**Includes only those with existing depression/mental disorders or those seeking services due to symptoms of mental disorders (n=11)**

1. Bosanquet K, Adamson J, Atherton K, Bailey D, Baxter C, Beresford-Dent J, Birtwistle J, Chew-Graham C, Clare E, Delgadillo J, Ekers D, Foster D, Gabe R, Gascoyne S, Haley L, Hamilton J, Hargate R, Hewitt C, Holmes J, Keding A, Lewis H, McMillan D, Meer S, Mitchell N, Nutbrown S, Overend K, Parrott S, Pervin J, Richards DA, Spilsbury K, Torgerson D, Traviss-Turner G, Trepel D, Woodhouse R, Gilbody S. CollAborative care for Screen-Positive EldeRs with major depression (CASPER plus): a multicentred randomised controlled trial of clinical effectiveness and cost-effectiveness. Health Technology Assessment (Winchester, England) 2017; 21(67):1-252.
2. Kiosses DN, Rosenberg PB, McGovern A, Fonzetti P, Zaydens H, Alexopoulos GS. Depression and Suicidal Ideation During Two Psychosocial Treatments in Older Adults with Major Depression and Dementia. Journal of Alzheimer's disease 2015; 48(2):453-462.
3. Gustavson KA, Alexopoulos GS, Niu GC, McCulloch C, T Meade, Areán PA. Problem-Solving Therapy Reduces Suicidal Ideation In Depressed Older Adults with Executive Dysfunction. The American Journal of Geriatric Psychiatry 2016; 24(1):11-17.
4. Richards DA, Hill JJ, Gask L, Lovell K, Chew-Graham C, Bower P, Cape J, Pilling S, Araya R, Kessler D, Bland JM, Green C, Gilbody S, Lewis G, Manning C, Hughes-Morley A, Barkham M. Clinical effectiveness of collaborative care for depression in UK primary care (CADET): cluster randomised controlled trial. BMJ 2013; 347:f4913.
5. Zhao Y, Munro-Kramer ML, Shi S, Wang J, Zhao Q. Effects of antenatal depression screening and intervention among Chinese high-risk pregnant women with medically defined complications: A randomized controlled trial. Early Intervention in Psychiatry. 2019;13(5):1090-1098.
6. Sikander S, Ahmad I, Atif N, et al. Delivering the Thinking Healthy Programme for perinatal depression through volunteer peers: a cluster randomised controlled trial in Pakistan. The Lancet Psychiatry. 2019;6(2):128-139.
7. Schiff GD, Klinger E, Salazar A, et al. Screening for Adverse Drug Events: a Randomized Trial of Automated Calls Coupled with Phone-Based Pharmacist Counseling. Journal of General Internal Medicine. 2019;34(2):285-292.
8. Fuhr DC, Weobong B, Lazarus A, et al. Delivering the Thinking Healthy Programme for perinatal depression through peers: an individually randomised controlled trial in India. The Lancet Psychiatry. 2019;6(2):115-127.
9. Richards SH, Campbell JL, Dickens C, et al. Enhanced psychological care in cardiac rehabilitation services for patients with new-onset depression: the CADENCE feasibility study and pilot RCT. Health technology assessment (Winchester, England). 2018;22(30):1‐220.
10. Gidding LG, Spigt M, Winkens B, Dinant GJ, Herijgers O. Psyscan e-tool to support diagnosis and management of psychological problems in general practice: A randomised controlled trial. British Journal of General Practice. 2018;68(666):e18.
11. Actrn. A randomised, controlled evaluation of the ‘beyondblue depression training program for aged care staff’: impact on the delivery of health care services for older people with depression. http://wwwwhoint/trialsearch/Trial2aspx?TrialID=ACTRN12607000647437. 2007.

**Mixed population (e.g., those with/out existing depression), and does not provide disaggregated data (n=3)**

1. Cross K, Flores R, Butterfield J, Blackman M, Lee S. The effect of passive listening versus active observation of music and dance performances on memory recognition and mild to moderate depression in cognitively impaired older adults. Psychological Reports: Mental & Physical Health 2012; 111(2):413‐423.
2. Davison TE, Karantzas G, Mellor D, McCabe MP, Mrkic D. Staff-focused interventions to increase referrals for depression in aged care facilities: a cluster randomized controlled trial. Aging & Mental Health 2013; 17(4):449-55.
3. Edward KL, Giandinoto JA, Stephenson J, Mills C, McFarland J, Castle DJ. Self- screening using the Edinburgh post natal depression scale for mothers and fathers to initiate early help seeking behaviours. Archives of Psychiatric Nursing. 2019;33(4):421-427.

**Intervention group does not receive an intervention that uses a single question, small sets of questions, or a screening questionnaire (validated or non-validated) with a pre-defined cut-off score to identify patients who may have depression, but who have not reported their symptoms to healthcare providers or who have otherwise not been identified as possibly depressed by healthcare providers (n=1)**

1. Poleshuck EW, M.//Crean, H. F.//Juskiewicz, I.//Bell, E.//Harrington, A.//Cerulli, C. A Comparative Effectiveness Trial of Two Patient-Centered Interventions for Women with Unmet Social Needs: Personalized Support for Progress and Enhanced Screening and Referral. Journal of Women's Health. 2020;29(2):242-252.

**Does not determine patient eligibility and randomized patients prior to administering the screening test (n=15)**

1. Actrn. A randomised controlled trial of a specialised multidisciplinary consultation team to improve the outcomes of patients with recent onset spinal cord injury in acute hospital. http://wwwwhoint/trialsearch/Trial2aspx?TrialID=ACTRN12610000164099. 2010.
2. Albert SM, King J, Anderson S, et al. Depression Agency-Based Collaborative: Effect of Problem-Solving Therapy on Risk of Common Mental Disorders in Older Adults With Home Care Needs. American Journal of Geriatric Psychiatry. 2019;27(6):619-624.
3. Albert SM, King J, Dew MA, Begley A, Anderson S, Karp J, Gildengers A, Butters M, Reynolds CF 3rd. Design and Recruitment for a Randomized Controlled Trial of Problem-Solving Therapy to Prevent Depression among Older Adults with Need for Supportive Services. The American Journal of Geriatric Psychiatry 2016; 24(1):94-102.
4. Celano CM, Suarez L, Mastromauro C, Januzzi JL, Huffman JC. Feasibility and utility of screening for depression and anxiety disorders in patients with cardiovascular disease. Circulation. Cardiovascular Quality and Outcomes 2013; 6(4):498-504.
5. Cho SK, Kim JJ, Park YG, Ko HS, Park IY, Shin JC. The simplified Edinburgh Postnatal Depression Scale (EPDS) for antenatal depression: is it a valid measure for pre-screening? International Journal of Medical Sciences 2012; 9(1):40-6.
6. Choi I, Milne DN, Deady M, Calvo RA, Harvey SB, Glozier N. Impact of Mental Health Screening on Promoting Immediate Online Help-Seeking: Randomized Trial Comparing Normative Versus Humor-Driven Feedback. JMIR mental health 2018; 5(2):e26.
7. Corones-Watkins KM, Theobald KA, White KM. Outcomes of a randomised pilot trial of a nurse-led clinic for patients after percutaneous coronary intervention. Australian Critical Care. 2019;32(4):285-292.
8. Isrctn. A multicomponent frailty intervention in community-dwelling elderly persons. http://wwwwhoint/trialsearch/Trial2aspx?TrialID=ISRCTN17143761. 2019.
9. Jordans MJD, Luitel NP, Lund C, Kohrt BA. Evaluation of Proactive Community Case Detection to Increase Help Seeking for Mental Health Care: A Pragmatic Randomized Controlled Trial. Psychiatric services (Washington, DC). 2020:appips201900377.
10. Karp JF, Zhang J, Wahed AS, et al. Improving Patient Reported Outcomes and Preventing Depression and Anxiety in Older Adults With Knee Osteoarthritis: Results of a Sequenced Multiple Assignment Randomized Trial (SMART) Study. American Journal of Geriatric Psychiatry. 2019;27(10):1035-1045.
11. Kastello JC, Jacobsen KH, Gaffney KF, Kodadek MP, Bullock LC, Sharps PW. Self-Rated Mental Health: Screening for Depression and Posttraumatic Stress Disorder Among Women Exposed to Perinatal Intimate Partner Violence. Journal of Psychosocial Nursing and Mental Health Services 2015; 53(11):32-8.
12. Kauye F, Jenkins R, Rahman A. Training primary health care workers in mental health and its impact on diagnoses of common mental disorders in primary care of a developing country, Malawi: a cluster-randomized controlled trial. Psychological medicine 2014; 44(3):657-66.
13. Lysack C, Leach C, Russo T, Paulson D, Lichtenberg Peter A. DVD training for depression identification and treatment in older adults: a two-croup, randomized, wait-list control study. American Journal of Occupational Therapy 2013; 67(5):584‐593.
14. Montag AC, Brodine SK, Alcaraz JE, Clapp JD, Allison MA, Calac DJ, Hull AD, Gorman JR, Lyons Jones K, Chambers CD. Effect of Depression on Risky Drinking and Response to a Screening, Brief Intervention, and Referral to Treatment Intervention. American Journal of Public Health 2015; 105(8):1572-6.
15. Picardi A, Lega I, Tarsitani L, Caredda M, Matteucci G, Zerella MP, Miglio R, Gigantesco A, Cerbo M, Gaddini A, Spandonaro F, Biondi M, SET-DEP Group. A randomised controlled trial of the effectiveness of a program for early detection and treatment of depression in primary care. Journal of affective disorders 2016; 198:96-101.

**All participants in the control group receive screening and/or results are provided to patients or healthcare providers (n=8)**

1. Haga SM, Drozd F, Lisoy C, Wentzel-Larsen T, Slinning K. Mamma Mia-A randomized controlled trial of an internet-based intervention for perinatal depression. Psychological Medicine. 2019;49(11):1850-1858.
2. Hoodin F, Zhao L, Carey J, Levine JE, Kitko C. Impact of psychological screening on routine outpatient care of hematopoietic cell transplantation survivors. Biology of blood and marrow transplantation 2013; 19(10):1493-7.
3. Jin H, Wu S. Text Messaging as a Screening Tool for Depression and Related Conditions in Underserved, Predominantly Minority Safety Net Primary Care Patients: Validity Study. Journal of Medical Internet Research. 2020;22(3):e17282.
4. Lebowitz MS, Ahn W-K. Testing positive for a genetic predisposition to depression magnifies retrospective memory for depressive symptoms. Journal of consulting and clinical psychology 2017; 85(11):1052-1063.
5. Löwe B, Blankenberg S, Wegscheider K, König HH, Walter D, Murray AM, Gierk B, Kohlmann S. Depression screening with patient-targeted feedback in cardiology: dEPSCREEN-INFO randomised clinical trial. The British Journal of Psychiatry 2017; 210(2):132‐139.
6. Rauwerda NL, Tovote KA, Peeters A, et al. WHO-5 and BDI-II are acceptable screening instruments for depression in people with diabetes. Diabetic medicine. 2018;35(12):1678‐1685.
7. Rona RJ, Burdett H, Khondoker M, Chesnokov M, Green K, Pernet D, Jones N, Greenberg N, Wessely S, Fear NT. Post-deployment screening for mental disorders and tailored advice about help-seeking in the UK military: a cluster randomised controlled trial. Lancet (London, England) 2017; 389(10077):1410-1423.
8. van Dijk DJA, Crone RM, van Empelen P, Assendelft JW, Middelkoop JB. Favourable outcomes of a preventive screening and counselling programme for older people in underprivileged areas in the Netherlands: the PRIMUS project. Preventive Medicine Reports 2017; 6:258‐264.

**Control group received depression screening to test for acceptability/feasibility (n=5)**

1. Kingston D, Austin M-P, Veldhuyzen van Zanten S, Harvalik P, Giallo R, McDonald SD, MacQueen G, Vermeyden L, Lasiuk G, Sword W, Biringer A. Pregnant Women's Views on the Feasibility and Acceptability of Web-Based Mental Health E-Screening Versus Paper-Based Screening: A Randomized Controlled Trial. Journal of Medical Internet Research 2017; 19(4):e88.
2. Kingston D, Biringer A, Veldhuyzen van Zanten S, Giallo R, McDonald S, MacQueen G, Vermeyden L, Austin M-P. Pregnant Women's Perceptions of the Risks and Benefits of Disclosure During Web-Based Mental Health E-Screening Versus Paper-Based Screening: Randomized Controlled Trial. JMIR Mental Health 2017; 4(4):e42.
3. Marcano-Belisario JS, Gupta AK, O'Donoghue J, Ramchandani P, Morrison C, Car J. Implementation of depression screening in antenatal clinics through tablet computers: results of a feasibility study. BMC Medical Informatics and Decision Making 2017; 17(1):59.
4. Saliba D, DiFilippo S, Edelen MO, Kroenke K, Buchanan J, Streim J. Testing the PHQ-9 Interview and Observational Versions (PHQ-9 OV) for MDS 3.0. Journal of the American Medical Directors Association 2012; 13(7):618-625.
5. van Dijk DJA, Crone MR, van Empelen P, Assendelft WJ, Middelkoop BJ. Favourable outcomes of a preventive screening and counselling programme for older people in underprivileged areas in the Netherlands: The PRIMUS project. Preventive medicine reports 2017; 6():258-264.

**Study does not provide similar depression management and treatment resources to patients who were identified as depressed via screening in the screening arm of the trial and patients in either the screening or non-screening arms of the trial who were identified as depressed via other methods (n=6)**

1. Bobo WV, Wollan P, Lewis G, Bertram S, Kurland MJ, Vore K, Yawn BP. Depressive symptoms and access to mental health care in women screened for postpartum depression who lose health insurance coverage after delivery: findings from the Translating Research into Practice for Postpartum Depression (TRIPPD) effectiveness study. Mayo Clinic proceedings 2014; 89(9):1220-8.
2. Browne AL, Appleton S, Fong K, Wood F, Coll F, de Munck S, Newnham E, Schug SA. A pilot randomized controlled trial of an early multidisciplinary model to prevent disability following traumatic injury. Disability and Rehabilitation 2013; 35(14):1149-63.
3. Noben C, Smit F, Nieuwenhuijsen K, Ketelaar S, Gartner F, Boon B, Sluiter J, Evers S. Comparative cost-effectiveness of two interventions to promote work functioning by targeting mental health complaints among nurses: pragmatic cluster randomised trial. International journal of nursing studies 2014; 51(10):1321-31.
4. Silverstone PH, Rittenbach K, Suen VYM, Moretzsohn A, Cribben I, Bercov M, Allen A, Pryce C, Hamza DM, Trew M. Depression Outcomes in Adults Attending Family Practice Were Not Improved by Screening, Stepped-Care, or Online CBT during a 12-Week Study when Compared to Controls in a Randomized Trial. Frontiers in Psychiatry 2017; 8:32.
5. Singer S, Danker H, Roick J, Einenkel J, Briest S, Spieker H, Dietz A, Hoffmann I, Papsdorf K, Meixensberger J, Mossner J, Schiefke F, Dietel A, Wirtz H, Niederwieser D, Berg T, Kersting A. Effects of stepped psychooncological care on referral to psychosocial services and emotional well-being in cancer patients: A cluster-randomized phase III trial. Psycho-oncology 2017; 26(10):1675-1683.
6. Yawn BP, Dietrich AJ, Wollan P, Bertram S, Graham D, Huff J, Kurland M, Madison S, Pace WD, TRIPPD practices. TRIPPD: a practice-based network effectiveness study of postpartum depression screening and management. Annals of Family Medicine 2012; 10(4):320-9.

**The doctors in the control group are given a generic prompt which recommends that patients be screened for (n=1)**

1. Carroll AE, Biondich P, Anand V, Dugan TM, Downs SM. A randomized controlled trial of screening for maternal depression with a clinical decision support system. Journal of the American Medical Informatics Association 2013; 20(2):311-316.

**Results provided are only those identified as having MDD, does not present information on the entire population that were randomized, searches medical records for depression with no timeline on how far back it was searched (and screening at baseline to follow-up was six months) (n=1)**

1. Romera I, Montejo AL, Aragones E, Arbesu JA, Iglesias-Garcia C, Lopez S, Lozano JA, Pamulapati S, Yruretagoyena B, Gilaberte I. Systematic depression screening in high-risk patients attending primary care: a pragmatic cluster-randomized trial. *BMC Psychiatry* 2013; 13:83.

**Not specific to depression screening, but overall psychosocial screening (n=3)**

1. Braeken APBM, Kempen GIJM, Eekers DBP, Houben RMA, van Gils FCJM, Ambergen T, Lechner L. Psychosocial screening effects on health-related outcomes in patients receiving radiotherapy. A cluster randomised controlled trial. Psycho-oncology 2013; 22(12):2736-46.
2. Barrera M, Alexander S, Atenafu EG, et al. Psychosocial screening and mental health in pediatric cancer: A randomized controlled trial. Health Psychology. 2020;39(5):381-390.
3. Schuurhuizen CSEW, Braamse AMJ, Beekman ATF, et al. Screening and stepped care targeting psychological distress in patients with metastatic colorectal cancer: The TES cluster randomized trial. JNCCN Journal of the National Comprehensive Cancer Network. 2019;17(8):911-920.

## Additional file 8. List of ongoing trials in the general adult population

| **Trial Identifier** | **Study Title** | **Study Start Date** | **Estimated Study Completion Date** |
| --- | --- | --- | --- |
| NCT03215199 | Northern Alberta Psychosocial Telecare (NAPT) Screening for HNC Patients | September 2017 | October 2018  (no publication available) |
| ISRCTN12154418 | Improving the care of people with long term conditions (ENHANCE) | September 2014 | December 2016  (no publication available) |
| ACTRN 12616001217493 | Integrated e-Screening for Postnatal Depression and Anxiety- feasibility and effectiveness | Not reported | Not reported  (no publication available) |
| NCT00433238 | Screening for Mental Health Concerns for at-Risk Community Living Chinese Seniors | March 2007 | “Finished” based on author follow-up  (no publication available) |
| NCT: National Clinical Trial; ISRCTN: International Standard Randomised Controlled Trial Number | | | |

## Additional file 9. Pregnancy and postpartum excluded studies bibliography with reason

**Full-text not available (n=1)**

1. Felice W, Agius A, Sultana R, Felice EM, Calleja-Agius J. The effectiveness of psychosocial assessment in the detection and management of postpartum depression: a systematic review. *Minerva Ginecologica* 2018; 70(3):323.

**Not English or French (n=2)**

1. A Kersting. [Peripartum depression and grief after pregnancy loss: special problem areas in obstetrics]. *Der Nervenarzt* 2012//; 83(11):1434 [German]
2. [Grey lit] Depresjonsscreening av gravide og barselkvinner. Rapport fra Kunnskapssenteret nr 1-2013. Systematisk oversikt. [Norwegian]

**Systematic reviews/Guidelines (n=8)**

1. Fleming N, O'Driscoll T, Becker G, Spitzer RF, CANPAGO COMMITTEE. Adolescent Pregnancy Guidelines. Journal of Obstetrics and Gynaecology Canada 2015; 37(8):740-756.
2. Thombs BD, Arthurs E, Coronado-Montoya S, Roseman M, Delisle VC, Leavens A, Levis B, Azoulay L, Smith C, Ciofani L, Coyne JC, Feeley N, Gilbody S, Schinazi J, Stewart DE, Zelkowitz P. Depression screening and patient outcomes in pregnancy or postpartum: a systematic review. Journal of Psychosomatic Research 2014; 76(6):433-446.
3. Hewitt CE, Gilbody SM. Is it clinically and cost effective to screen for postnatal depression: a systematic review of controlled clinical trials and economic evidence. BJOG: An International Journal of Obstetrics and Gynaecology 2009; 116(8):1019-1027.
4. Austin M-P, Priest SR, Sullivan EA. Antenatal psychosocial assessment for reducing perinatal mental health morbidity. Cochrane Database of Systematic Reviews 2008;(4):CD005124.
5. Reinsperger I, Winkler R, Piso B. Identifying sociomedical risk factors during pregnancy: recommendations from international evidence-based guidelines. J Public Health 2015; 23(1):1‐13.
6. Reilly NK, D.//Loxton, D.//Talcevska, K.//Austin, M. P. A narrative review of studies addressing the clinical effectiveness of perinatal depression screening programs. Women Birth. 2020;33(1):51-59.
7. Maxwell CG, L.//Cassir, G.//Nowik, C.//McLeod, N. L.//Jacob, C. E.//Walker, M. Guideline No. 392-Pregnancy and Maternal Obesity Part 2: Team Planning for Delivery and Postpartum Care. Journal of Obstetrics and Gynaecology Canada. 2019;41(11):1660-1675.
8. O'Connor ES, C. A.//Henninger, M. L.//Coppola, E.//Gaynes, B. N. Interventions to Prevent Perinatal Depression: Evidence Report and Systematic Review for the US Preventive Services Task Force. Obstetrical and Gynecological Survey. 2019;74(6):317-318.

**Observational studies (e.g., cohorts, case-controls, cross-sectional, case series) (n=25)**

1. McKean M, Caughey AB, Yuracko McKean MA, Cabana MD, Flaherman VJ. Postpartum Depression: When Should Health Care Providers Identify Those at Risk? Clinical Pediatrics 2018; 57(6):689-693.
2. Yawn BP, Bertram S, Kurland M, Wollan PC. Repeated depression screening during the first postpartum year. Annals of Family Medicine 2015; 13(3):228-234.
3. Venkatesh KK, Zlotnick C, Triche EW, Ware C, Phipps MG. Accuracy of brief screening tools for identifying postpartum depression among adolescent mothers. Pediatrics 2014; 133(1):e45-e53.
4. Birmingham MC, Chou KJ, Crain EF. Screening for postpartum depression in a pediatric emergency department. Pediatric Emergency Care 2011; 27(9):795-800.
5. Svanberg PO, Mennet L, Spieker S. Promoting a secure attachment: A primary prevention practice model. Clinical Child Psychology and Psychiatry 2010; 15(3):363-378.
6. Petrou S, Morrell J, Spiby H. Assessing the empirical validity of alternative multi-attribute utility measures in the maternity context. Health and quality of life outcomes 2009; 7:40.
7. Harrington AR, Greene-Harrington CG. Healthy Start screens for depression among urban pregnant, postpartum and interconceptional women. Journal of the National Medical Association 2007; 99(3):226-231.
8. Jardri R, Pelta J, Maron M, Thomas P, Delion P, Codaccioni X, Goudemand M. Predictive validation study of the Edinburgh Postnatal Depression Scale in the first week after delivery and risk analysis for postnatal depression. Journal of affective disorders 2006; 93(1-3):169-176.
9. Olson AL, Dietrich AJ, Prazar G, Hurley J, Tuddenham A, Hedberg V, Naspinsky DA. Two approaches to maternal depression screening during well child visits. Journal of developmental and behavioral pediatrics 2005; 26(3):169-176.
10. Garcia-Esteve L, Ascaso C, Ojuel J, Navarro P. Validation of the Edinburgh Postnatal Depression Scale (EPDS) in Spanish mothers. Journal of Affective Disorders 2003; 75(1):71-76.
11. Davis K, Pearlstein T, Stuart S, O'Hara M, Zlotnick C. Analysis of brief screening tools for the detection of postpartum depression: Comparisons of the PRAMS 6-item instrument, PHQ-9, and structured interviews Archives of Women's Mental Health 2013; 16(4):271-277.
12. Milgrom J, Ericksen J, Negri L, Gemmill AW. Screening for postnatal depression in routine primary care: Properties of the Edinburgh Postnatal Depression Scale in an Australian sample Australian and New Zealand Journal of Psychiatry 2005; 39(9):833-839.
13. Leverton TJ, Elliott SA. Is the EPDS a magic wand? 1. A comparison of the Edinburgh Postnatal Depression Scale and health visitor report as predictors of diagnosis on the present state examination. Journal of Reproductive and Infant Psychology 2000; 18(4):279-296.
14. Wisner KL, Sit DK, McShea MC, Rizzo DM, Zoretich RA, Hughes CL. Onset timing, thoughts of self-harm, and diagnoses in postpartum women with screen-positive depression findings. JAMA Psychiatry 2013; 70(5):490‐498.
15. Thomas DV, Looney SW. Effectiveness of a comprehensive psychoeducational intervention with pregnant and parenting adolescents: a pilot study. Journal of Child and Adolescent Psychiatric Nursing 2004; 17(2):66-77.
16. Barnet B, Liu J, Devoe M. Double jeopardy: depressive symptoms and rapid subsequent pregnancy in adolescent mothers. Arch Pediatr Adolesc Med 2008;162(3):246-252.
17. Logsdon MC, Foltz MP, Stein B, Usui W, Josephson A. Adapting and testing telephone-based depression care management intervention for adolescent mothers. Arch Womens Ment Health 2010; 13(4):307-317.
18. Evins GG, Theofrastous JP, Galvin SL. Postpartum depression: a comparison of screening and routine clinical evaluation. Am J Obstet Gynecol 2000; 182(5):1080-1082.
19. Glavin K, Smith L, Sørum R, Ellefsen B. Redesigned community postpartum care to prevent and treat postpartum depression in women--a one-year follow-up study. Journal of Clinical Nursing 2010; 19(21-22):3051-3062.
20. Wickberg B, Tjus T, Hwang P. Using the EPDS in routine antenatal care in Sweden: a naturalistic study. Journal of Reproductive and Infant Psychology 2005; 23(1):33-41.
21. Dadi AFM, E. R.//Woodman, R.//Bisetegn, T. A.//Mwanri, L. Antenatal depression and its potential causal mechanisms among pregnant mothers in Gondar town: application of structural equation model. BMC Pregnancy Childbirth. 2020;20(1):168.
22. Kubota CI, T.//Nakamura, Y.//Shiino, T.//Ando, M.//Aleksic, B.//Yamauchi, A.//Morikawa, M.//Okada, T.//Ohara, M.//Sato, M.//Murase, S.//Goto, S.//Kanai, A.//Ozaki, N. Stable factor structure of the Edinburgh Postnatal Depression Scale during the whole peripartum period: Results from a Japanese prospective cohort study. Scientific Reports. 2018;8(1):17659.
23. Gutierrez-Zotes AG-P, D.//Labad, J.//Martin-Santos, R.//Garcia-Esteve, L.//Gelabert, E.//Jover, M.//Guillamat, R.//Mayoral, F.//Gornemann, I.//Canellas, F.//Gratacos, M.//Guitart, M.//Roca, M.//Costas, J.//Ivorra, J. L.//Navines, R.//de Diego, Y.//Vilella, E.//Sanjuan, J. Factor Structure of the Spanish Version of the Edinburgh Postnatal Depression Scale. Actas Esp Psiquiatr. 2018;46(5):174-182.
24. Hompoth EAP, Z.//Fureszne Balogh, V.//Toreki, A. Associations Between Depression Symptoms, Psychological Intervention and Perinatal Complications. Journal of Clinical Psychology in Medical Settings. 2020;27(1):199-205.
25. Yamauchi AO, T.//Ando, M.//Morikawa, M.//Nakamura, Y.//Kubota, C.//Ohara, M.//Murase, S.//Goto, S.//Kanai, A.//Ozaki, N. Validation and factor analysis of the Japanese version of the highs scale in perinatal women. Frontiers in Psychiatry. 2018;9 (no pagination)(269).

**Narrative/literature reviews (non-systematic) (n=15)**

1. O'Hara MW, McCabe JE. Postpartum depression: current status and future directions. *Annual Review of Clinical Psychology* 2013; 9:379-407.
2. Yozwiak JA. Postpartum depression and adolescent mothers: a review of assessment and treatment approaches. *Journal of Pediatric and Adolescent Gynecology* 2010; 23(3):172-178.
3. Holden JM. Postnatal depression: its nature, effects, and identification using the Edinburgh Postnatal Depression scale. *Birth (Berkeley, Calif.)* 1991; 18(4):211-221.
4. Ukatu N, Clare CA, Brulja M. Postpartum Depression Screening Tools: A Review. *Psychosomatics* 2018; 59(3):211-219.
5. Langan RC, Goodbred AJ. Identification and management of peripartum depression. *American Family Physician* 2016; 93(10):852-858.
6. Payne JL. Recent Advances and Controversies in Peripartum Depression. *Current Obstetrics and Gynecology Reports* 2016; 5(3):250-256.
7. Yilmaz EA, Gulumser C. The risk factors, consequences, treatment, and importance of gestational depression. *J Turk Soc Obstet Gynecol* 2015; 12(2):102-113.
8. Pope CJ, Sharma V, Mazmanian D. Recognition, diagnosis and treatment of postpartum bipolar depression. *Expert Review of Neurotherapeutics* 2014; 14(1):19-28.
9. Drozdowicz E ,Rodzoch R, Matsumoto H, Skalski M, Wielgos M, Radziwon-Zaleska M. Postpartum depression - a review of current knowledge *Medical Science Technology* 2013; 54(1):125-129.
10. Clare CA, Yeh J. Postpartum depression in special populations: A review. *Obstetrical and Gynecological Survey* 2012; 67(5):313-323.
11. Chabrol H, Callahan S. Prevention and treatment of postnatal depression. *Expert Review of Neurotherapeutics* 2007; 7(5):557-576.
12. Haller E. Depression during and after pregnancy: What does the primary care physician need to know? *Advanced Studies in Medicine* 2005; 5(1):21-26.
13. Gale S, Harlow BL. Postpartum mood disorders: A review of clinical and epidemiological factors. *Journal of Psychosomatic Obstetrics and Gynecology* 2003; 24(4):257-266.
14. [Grey lit] Glavin K, Leahy-Warren P. Postnatal Depression Is a Public Health Nursing Issue: Perspectives from Norway and Ireland. *Nursing Research and Practice* 2013; Article ID 813409, 7 pages.
15. McClanahan KK. Depression in pregnant adolescents: considerations for treatment. *J Pediatr Adolesc Gynecol* 2009; 22(1):59-64.

**Protocol (n=32)**

1. Marcano Belisario JS, Doherty K, O'Donoghue J, Ramchandani P, Majeed A, Doherty G, Morrison C, Car J. A bespoke mobile application for the longitudinal assessment of depression and mood during pregnancy: protocol of a feasibility study. BMJ Open 2017; 7(5):e014469.
2. Marcano-Belisario JS, Gupta AK, O'Donoghue J, Morrison C, Car J. Tablet computers for implementing NICE antenatal mental health guidelines: protocol of a feasibility study. BMJ Open 2016; 6(1):e009930.
3. Kingston D, Austin M-P, Hegadoren K, McDonald S, Lasiuk G, McDonald S, Heaman M, Biringer A, Sword W, Giallo R, Patel T, Lane-Smith M, Veldhuyzen van Zanten S. Study protocol for a randomized, controlled, superiority trial comparing the clinical and cost- effectiveness of integrated online mental health assessment-referral-care in pregnancy to usual prenatal care on prenatal and postnatal mental health and infant health and development: the Integrated Maternal Psychosocial Assessment to Care Trial (IMPACT). Trials 2014; 15:72.
4. Kingston D, McDonald S, Biringer A, Austin M-P, Hegadoren K, McDonald S, Giallo R, Ohinmaa A, Lasiuk G, MacQueen G, Sword W, Lane-Smith M, Veldhuyzen van Zanten S. Comparing the feasibility, acceptability, clinical-, and cost-effectiveness of mental health e-screening to paper-based screening on the detection of depression, anxiety, and psychosocial risk in pregnant women: a study protocol of a randomized, parallel-group, superiority trial. Trials 2014: 15:3.
5. [Grey Lit] ISRCTN10697380. Multi-centre randomized controlled trial for postnatal depression in British South Asian women – ROSHNI-2.
6. [Grey Lit] ISRCTN42298046. The effectiveness of screening for postpartum depression in child health care.
7. [Grey Lit] NCT02516982. mHealth for Antenatal Mental Health (AMHS).
8. [Grey Lit] NCT03615794. A Study of Pregnant and Postpartum Women With and Without Mood Disorders.
9. [Grey Lit] NCT00548743. Translating Research Into Practice for Postpartum Depression (TRIPPD).
10. [Grey Lit] NCT00251342. Outcome of Postnatal Depression Screening Using Edinburgh Postnatal Depression Scale.
11. [Grey Lit] NCT01899534. Mental Health E-screening in Pregnant and Postpartum Women.
12. [Grey Lit] ChiCTR9IOR-15006433. Study of the Effects of Prenatal Screening Psychological Intervention on the High-risk Pregnant Women’s Postpartum Depression.
13. [Grey Lit] NCT01773629. Care Manager for Perinatal Depression (CMPD).
14. [Grey Lit] NCT03448289. Use of a Reproductive Life Planning Tool at the Pediatric Well-Baby Visit with Postpartum Women.
15. [Grey Lit] NCT00056901. Screening Evaluation for Women With Postpartum Depression.
16. [Grey Lit] NCT01238614. Universal Screening for Maternal Depression with the CHICA System.
17. [Grey Lit] NCT00282776. Identification and Therapy of Postpartum Depression.
18. [Grey Lit] ACTRN12616001217493. Integrated e-Screening for Postnatal Depression and Anxiety.
19. Aarestrup AKSV, M.//Petersen, J.//Rohder, K.//Schiotz, M. An early intervention to promote maternal sensitivity in the perinatal period for women with psychosocial vulnerabilities: study protocol of a randomized controlled trial. BMC psychology. 2020;8(1):41.
20. Hamel CL, E.//Morissette, K.//Beck, A.//Stevens, A.//Skidmore, B.//Colquhoun, H.//LeBlanc, J.//Moore, A.//Riva, J. J.//Thombs, B. D.//Colman, I.//Grigoriadis, S.//Nicholls, S. G.//Potter, B. K.//Ritchie, K.//Robert, J.//Vasa, P.//Lauria-Horner, B.//Patten, S.//Vigod, S. N.//Hutton, B.//Shea, B. J.//Shanmugasegaram, S.//Little, J.//Moher, D. Screening for depression in women during pregnancy or the first year postpartum and in the general adult population: a protocol for two systematic reviews to update a guideline of the Canadian Task Force on Preventive Health Care. Systematic Reviews. 2019;8(1):27.
21. Carlin EB, S. J.//Cadet-James, Y.//Campbell, S.//Williams, M.//Engelke, C.//Taverner, D.//Marriott, R.//Edmonds, K.//Atkinson, D.//Marley, J. V. Study protocol: a clinical trial for improving mental health screening for Aboriginal and Torres Strait Islander pregnant women and mothers of young children using the Kimberley Mum's Mood Scale. BMC Public Health. 2019;19(1):1521.
22. Moore Simas TAB, L.//Sankaran, P.//Masters, G. A.//Person, S.//Weinreb, L.//Ko, J. Y.//Robbins, C. L.//Allison, J.//Byatt, N. The PRogram In Support of Moms (PRISM): study protocol for a cluster randomized controlled trial of two active interventions addressing perinatal depression in obstetric settings. BMC Pregnancy Childbirth. 2019;19(1):256.
23. Steardo L, Jr.//Caivano, V.//Sampogna, G.//Di Cerbo, A.//Fico, G.//Zinno, F.//Del Vecchio, V.//Giallonardo, V.//Torella, M.//Luciano, M.//Fiorillo, A. Psychoeducational Intervention for Perinatal Depression: Study Protocol of a Randomized Controlled Trial. Front Psychiatr. 2019;10:55.
24. Actrn. The post-operative review as an opportunity to intervene for postnatal depression in mothers undergoing Caesarean section. http://wwwwhoint/trialsearch/Trial2aspx?TrialID=ACTRN12618001577202. 2018.
25. Nct. Minimum Intervention to Maintain a Postpartum Depression Prevention Program in Clinics Serving Low-income Women. https://clinicaltrialsgov/show/NCT03267563. 2017.
26. Nct. MAMA NO STRESS Project. The Effects of the "HAPPY MAMA" Intervention. https://clinicaltrialsgov/show/NCT04093791. 2019.
27. Ntr. Healthy Pregnancy 4 All -2: maternity care. http://wwwwhoint/trialsearch/Trial2aspx?TrialID=NTR6311. 2017.
28. Actrn. Perinatal Identification, Referral and Integrated Management for Improving Depression: the PIRIMID Study. http://wwwwhoint/trialsearch/Trial2aspx?TrialID=ACTRN12619001433190. 2019.
29. Nct. Integrated Maternal Psychosocial Assessment to Care Trial (IMPACT) Full Trial. https://clinicaltrialsgov/show/NCT01901796. 2013.
30. Nct. PRogram In Support of Moms: an Innovative Stepped-Care Approach for Obstetrics and Gynecology Clinics. https://clinicaltrialsgov/show/NCT02760004. 2016.
31. Pactr. Developing a screening tool for postnatal Depression in Zimbabwe. http://wwwwhoint/trialsearch/Trial2aspx?TrialID=PACTR201303000485383. 2013.
32. Nct. Depression Attention for Women Now (The DAWN Study). https://clinicaltrialsgov/show/NCT01096316. 2010.

**Abstract (n=2)**

1. Murray KE, Nyp SS. Postpartum depression. *Journal of Developmental and Behavioral Pediatrics* 2011; 32(2):175.
2. Anonymous. Abstracts from Research Forums Presented at the American College of Nurse-Midwives' 62nd Annual Meeting. *Journal of Midwifery and Women's Health Conference: 62nd Annual Meeting and Exhibition of the American College of Nurse Midwives, ACNM.* 2017;62(5).

**Other (e.g., editorial, commentaries, letters, opinion pieces, case reports) (n=13)**

1. Terrazas C, Segre LS, Wolfe C. Moving beyond depression screening: integrating perinatal depression treatment into OB/GYN practices. *Primary health care research & development* 2018:1-9.
2. Hewitt C, Gilbody S, Brealey S, Paulden M, Palmer S, Mann R, Green J, Morrell J, Barkham M, Light K, Richards D. Methods to identify postnatal depression in primary care: an integrated evidence synthesis and value of information analysis. *Health technology assessment* 2009; 13(36).
3. Reynolds CF, Patel V. Screening for depression: the global mental health context. *World Psychiatry* 2017; 16(3):316-317.
4. Bond S. Us preventive services task force recommends routine depression screening in adults including pregnant and postpartum women. *Journal of Midwifery and Women's Health* 2016; 61(3):390-391.
5. Slattengren AH, Prasad S, Altaf Kaiseruddin M. Should you screen for postpartum depression? *Journal of Family Practice* 2013; 62(11):E1-E3.
6. Kent A. What's new in the other journals? *BJOG: An International Journal of Obstetrics and Gynaecology* 2012; 119(6):770-771.
7. Austin MP. Antenatal screening and early intervention for "perinatal" distress, depression and anxiety: Where to from here? *Archives of Women's Mental Health* 2004; 7(1):1-6.
8. Thombs BD, Stewart DE. Depression screening in pregnancy and postpartum: How close are we? *Journal of Psychosomatic Research* 2014; 77(3):244-245.
9. Sidebottom AC, Harrison PA, Godecker A, Kim H. Response to letter from Coronado-Montoya et al. *Archives of Women's Mental Health* 2013; 16(3):255-256.
10. Lok HI. Psychological morbidity after miscarriage. *Dissertation Abstracts International: Section B: The Sciences and Engineering* 2007; 68(3-B):1567.
11. Matthey S. Assessing for psychosocial morbidity in pregnant women. *Canadian Medical Association Journal* 2005; 173(3):267-269.
12. [Grey lit] Corby-Edwards on behalf of the American Psychological Association. RE: U.S. Preventive Services Task Force DRAFT Research Plan Primary Care Screening for Depression in Adult. April 23, 2014
13. [Grey lit] APA Public Interest Government Relations Office. Postpartum Depression.

**Does not include pregnant/postpartum women (n=1)**

1. Montag AC, Brodine SK, Alcaraz JE, Clapp JD, Allison MA, Calac DJ, Hull AD, Gorman JR, Jones KL, Chambers CD. Effect of Depression on Risky Drinking and Response to a Screening, Brief Intervention, and Referral to Treatment Intervention. *American Journal of Public Health* 2015; 105(8):1572-1576.

**Includes ONLY those with existing depression/mental disorders or those seeking services due to symptoms of mental disorders (n=5)**

1. Peindl KS, Wisner KL, Hanusa BH. Identifying depression in the first postpartum year: guidelines for office-based screening and referral. *Journal of Affective Disorders* 2004; 80(1):37-44.
2. [Grey Lit] Brugha TS, Wheatley S, Taub NA, Culverwell A, Friedman T, Kirwan P, Jones DR, Shapiro DA. Pragmatic randomized trial of antenatal intervention to prevent post-natal depression by reducing psychosocial risk factors. *Psychological Medicine* 2000; 30:1273-1281. **FOLLOW-UP FROM PROTOCOL
3. [Grey Lit] Segre LS, Brock RL, O’Hara MW. Depression Treatment for Impoverished Mothers by Point-of-Care Providers: A Randomized Controlled Trial. *J Consult Clin Psychol* 2015; 83(2):314-324. **FOLLOW-UP FROM PROTOCOL
4. Byatt NMS, T. A.//Biebel, K.//Sankaran, P.//Pbert, L.//Weinreb, L.//Ziedonis, D.//Allison, J. PRogram In Support of Moms (PRISM): a pilot group randomized controlled trial of two approaches to improving depression among perinatal women. *Journal of Psychosomatic Obstetrics and Gynecology.* 2018;39(4):297-306.
5. Zhao YM-K, M. L.//Shi, S.//Wang, J.//Zhao, Q. Effects of antenatal depression screening and intervention among Chinese high-risk pregnant women with medically defined complications: A randomized controlled trial. *Early Intervention in Psychiatry.* 2019;13(5):1090-1098.

**Mixed population (e.g., those with/out existing depression) and does not provide disaggregated data (n=3)**

1. Fernandez y Garcia E, Joseph J, Wilson MD, Hinton L, Simon G, Ludman E, Scott F, Kravitz RL. Pediatric-based intervention to motivate mothers to seek follow-up for depression screens: The Motivating Our Mothers (MOM) trial. Academic pediatrics 2015; 15(3):311-318.
2. Edward KLG, J. A.//Stephenson, J.//Mills, C.//McFarland, J.//Castle, D. J. Self- screening using the Edinburgh post natal depression scale for mothers and fathers to initiate early help seeking behaviours. Arch Psychiatr Nurs. 2019;33(4):421-427.
3. George C. Effectiveness of a group intervention led by lay health workers in reducing the incidence of postpartum depression in South India. Asian Journal of Psychiatry. 2020;47 (no pagination)(101864).

**Study does not determine patient eligibility and randomize patients prior to administering the screening test (n=6)**

1. Kastello JC, Jacobsen KH, Gaffney KF, Kodadek MP, Bullock LC, Sharps PW. Self-Rated Mental Health: Screening for Depression and Posttraumatic Stress Disorder Among Women Exposed to Perinatal Intimate Partner Violence. Journal of Psychosocial Nursing and Mental Health Services 2015; 53(11):32-38.
2. Kubota C, Okada T, Aleksic B, Nakamura Y, Kunimoto S, Morikawa M, Shiino T, Tamaji A, Ohoka H, Banno N, Morita T, Murase S, Goto S, Kanai A, Masuda T, Ando M, Ozaki N. Factor structure of the Japanese version of the Edinburgh Postnatal Depression Scale in the postpartum period. PloS One 2014; 9(8):e103941.
3. Choi SK, Kim JJ, Park YG, Ko HS, Park IY, Shin JC. The simplified Edinburgh Postnatal Depression Scale (EPDS) for antenatal depression: is it a valid measure for pre-screening? International Journal of Medical Sciences 2012; 9(1):40-46.
4. Andrews Horowitz J, Murphy CA, Gregory KE, Wojcik J. Best practices: community-based postpartum depression screening: results from the CARE study. Psychiatric services (Washington, D.C.) 2009; 60(11):1432-1434.
5. [Grey Lit] Morrell CJ, Slade P, Warner R, Paley G, Dixon S, Walters SJ, Brugha T, Barkham M, Parry GJ, Nicholl J. Clinical effectiveness of health visitor training in psychologically informed approaches for depression in postnatal women: pragmatic cluster randomized trial in primary care. BMJ 2009; 338:a3045. **FOLLOW-UP FROM PROTOCOL
6. Zlotnick C, Miller IW, Pearlstein T, Howard M, Sweeney P. A preventive intervention for pregnant women on public assistance at risk for postpartum depression. The American Journal of Psychiatry 2006; 163(8):1443-1445.

**Intervention group does not receive an intervention that uses a single question, small sets of questions, or a screening questionnaire (validated or non-validated) with a pre-defined cut-off score to identify patients who may have depression, but who have not reported their symptoms to healthcare providers or who have otherwise not been identified as possibly depressed by healthcare providers (n=6)**

1. Fisher F, Rowe H, Wynter K, Tran T, Lorgelly P, Amir LH, Proimos J, Ranasinha S, Hiscock H, Bayer J, Cann W. Gender-informed, psychoeducational programme for couples to prevent postnatal common mental disorders among primiparous women: cluster randomised controlled trial. BMJ Open 2016; 6(3):e009396.
2. MacArthur C, Winter HR, Bick DE, Knowles H, Lilford R, Henderson C, Lancashire RJ, Braunholtz DA, Gee H. Effects of redesigned community postnatal care on womens' health 4 months after birth: a cluster randomised controlled trial Lancet 2002; 359(9304):378-385.
3. [Grey Lit] Shorey S, Wai Chi Chan S, Chong YS, He H-G. A randomized controlled trial of the effectiveness of a postnatal psychoeducation programme on self-efficacy, social support and postnatal depression among primiparas. Journal of Advanced Nursing 2014; 71(6):1260-1273. **FOLLOW UP FROM PROTOCOL
4. Poleshuck EW, M.//Crean, H. F.//Juskiewicz, I.//Bell, E.//Harrington, A.//Cerulli, C. A Comparative Effectiveness Trial of Two Patient-Centered Interventions for Women with Unmet Social Needs: Personalized Support for Progress and Enhanced Screening and Referral. Journal of Women's Health. 2020;29(2):242-252.
5. Yan WQ, Y.//Yan, F. Comprehensive nursing intervention effectively improves maternal psychological status and maternal postpartum quality of life. International Journal of Clinical and Experimental Medicine. 2019;12(9):11587-11594.
6. Ondersma SJB, J. R.//Puder, K. S.//Janisse, J.//Svikis, D. S. Feasibility and Acceptability of e-Screening and Brief Intervention and Tailored Text Messaging for Marijuana Use in Pregnancy. Journal of Women's Health. 2019;28(9):1295-1301.

**Control group received depression screening to test for acceptability/feasibility (n=1)**

1. Kingston D, Austin M-P, Veldhuyzen van Zanten S, Harvalik P, Giallo R, McDonald SD, MacQueen G, Vermeyden L, Lasiuk G, Sword W, Biringer A. Pregnant Women's Views on the Feasibility and Acceptability of Web-Based Mental Health E-Screening Versus Paper-Based Screening: A Randomized Controlled Trial. *Journal of Medical Internet Research* 2017; 19(4):e88.

**All participants in the control group receive screening and/or results are provided to patients or healthcare providers (n=8)**

1. Carrick-Sen DM, Steen N, Robson SC. Twin parenthood: the midwife's role--a randomised controlled trial. BJOG 2014; 121(10):1302-1311.
2. Paul IM, Downs DS, Schaefer EW, Beiler JS, Weisman CS. Postpartum anxiety and maternal-infant health outcomes. Pediatrics 2013; 131(4):e1218-e1224.
3. Robertson Blackmore E, Carroll J, Reid A, Biringer A, Glazier RH, Midmer D, Permaul JA, Stewart DE. The use of the Antenatal Psychosocial Health Assessment (ALPHA) tool in the detection of psychosocial risk factors for postpartum depression: a randomized controlled trial. Journal of Obstetrics and Gynaecology Canada 2006; 28(10):873-878.
4. Carroll JC, Reid AJ, Biringer A, Midmer D, Glazier RH, Wilson L, Permaul JA, Pugh P, Chalmers B, Seddon F, Stewart DE. Effectiveness of the Antenatal Psychosocial Health Assessment (ALPHA) form in detecting psychosocial concerns: a randomized controlled trial. Canadian Medical Association Journal 2005; 173(3):253-259.
5. Webster J, Linnane J, Roberts J, Starrenburg S, Hinson J, Dibley L. IDentify, Educate and Alert (IDEA) trial: an intervention to reduce postnatal depression. BJOG: 2003; 110(9):842-846.
6. Beck CT, Gable RK. Comparative analysis of the performance of the Postpartum Depression Screening Scale with two other depression instruments. Nursing Research 2001; 50(4):242-250.
7. Stamp GE, Williams AS, Crowther CA. Predicting postnatal depression among pregnant women. Birth 1996; 23(4):218-223.
8. Haga SMD, Filip//Lisoy, Carina//Wentzel-Larsen, Tore//Slinning, Kari. Mamma Mia-A randomized controlled trial of an internet-based intervention for perinatal depression. Psychological Medicine. 2019;49(11):1850-1858.

**The doctors in the control group are given a generic prompt which recommends that patients be screened for depression (n=1)**

1. Carroll AE, Biondich P, Anand V, Dugan TM, Downs SM. A randomized controlled trial of screening for maternal depression with a clinical decision support system. Journal of the American Medical Informatics Association 2013; 20(2):311-316.

**Study does not provide similar depression management and treatment resources to patients who were identified as depressed via screening in the screening arm of the trial and patients in either the screening or non-screening arms of the trial who were identified as depressed via other methods (n=2)**

1. Bobo WV, Wollan P, Lewis G, Bertram S, Kurland MJ, Vore K, Yawn Bp. Depressive symptoms and access to mental health care in women screened for postpartum depression who lose health insurance coverage after delivery: findings from the Translating Research into Practice for Postpartum Depression (TRIPPD) effectiveness study. Mayo Clinic Proceedings 2014; 89(9):1220-1228.
2. Barbara P Yawn, Allen J Dietrich, Peter Wollan, Susan Bertram, Debbie Graham, Jessica Huff, Margary Kurland, Suzanne Madison, Wilson D Pace, TRIPPD practices TRIPPD: a practice-based network effectiveness study of postpartum depression screening and management. Annals of family medicine 2012; 10(4):320-329.

## Additional file 10. List of ongoing trials in the pregnancy and postpartum population

| **Trial Identifier** | **Study Title** | **Study Start Date** | **Estimated Study Completion Date** |
| --- | --- | --- | --- |
| ACTRN 12616001217493 | Integrated e-Screening for Postnatal Depression and Anxiety- feasibility and effectiveness | Not reported | Not reported  (no publication available) |
